# Age-specific chikungunya outbreak response immunisation strategies in Brazil: a modelling study

**DOI:** 10.1016/j.eclinm.2025.103690

**Published:** 2025-12-05

**Authors:** Hyolim Kang, Ahyoung Lim, Andrew Clark, Felipe J. Colón González, Hannah Eleanor Clapham, Jean-Paul Carrera, Jong-Hoon Kim, Megan Auzenbergs, Preethi Lakshminarayanan, Sandra López-Vergès, So Yoon Sim, Su Myat Han, Thiago Cerqueira-Silva, Timothy Endy, Zulma M. Cucunubá, W John Edmunds, Sushant Sahastrabuddhe, Oliver J. Brady, Kaja Abbas

**Affiliations:** a Department of Infectious Disease Epidemiology and Dynamics, Faculty of Epidemiology and Population Health, London School of Hygiene & Tropical Medicine, London, UK; b School of Tropical Medicine and Global Health, Nagasaki University, Nagasaki, Japan; c Institute of Tropical Medicine, Nagasaki University, Nagasaki, Japan; d Department of Health Services Research and Policy, Faculty of Public Health and Policy, London School of Hygiene & Tropical Medicine, London, UK; e Data for Science and Health, Wellcome Trust, London, UK; f Saw Swee Hock School of Public Health, National University of Singapore and National University Health System, Singapore; g Carson Centre for Health and Ecosystems Research, Darien, Panama; h Department of Biology, University of Oxford, Oxford, UK; i International Vaccine Institute, Seoul, South Korea; j Imperial College London, London, UK; k Gorgas Memorial Institute for Health Studies, Panama City, Panama; l Department of Immunization, Vaccines, and Biologicals, World Health Organization, Geneva, Switzerland; m Laboratório de Medicina e Saúde Pública de Precisão - Fundação Oswaldo Cruz, Salvador, Brazil; n Coalition for Epidemic Preparedness Innovations, Washington, DC, USA; o Institute of Public Health, Pontificia Universidad Javeriana, Colombia; p Department of Microbiology, College of Medicine, Yonsei University, Seoul, South Korea; q Centre International de Recherche en Infectiologie, Université Jean Monnet, Université Claude Bernard Lyon 1, France; r Public Health Foundation of India, New Delhi, India; s National Institute of Infectious Diseases, Japan Institute for Health Security, Tokyo, Japan

**Keywords:** Chikungunya, Outbreak response immunisation, Vaccine impact modelling, Age-specific vaccination

## Abstract

**Background:**

Two chikungunya vaccines, Ixchiq and Vimkunya are licensed. In April 2025, Brazil is the first endemic country to license Ixchiq, but optimal age groups for vaccination remain unclear. Our aim is to model the public health impact of age-specific chikungunya outbreak response immunisation strategies in Brazil and infer broader implications for vaccine use case scenarios in outbreak prone regions.

**Methods:**

We developed an age-structured transmission dynamic model calibrated with state-level Brazilian surveillance data for 2022 and long-term average annual force of infections. We simulated outbreak response immunisation strategies targeting ages 1–11, 12–17, 18–59, and ≥60 years for Ixchiq and Vimkunya across 11 out of 27 states in Brazil. We assessed vaccine impact by symptomatic cases, deaths, and disability-adjusted life years (DALYs) averted and number needed to vaccinate (NNV) based on vaccine protection against disease only and against both disease and infection.

**Findings:**

Ixchiq and Vimkunya showed similar vaccine impact. Across strategies, vaccinating children 1–11 years yielded the lowest NNV for both vaccines, whereas vaccinating adults 18–59 years achieved the greatest absolute reduction in symptomatic cases, averting 62.5% (95% Uncertainty Intervals [UI]: 54.2–84.1) of total symptomatic cases with Vimkunya and 66.2% (58.2–86.0) with Ixchiq, under disease and infection blocking mechanism. Vaccinating adults 18–59 years with Ixchiq or Vimkunya yielded similar efficiency, with NNVs to avert a DALY of 339 (39–3412) and 361 (40–3777) respectively, under disease and infection-blocking mechanism.

**Interpretation:**

Under current licensure, vaccinating adolescents aged 12–17 years first, followed by 18–59 years are efficient strategies, with similar NNVs for both Ixchiq and Vimkunya. If eligibility expands to younger populations, vaccinating 1–11-year age group will have relatively higher efficiency.

**Funding:**

International Vaccine Institute and 10.13039/100009619Japan Agency for Medical Research and Development.

**Research in context**

**Evidence before this study**

We searched PubMed up to July 16, 2025, using the terms “chikungunya” AND (“vaccine” OR “vaccination” OR “immuni∗” OR “outbreak response” OR “reactive vaccination”) AND (“model∗” OR “impact”) in the title and abstract fields. We identified three modelling studies that assessed the impact of outbreak response immunisation strategies: one at the global level and two focused on Italy and Paraguay. These studies evaluated the population-level impact of outbreak response immunisation targeting adults aged 18 years and older using Ixchiq. Critical evidence gaps remain regarding optimal age-specific vaccination strategies for currently licensed chikungunya vaccines.

**Added value of this study**

To our knowledge, this is the first study to model age-specific vaccination strategies for two licensed chikungunya vaccines while aligning with current or potential licensure extensions for Ixchiq and Vimkunya. Specifically, we compared four age-specific outbreak response immunisation strategies for Ixchiq and Vimkunya, to guide optimal age group prioritisation under different vaccine protection mechanisms and potential label extensions to younger populations. We reconstructed subnational outbreaks from the 2022 Brazil epidemic with varying transmission dynamics and demographic structures. We inferred consistent patterns on efficiency metrics for number needed to vaccinate to avert health outcomes across transmission settings, suggesting broad applicability to outbreak prone regions for decision-making on age-group prioritisation for vaccination. We also calibrated reporting rates by comparing surveillance data with model-derived annual symptomatic burden derived from seroprevalence based force of infection estimates, revealing that the chikungunya burden is substantially higher than reported cases after adjusting for under-reporting. Our findings also provide broader implications for targeting optimal age groups for vaccination in outbreak prone regions beyond Brazil as well as for countries where vaccines are licensed for travellers to regions at risk of chikungunya outbreaks.

**Implications of all the available evidence**

We inferred that vaccinating children aged 1–11 years will have relatively higher efficiency if vaccine eligibility expands, with the lowest number needed to vaccinate to avert an infection, symptomatic case, death, or disability-adjusted life year, across transmission settings for Ixchiq and Vimkunya. Under current licensure approvals of Ixchiq and Vimkunya, vaccinating adolescents aged 12–17 years, followed by adults aged 18–59 years are efficient strategies under current licensures for both vaccines. Our findings also align with the current precautionary advice on chikungunya vaccine in UK travellers to not use the Ixchiq in adults aged 60 years and older, while the safety signal among older adults are reviewed. There are no similar age restrictions for Vimkunya among older adults though this vaccine has not been widely used. While longer-term evaluations are needed to assess vaccine effectiveness, our model-based inferences provide timely evidence on optimal age-specific vaccination strategies for Ixchiq and Vimkunya.

## Introduction

Chikungunya virus (CHIKV) is a mosquito-borne alphavirus transmitted by *Aedes albopictus* and *Aedes aegypti* mosquitoes. Since its first identification in 1952, outbreaks have been reported in over 115 countries across Asia, Africa, the Americas, and Europe.^1^ Although historically tropical, expanding vector habitats from viral adaptation, climate change, and globalisation have extended the geographical range of CHIKV beyond the tropics.^2^ Recent large-scale outbreaks in the Indian Ocean, the Americas and Southern Europe, such as those in La Réunion in 2025, Paraguay in 2023, and Italy in 2017 have demonstrated the epidemic potential of chikungunya beyond its traditional range.^2^, ^3^, ^4^, ^5^

CHIKV infection causes acute high fever, rash, myalgia and headache. While most acute symptoms resolve within one to two weeks, nearly 50% of patients develop chronic sequelae such as arthralgia or arthritis, persisting months to years and causing long-term disability.^6^ Case fatality is higher among older adults, particularly individuals aged 60 years and older.^7^ The risk of severe disease, including neurological disorders, is greater in children under 5 years.^8^

As of October 2025, two chikungunya vaccines (Ixchiq and Vimkunya) have received licensure, but key questions remain about vaccine protection mechanisms and real-world effectiveness across ages. Ixchiq, a live attenuated single-dose vaccine, and Vimkunya, a recombinant virus-like particle vaccine, received licensure via accelerated pathways. Ixchiq was the first chikungunya vaccine to receive regulatory approval, licensed by the US Food and Drug Administration (FDA) in 2023 (license suspended on August 22, 2025, based on vaccine-related serious safety concerns), and subsequently approved in the European Union (EU), Canada, UK, and Brazil.^9^, ^10^, ^11^, ^12^, ^13^, ^14^ Vimkunya received regulatory approvals in the US, EU, and UK in 2025.^15^, ^16^, ^17^ Initially approved for individuals aged 18 years and older, Ixchiq is approved for individuals aged 12 years and older by the European Medicines Agency (EMA) in April 2025, while the UK, Canada, and Brazil maintain licensure approval for individuals aged 18 years and older. Ixchiq was authorised based on a surrogate marker of protection established from animal challenge and sero-epidemiological cohort studies, although how this threshold translates to protection against CHIKV infection and disease in humans remains uncertain.^18^, ^19^, ^20^ Vimkunya also received US FDA accelerated approval following a phase 3 trial showing high neutralising antibody levels in participants ≥12 years, although antibody waning was observed after six months with different rates across age strata.^21^ Ongoing studies aim to clarify protection mechanisms in humans and expand vaccine eligibility to younger age groups. Pharmacovigilance surveillance and post-authorisation effectiveness studies are planned for Ixchiq.^22^ phase-3 trial for Ixchiq in Brazilian adolescents (12–17 years) showed a high-sustained sero-response, and phase-2 trial in children (1–11 years) in Dominican Republic and Honduras demonstrated antibody persistence, suggesting potential for further label extension to younger age groups.^23^^,^^24^

The first mass vaccination campaign using Ixchiq was launched in La Réunion in April 2025, initially targeting adults aged 18–64 with comorbidities and all adults aged 65 and over. Since its initial approval in November 2023 from the US FDA, over 80,000 doses of Ixchiq have been distributed globally as of October 2025.^25^ However, Ixchiq remains under regulatory scrutiny to assess its safety profile in real-world settings. Following reports of serious adverse events (SAEs), including two deaths among recipients aged 62–89 years in May 2025, EMA temporarily restricted vaccination in adults 65 and above and in immunocompromised individuals.^26^ The restriction has since been lifted in July 2025, but EMA now recommends vaccination only when there is significant risk of chikungunya infection and after careful consideration of the benefit-risk.^27^ The UK Commission on Human Medicines has temporarily restricted the use of Ixchiq for people aged 65 years and older due to rare fatal reactions, pending a safety review by the regulatory agency (Medicines and Healthcare Products Regulatory Agency in the UK), and the UK JCVI (Joint Committee on Vaccination and Immunisation) has advised against the use of Ixchiq in adults aged 60 years and older and immunocompromised individuals while safety review is ongoing.^28^^,^^29^ In the US, the FDA initially recommended pausing vaccination in those aged 60 years and older in May 2025. This pause was lifted on August 6, 2025.^30^^,^^31^ However, on August 22, 2025, the FDA suspended Ixchiq's license based on vaccine-related serious safety concerns.^9^ This evolving regulatory landscape underscores that post-marketing surveillance is crucial to comprehensively evaluate chikungunya vaccine safety across different populations and transmission settings, particularly considering underlying health conditions of vaccine recipients and age-specific risk profiles.

Despite the availability of licensed chikungunya vaccines, few studies have assessed the potential impact of outbreak response immunisation at national, regional, or global levels.^32^, ^33^, ^34^ Critical gaps remain, particularly regarding age-specific prioritisation aligned with current licensure for targeted age groups or anticipated future eligibility extensions to younger age groups. Moreover, recent safety concerns associated with Ixchiq have led to age-based restrictions in several countries, underscoring the need to evaluate the impact across different age-groups.

To address this evidence gap, we compared four age-specific vaccination strategies at national and sub-national levels under conservative (protection against disease only) and optimistic (protection against both infection and disease) vaccine protection mechanisms and varying coverage levels. We developed a transmission dynamic model integrated with surveillance data and seroprevalence-derived force of infection (FOI) data. We estimated the efficiency of each strategy by the number needed to vaccinate (NNV) to avert an infection, symptomatic case, death, or disability-adjusted life year (DALY). We infer useful insight from our modelling study for policy and programmatic decision-making on age-specific outbreak response immunisation strategies.

## Methods

### Chikungunya transmission dynamic model

We developed an age-structured SEIRV (Susceptible, Exposed, Infectious, Recovered, Vaccinated) transmission dynamic model to reconstruct chikungunya outbreaks in Brazilian states (Level 1 administrative units, hereby *state*) (Figure S1). We ran the model over 52 weeks to assess outbreak response immunisation strategies.

We selected the 2022 chikungunya outbreak for its high reporting completeness (52 epidemiological weeks across affected states) and wide geographic spread across all 27 states. We excluded states with insufficient epidemic signal (peak symptomatic cases <20 per million population or total symptomatic cases <500), as these exhibited stochastic fluctuations rather than identifiable outbreak dynamics. Among the remaining states, we performed k-means clustering using peak symptomatic cases per million and total cases to examine the empirical clustering pattern of the states. Based on this, we categorised states with peak symptomatic cases per million population of (i) less than 100 as low transmission setting, (ii) 100–200 as moderate, and (iii) > 200 as high transmission setting (Table S3, Figures S2–S4). We did not explicitly include vector compartments; instead, mosquito-to-human transmission dynamics was incorporated into a weekly transmission rate $\left(\beta_{t}\right)$, embedding entomological and ecological factors. We defined force of infection as $\beta_{t}$ ∗ $\frac{I_{t}}{N}$ and applied uniformly across age groups. We did not include ageing due to the short outbreak duration (<1 year).

Our modelling process included calibration of epidemiological parameters, simulation of age-specific vaccination strategies, and estimation of health impact by strategy. We modelled two licensed vaccines (Ixchiq and Vimkunya) and two vaccine protection mechanisms: (i) disease blocking only —protection against disease (preventing symptomatic disease, deaths, and DALYs) but not against infection, and (ii) disease and infection blocking —protection against both infection and disease at the same efficacy level, as reported in phase 3 trials. We assumed both vaccines are all-or-nothing vaccines based on animal challenge studies suggesting sterile immunity in non-human primates and phase-3 trials. From the passive transfer challenge experiments, macaques showed no detectable replicating wild-type CHIKV across a wide range of transferred titres. This suggests an all-or-nothing mechanism of vaccine protection, in the absence of human challenge or vaccine effectiveness studies.^18^^,^^20^^,^^21^^,^^35^

### Chikungunya data

We used reported chikungunya case data and seroprevalence-derived FOI data to leverage complementary strengths of both data types.^36^ We sourced weekly age-stratified confirmed chikungunya case data from the Brazilian National Notifiable Disease Information System (Sistema de Informação de Agravos de Notificação—SINAN) for January–December 2022,^37^ and population data from the 2022 Brazilian census (ages 0–89, 1-year interval).^38^ Reported case data provide timely burden estimates but are subject to underreporting.^36^ We used model-predicted annual symptomatic cases and multi-year average reported cases (2015–2024) to calibrate state-specific reporting rates^39^ (Tables S1–S2).

### Model parameters and calibration

We calibrated weekly transmission rates $\left(\beta_{t}\right)$, latent period (σ), recovery rate (γ), initial infections $\left(I_{0}\left[a\right]\right)$, and reporting rate $\left(\rho_{s}\right)$. Weekly transmission rates, initial infections, and reporting rates were calibrated per state, while latent period and recovery rate were common across all states (Supplementary p2-3, Figures S5–S6). To represent baseline immunity from past-infections, we estimated individuals with prior natural infection and recovery using long-term annual FOI estimates for each state. We estimated age-specific equilibrium seroprevalence from FOI using a catalytic model, $1-e^{-FOI*age}$, assuming constant FOI and lifelong immunity.^6^ We estimated the long-term national reporting rate as 25% (95% Uncertainty Intervals [UI]: 20.1–32.5), calculated from the ratio between the national long-term average of notified cases (2015–2024) and the long-term average of model-predicted symptomatic cases derived from the predicted FOI maps.^39^ We used this estimate for an informative prior (beta distribution) for the reporting rate $\rho_{s}$. We fitted data from each state to update $\rho_{s}$ through posterior distribution, thereby capturing spatial heterogeneity in reporting rates (Supplementary p4, Figure S6). We fitted the model to 2022 weekly reported data using Stan's Hamiltonian Monte Carlo algorithm with the No-U-Turn Sampler (5000 iterations; 1000 burn-in). We assessed model convergence using effective sample size and R-hat (<1.01 indicating convergence) (Table S5).

We estimated symptomatic cases from predicted infections by applying symptomatic progression and state-specific reporting rates calibrated from our model.^40^ We used hospitalisation rates and case-fatality rates by each age group and estimated age-specific DALYs. We used Latin Hypercube sampling (1000 iterations) to draw from the uncertainty distributions of disability weights, illness duration, and remaining life-years to estimate DALYs with 95% UIs (uncertainty intervals) (Table S4).^39^ All analyses were conducted using R.4.3.3, and the software code is publicly available on GitHub (https://github.com/hyolimkang/CHIK_VIM).

### Vaccine efficacy, vaccination strategies and outcome measures

In comparison to no vaccination, we evaluated four age-specific vaccination strategies: (i) 1–11 years, (ii) 12–17 years, (iii) 18–59 years, and (iv) ≥ 60 years. These strategies reflect current vaccine licensure for different age groups, plus considering potential label extension to younger children.

We derived vaccine efficacy and time until immunity acquisition from phase-3 trial results. For Ixchiq, we applied 98.9% (95% CI: 96.7–99.8) efficacy for all age groups, with immunity established two weeks post-vaccination and no waning.^20^ For Vimkunya, we applied pre-waning efficacy 97.8% (95% CI: 97.2–98.3) at 3-weeks post-vaccination for all individuals, and assumed exponential waning, reaching 94.8% for 12–17 years, 85% for 18–45 years, 84.1% for ≥45 years by week 26 based on phase-3 trial data. We applied the same weekly exponential waning rate (ω) beyond week 26 (Equation (1)).^21^ We modelled two vaccine protection mechanisms — one assuming disease blocking only, and the other assuming both disease and infection blocking at the same efficacy.

(1) $$VE\left(t\right)=VE_{week3}*e^{-\omega \;\left(t-3\right)}$$

We assumed vaccination began two weeks after outbreak onset, delivering vaccines at 10% of the target population per week (delivery speed), with 50% of coverage for target population (Table 1) — vaccine coverage was varied between 10 and 100% and deployment delay between 1 and 52 weeks in the uncertainty analysis. We determined vaccination windows based on weekly delivery speed (Figures S6–S7). We estimated health impact of each vaccination strategy using the absolute and proportional reduction in cumulative symptomatic cases, deaths, and DALYs over a 1-year horizon. Absolute impact was defined as the difference in chikungunya burden (cases, deaths, and DALYs) between the vaccination scenario and the counterfactual no-vaccination scenario, and proportional reduction was calculated as the percentage of baseline burden averted. To assess efficiency, we calculated NNV to avert a symptomatic case, death, or DALY, and also expressed as averted burden per 100,000 doses. All outcomes were summarised using medians and 95% uncertainty intervals (UIs) to illustrate the uncertainty.

**Table 1:** Model parameters.

	Ixchiq	Vimkunya	Reference
**Vaccine and vaccination parameters**			
Vaccine efficacy (pre-waning)	98.9% (95% CI: 96.7–99.8)	97.8% (95% CI: 97.2–98.3)	^20^^,^^21^
Vaccine efficacy (post-waning—26 weeks after vaccination)	–	12–17 years: 94.8% 18–45 years: 85% >45 years: 84.1%	^20^^,^^21^
Time until acquisition of immunity (weeks)	2	3	^20^^,^^21^
Vaccine coverage	50% (95% UI: 40–60)		Assumption^a^
Weekly vaccine delivery rate	10% (95% UI: 0.09–0.11)		Assumption
Delay in deployment (weeks)	2 (95% UI: 1–3)		Assumption^b^
**Disease parameters**			
Recovery rate (week)	0.54 (95% UI: 0.50–0.58)		^41^
Latent period (week)	0.60 (95% UI: 0.45–0.80)		^42^
Proportion of infections progressing to symptomatic cases	52.40% (95% UI: 40.6–64.0)		^40^
**Long term average FOI**			
**High transmission setting**			
Ceará	0.012 (95% UI: 0.005–0.026)		^39^
Piauí	0.012 (95% UI: 0.005–0.026)		^39^
Paraíba	0.011 (95% UI: 0.005–0.022)		^39^
Alagoas	0.008 (95% UI: 0.003–0.018)		^39^
**Moderate transmission setting**			
Tocantins	0.009 (95% UI: 0.004–0.022)		^39^
Pernambuco	0.009 (95% UI: 0.004–0.021)		^39^
**Low transmission setting**			
Bahia	0.009 (95% UI: 0.004–0.020)		^39^
Rio Grande do Norte	0.011 (95% UI: 0.005–0.025)		^39^
Minas Gerais	0.012 (95% UI: 0.005–0.024)		^39^
Sergipe	0.008 (95% UI: 0.004–0.017)		^39^
Goiás	0.008 (95% UI: 0.003–0.020)		^39^

Parameters governing the estimation of chikungunya transmission dynamics and vaccination impact—baseline values & 95% uncertainty intervals for sensitivity analyses.

a Vaccine coverage is varied 10–100% in additional sensitivity analysis (see Figure S13).

b Delay in deployment is varied 1–52 weeks in additional sensitivity analysis (see Figure S13).

### Uncertainty analysis

We performed both multivariate probabilistic sensitivity analysis and one-way deterministic sensitivity analyses. For probabilistic sensitivity analysis, we drew 1000 probabilistic values from uncertainty distribution of parameters via Latin Hypercube Sampling and incorporated full uncertainty of FOIs in each state derived from predicted global FOI maps in our previous study (Supplementary p. 16).^39^ We quantified the results using medians and 95% UIs from 1000 simulations (optimal to reach convergence). We conducted one-way deterministic sensitivity analysis to assess individual parameter influence on vaccine impact measured as percent reduction in cumulative symptomatic cases (Figures S17 and S18). We also performed an extended grid-search scenario analysis by varying vaccine coverage (10–100% by 10%) and deployment delay (1–52 weeks by 1 week), estimating percent reduction of cumulative symptomatic cases and proportion of indirectly averted cases for each combination of coverage and delay (Figure S19).

### Ethics

Since we used publicly available data without individual-level identifiers and were already anonymised and in the public domain, ethical approval was not required.

### Role of funding source

The funder (IVI) of the study had a role in study design, data collection, data analysis, data interpretation, and writing of the report.

## Results

Of the 27 states in Brazil, 11 were included in our model for outbreak response immunisation (Figures S2–S4). Before vaccination, our model predicted 2.00 million (95% uncertainty interval [UI] 0.29M–13.8 M) infections, 1.05 million (0.15M–7.5 M) symptomatic cases, 837 (122–6077) deaths, and 109,483 (16,281–770,187) DALYs across these states, adjusting for underreporting. Adults aged 18–59 years accounted for the largest proportion of symptomatic cases (60.7%), while children aged 1–11 years accounted for the largest proportion of deaths (38.1%). Calibrated reporting rates ranged from 3% (2–5) in Alagoas to 22% (17–29) in Bahia.

**Fig. 1:**
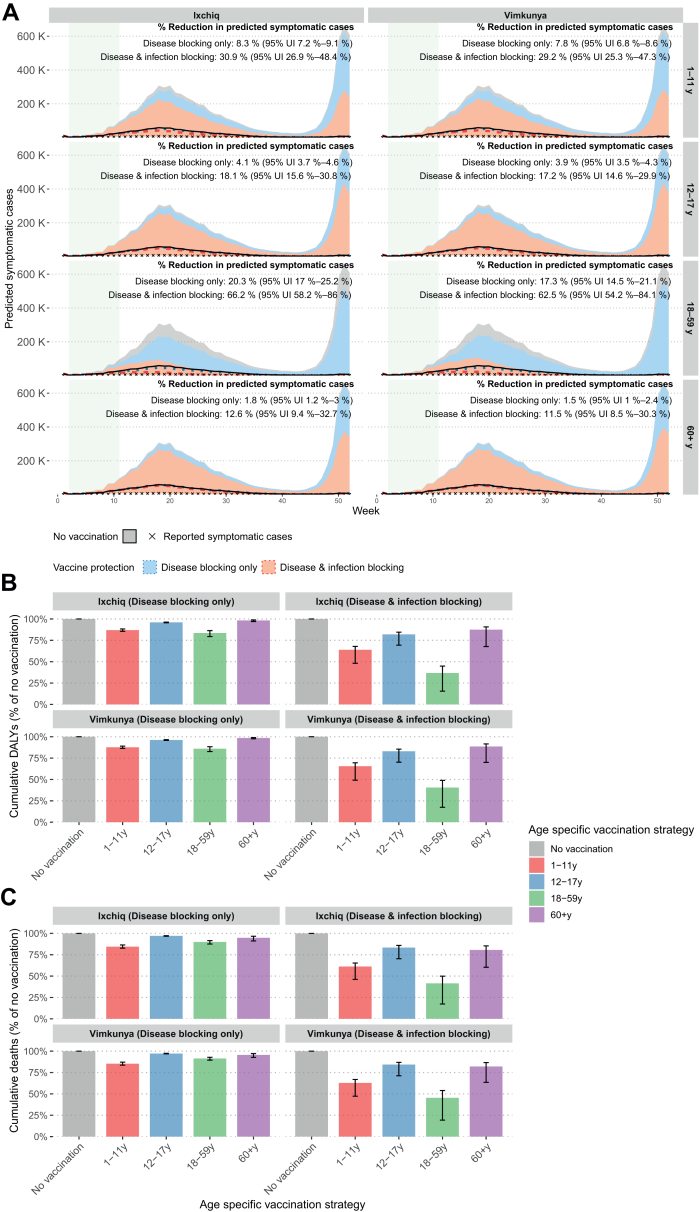
**Impact of chikungunya outbreak response immunisation strategies on predicted symptomatic cases, deaths, and DALYs at the national level in Brazil.** Panel A shows national-level epidemic curves (median and 95% uncertainty intervals) of predicted symptomatic cases for four age-specific vaccination strategies at 50% vaccination coverage and the counterfactual of no vaccination, aggregated across 11 states for Ixchiq and Vimkunya for two vaccine protection (disease-blocking only and disease & infection blocking) mechanisms. The light green shaded area in Panel A indicates the vaccination period (weeks 2–11). Panel B shows percent reduction in cumulative DALYs by age-specific vaccination strategy at the national level. Panel C shows percent reduction in cumulative deaths by age-specific vaccination strategy at the national level.

Overall vaccine impact varied notably by vaccine protection mechanism and targeted age group but minimally between Ixchiq and Vimkunya. Vaccinating 18–59 years averted the most symptomatic cases due to relatively higher number of effectively vaccinated people, with 50% vaccine coverage across the strategies. When the vaccines protect against disease only, the impact from vaccinating 18–59 years was 17.2% (14.6–29.9) with Vimkunya and 18.1% (15.6–30.8) with Ixchiq. Vaccinating children aged 1–11 years achieved the next greatest impact, averting 7.8% (6.8–8.6) with Vimkunya and 8.3% (7.2–9.1) with Ixchiq. Vaccinating adults aged ≥60 years resulted in relatively lower impact, averting 1.5% (1–2.4%) with Vimkunya and 1.8% (1.2–3) with Ixchiq. When the vaccines protect against both disease and infection, the impact increased due to indirect effects and herd immunity. For instance, vaccinating 18–59 years averted 62.5% (54.2–84.1) of symptomatic cases with Vimkunya and 66.2% (58.2–86) with Ixchiq (Fig. 1A).

Vaccine impact on reduction in DALYs and deaths was also similar between Ixchiq and Vimkunya. When the vaccines protect against disease only, vaccinating 18–59 years produced the largest reduction in DALYs, averting 14.0% (11.7–17.3) with Vimkunya and 16.4% (13.7–20.7) with Ixchiq. When the vaccines protect against both disease and infection, the impact on reduction in DALYs increased to 59.5% (51.0–82.5%) with Vimkunya and 63.3% (55.1–84.5%) with Ixchiq (Fig. 1B). In terms of total deaths averted, when the vaccines protect against disease only, vaccinating 1–11 years had the greatest impact, averting 14.7% (12.8–16.4%) with Vimkunya and 15.6% (13.6–17.3%) with Ixchiq. However, when vaccines protect against both disease and infection, the highest impact shifted to vaccinating 18–59 years, averting 54.6% (46.0–80.7%) with Vimkunya to 58.5% (50.1–82.7%) with Ixchiq, followed by vaccinating 1–11 years (Fig. 1C).

At the state level, we observed substantial variation in impact, but patterns across vaccination strategies were consistent with the national level trends. Overall vaccine impact was relatively higher in high-transmission settings than in low-transmission settings. For example, the reduction in total symptomatic cases from vaccinating 18–59 years ranged from 41% (19–58) in Goiás, a low transmission state, to 82% (68–88) in Alagoas, a high-transmission state (Figures S13 and S14).

**Fig. 2:**
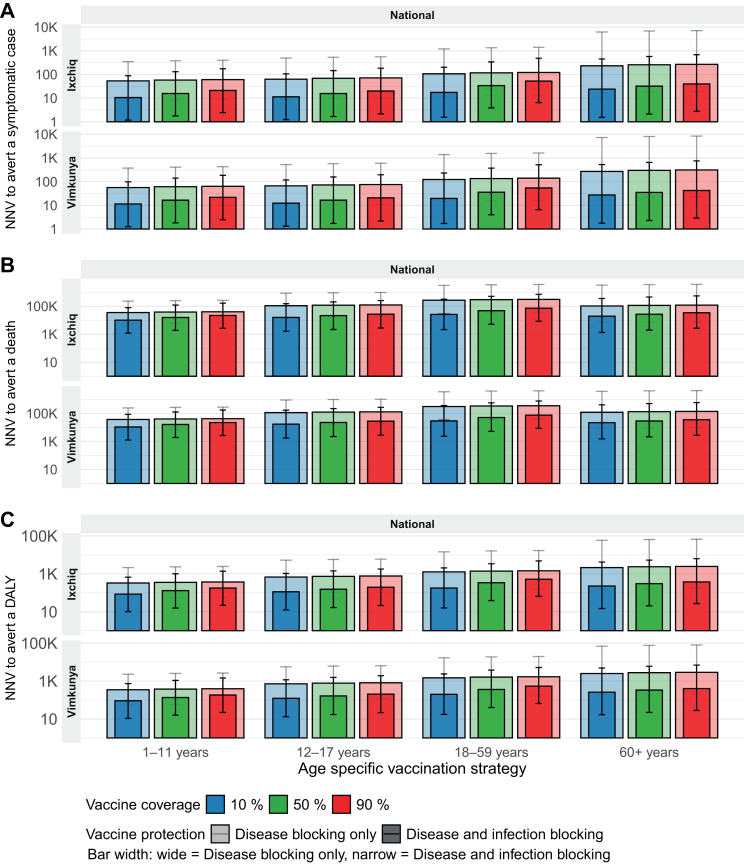
**Efficiency of chikungunya outbreak response immunisation strategies.** Number needed to vaccinate (NNV) to avert a symptomatic case (Panel A), death (Panel B), or DALY (Panel C) by age-specific vaccination strategy, vaccine protection (disease blocking only, and disease and infection blocking), and vaccine product (Vimkunya, Ixchiq), at different coverage levels (10%, 50%, 90%). Results represent national level average across 11 states in Brazil. Darker coloured bars indicate NNV for disease and infection blocking vaccine protection, and lighter colour bars indicate NNV for disease-blocking only vaccine protection. Subnational NNV graphs for each health outcome are provided in Figures S8–S9.

Across health outcomes, NNVs to avert symptomatic cases, deaths, and DALYs was lowest when vaccinating 1–11 years, for both vaccines and protection mechanisms (Fig. 2). That is, relatively higher number of symptomatic cases, deaths, and DALYs were averted per 100,000 doses by vaccinating 1–11 years (Table 2). Although overall vaccine impact was greatest when targeting larger population age-group (18–59 years), NNV to avert a health outcome was relatively lower when vaccinating 1–11 years. When the vaccines protect against disease only, vaccinating 1–11 years required the fewest doses to avert a symptomatic case, with NNVs of 58 (9–381) for Ixchiq and 61 (9–411) for Vimkunya at 50% vaccine coverage. When the vaccines protect against both disease and infection, the NNV decreased to 16 (2–131) for Ixchiq and 17 (2–158) for Vimkunya (Fig. 2A–Table 2).

**Table 2:** Efficiency of chikungunya outbreak response immunisation strategies.

Vaccine protection	Vaccine impact	Vimkunya				Ixchiq			
		Age-specific vaccination strategy							
		1–11 years	12–17 years	18–59 years	60+ years	1–11 years	12–17 years	18–59 years	60+ years
Disease blocking only	Infections averted per 100,000 doses	–	–	–	–	–	–	–	–
	Symptomatic cases averted per 100,000 doses	1635 (244–10,541)	1376 (173–9577)	739 (64–6430)	333 (12–4475)	1727 (263–11,151)	1454 (187–10,119)	856 (74–7814)	390 (15–5522)
	Deaths averted per 100,000 doses	2.47 (0.37–15.90)	0.80 (0.10–5.54)	0.29 (0.03–2.58)	0.74 (0.02–10.57)	2.61 (0.40–16.82)	0.84 (0.11–5.85)	0.34 (0.03–3.13)	0.87 (0.03–13.04)
	DALYs averted per 100,000 doses	265 (40–1709)	128 (16–892)	62 (5–542)	36 (1–491)	280 (43–1808)	135 (17–943)	72 (6–659)	42 (2–605)
	NNV (Infection)	–	–	–	–	–	–	–	–
	NNV (symptomatic case)	61 (9–411)	73 (10–578)	135 (16–1563)	300 (22–8061)	58 (9–381)	69 (10–534)	117 (13–1346)	257 (18–6841)
	NNV (death)	40,558 (6290–272,247)	125,656 (18,055–998,760)	342,054 (38,812–3,988,925)	135,075 (9459–4,147,192)	38,385 (5946–252,361)	118,957 (17,088–924,187)	295,296 (31,937–3,434,067)	115,507 (7667–3,516,908)
	NNV (DALY)	377 (59–2528)	780 (112–6198)	1617 (185–18,847)	2772 (204–76,284)	357 (55–2343)	738 (106–5736)	1396 (152–16,226)	2369 (165–64,734)
Disease and infection blocking	Infections averted per 100,000 doses	10,265 (1130–94,024)	10,306 (1032–100,220)	4922 (462–43,836)	5093 (275–77,645)	10,904 (1240–97,038)	10,974 (1136–103,658)	5249 (513–45,449)	5604 (313–83,724)
	Symptomatic cases averted per 100,000 doses	6054 (708–54,988)	6040 (632–58,092)	2794 (267–25,042)	2837 (153–43,812)	6385 (766–56,654)	6394 (688–59,979)	2961 (294–25,881)	3120 (174–47,389)
	Deaths averted per 100,000 doses	6.11 (0.78–51.81)	4.39 (0.45–44.32)	1.94 (0.17–18.76)	3.44 (0.20–46.99)	6.37 (0.83–53.10)	4.66 (0.49–45.83)	2.08 (0.20–19.50)	3.72 (0.22–50.70)
	DALYs averted per 100,000 doses	737 (92–6238)	608 (64–5822)	277 (26–2490)	299 (17–4516)	771 (99–6405)	645 (70–6014)	295 (29–2579)	329 (19–4883)
	NNV (Infection)	10 (1–89)	10 (1–97)	20 (2–216)	20 (1–364)	9 (1–81)	9 (1–88)	19 (2–195)	18 (1–319)
	NNV (symptomatic case)	17 (2–141)	17 (2–158)	36 (4–374)	35 (2–652)	16 (2–131)	16 (2–145)	34 (4–340)	32 (2–574)
	NNV (death)	16,361 (1930–128,998)	22,805 (2256–223,690)	51,520 (5331–571,583)	29,089 (2128–509,033)	15,704 (1883–121,153)	21,437 (2182–204,153)	48,055 (5129–511,670)	26,849 (1972–456,197)
	NNV (DALY)	136 (16–1083)	164 (17–1557)	361 (40–3777)	334 (22–6025)	130 (16–1011)	155 (17–1426)	339 (39–3412)	304 (20–5307)

Estimated national-level vaccine impact at 50% vaccine coverage on infections, symptomatic cases, deaths, and disability-adjusted life years (DALYs) averted per 100,000 doses by age-specific vaccination strategy, vaccine protection (disease blocking only, and disease and infection blocking), and vaccine product (Vimkunya, Ixchiq) and number needed to vaccinate (NNV) to avert infection, symptomatic case, death, and DALY.

Vaccinating 1-11-year age group required the fewest vaccine doses to avert a death, followed by vaccinating 12–17 years, ≥60 years, and 18–59 years, across both vaccines and protection mechanisms. With vaccine protection against disease only at 50% coverage, vaccinating 1–11-year age group required 38,385 doses (5946–252,361) for Ixchiq and 40,558 doses (6290–272,247) for Vimkunya to avert a death. When vaccines protect against both disease and infection, the NNV decreased to 15,704 (1883–121,153) for Ixchiq and 16,361 (1930–128,998) for Vimkunya (Fig. 2B, Table 2).

Vaccinating 1-11-year age group also achieved the lowest NNV to avert a DALY. When vaccines protect against disease only, the NNV to avert a DALY was 357 (55–2343) for Ixchiq and 377 (59–2528) for Vimkunya. The NNV further improved when the vaccines protect against both disease and infection, with NNV decreasing to 130 (16–1011) for Ixchiq and 136 (16–1083) for Vimkunya. Notably, when the vaccines protect against both disease and infection, vaccinating ≥60 years was more efficient than vaccinating 18–59 years to avert a DALY. For Ixchiq, the NNV was 304 (20–5307) when vaccinating ≥60 years, compared with 339 (39–3412) for 18–59 years, with a similar pattern observed for Vimkunya (Fig. 2C, Table 2).

The NNVs to avert symptomatic case, death, and DALY remained constant across different coverage levels (10%, 50%, 90%) when the vaccines protect against disease only. In contrast, when the vaccines protect against both disease and infection, the NNVs increased by coverage level, reflecting smaller marginal gains from indirectly averted health outcomes at higher coverage. Overall, the pattern of NNVs across vaccination strategies was consistent across transmission settings. For symptomatic cases and DALYs, vaccinating 1-11-year age group had the lowest NNVs, followed sequentially by 12–17 years, 18–59 years, and ≥60 years. For deaths, vaccinating 1-11-year age group had the lowest NNV, followed by 12–17 years, ≥60 years, and 18–59 years (Figures S10 and S11). Overall, Ixchiq and Vimkunya showed comparable vaccine impact and efficiency.

Based on the one-way sensitivity analysis, we identified underlying immunity derived from long-term FOI, and total vaccine coverage as the principal drivers of uncertainty in vaccine impact (Figures S17 and S18). The dominant driver of uncertainty differed by age-specific vaccination strategy. For strategies vaccinating adults (≥18 years), the percent reduction in total symptomatic cases was most sensitive to underlying immunity, whereas for strategies targeting children (1–11 years) and adolescents (12–17 years), the vaccine impact was most sensitive to vaccine coverage, followed by underlying immunity. Despite the differences in sensitivity, the relative ranking of vaccination strategies remained unchanged.

## Discussion

Based on our model-based projections, we infer that vaccinating children aged 1–11 years is the relatively efficient strategy to reduce symptomatic cases, deaths, and DALYs for both currently licensed vaccines —Ixchiq and Vimkunya. Under current licensure, vaccinating adolescents aged 12–17 years first, followed by 18–59 years are efficient strategies, with similar NNVs for both Ixchiq and Vimkunya. If eligibility expands to younger populations, vaccinating 1–11-year age group will be more efficient. However, given the emerging safety concerns that restrict the use of Ixchiq in adults aged 60 years and older in the UK, and the suspension of its licensure in the US, our findings on the benefits of chikungunya vaccination should be interpreted alongside a benefit-risk assessment for decision-making on vaccination strategies.

Given uncertainty around vaccine-induced protection, our study provides timely evidence to guide age-based vaccination strategies. We evaluated vaccine protection against disease only and against both disease and infection, as well as considering children not yet eligible for vaccination. Anticipating expanded eligibility to younger age groups, our study contributes to ongoing clinical and policy discussions by quantifying potential population-level impact and informing age-specific vaccination strategies.

The first mass vaccination campaign with Ixchiq in La Réunion in April 2025 highlighted how safety concerns limit vaccine eligibility. Vaccination initially targeted adults aged 65 years and above and 18–64-years old with comorbidities as well as professionals working in vector control based on French NITAG recommendation.^43^ Following serious adverse events in recipients over 60 years of age, the US FDA advised temporary restriction of Ixchiq use in adults aged 60 and older on May 9, 2025.^30^ The restriction was removed on August 6, 2025, but the license was suspended on August 22, 2025, by the US FDA based on vaccine-related serious safety concerns.^9^^,^^31^

Our findings provide model-based assessment of the impact and efficiency of Vimkunya, currently licensed for individuals 12 years and older with potential label extension to younger age groups. Specifically, Vimkunya showed similar efficiency to Ixchiq across all age-groups in terms of NNV to avert symptomatic cases, deaths, and DALYs. However as of October 2025, Vimkunya has not been used extensively.^29^

Our modelling study offers useful insight for chikungunya vaccine policy and programmatic decision-making by reconstructing subnational outbreaks across diverse transmission settings, showing consistent implications for age-based prioritisation under varying transmission and demographic conditions. While earlier studies evaluated outbreak response immunisation, our study is the first to stratify strategies by current and potential licensure age groups and to incorporate age-stratified disease parameters and state-specific underlying immunity using predicted FOIs.^32^, ^33^, ^34^ Our results show that uncertainty in vaccine impact largely arises from uncertainty in underlying immunity and underscores the need for strengthening surveillance, as calibrated reporting rates were low and varied across states. This suggests that the disease burden of chikungunya epidemics may be substantially underestimated.

Our study has limitations. First, with a one-year horizon, we could not evaluate whether protection persists beyond the first year or how transmission evolves later. Considering waning sero-responses observed in phase-3 trial for Vimkunya at six months after vaccination, future studies should extend follow-up over a longer time-horizon to examine durability of protection and need for booster doses. Second, we did not account for individual-level risk factors such as comorbidities or occupational exposures, as considered during the vaccine deployment to control the chikungunya outbreak in La Réunion in April 2025. As such, our model-based results should be interpreted with caution, as impact may differ when comorbidities are included. Future analyses could integrate individual-level risk stratification as more data become available. Third, our analysis is limited by the retrospective selection of states, driven by the unpredictable outbreak trajectories. Future modelling studies should develop locally tailored outbreak response triggers with local stakeholders. In the absence of such thresholds, our results highlight trade-off between early vaccine deployment without precise outbreak definition and reduced impact resulting from delayed deployment. Fourth, our model relied on immunogenicity biomarker to characterise vaccine protection.^20^^,^^21^ However, uncertainty remains regarding how well this immunogenicity biomarker is correlated with clinical protection. Our current results are therefore based on available correlates, and the magnitude of impact may vary depending on actual real-world clinical protection. Fifth, our model did not explicitly include mosquito compartments but captured human-mosquito-human transmission through calibrated weekly transmission rates. Given the absence of reliable entomological data at the subnational level, we adopted a parsimonious modelling approach, as adding limited vector parameters would not improve parameter identifiability and could introduce additional uncertainty.^44^ While an explicit vector model could introduce delayed changes in FOI, our main conclusions regarding the relative effectiveness of age-specific vaccination strategies would remain consistent, as these strategic comparisons depend primarily on transmission intensity and immunity rather than fine-grained vector dynamics.^45^

Current recommendations in high-income settings without local CHIKV transmission focus on individual-level protection for travellers, whereas our study addresses broader population-level impact in outbreak prone regions. In such non-endemic settings, importation may play a substantial role, and future models could assess the benefits of pre-travel vaccination in preventing autochthonous transmission.^46^^,^^47^ While our model did not explicitly incorporate importation or inter-state mobility, these factors are unlikely to substantially alter age-targeting priorities for outbreak response immunisation. Once sustained local transmission is established, epidemic progression is primarily driven by local ecological and demographic factors rather than importation patterns.^48^

While vaccinating children aged 1–11 years was predicted to have relatively higher efficiency (lowest NNV) to avert symptomatic cases and DALYs across outbreak settings, our findings should be interpreted as comparative epidemiological impact rather than cost-effectiveness of the vaccines. In practice, vaccination strategies targeting children versus adults may differ in terms of feasibility, logistical complexity, and public acceptability which should be considered when making programmatic decisions. Future research should extend these findings by evaluating age-specific cost-effectiveness, considering direct medical costs and indirect costs, such as long-term productivity loss.

In conclusion, we evaluated health impact and efficiency of age-specific chikungunya outbreak response immunisation strategies, comparing two licensed vaccines (Ixchiq and Vimkunya) based on vaccine protection against disease only and against both disease and infection. While grounded in Brazilian context, our results provide broader insights for decision-making on optimal age-specific vaccination strategies for outbreak prone regions in Brazil and beyond, while accounting for future licensure approvals for adolescents and children.

## Contributors

HK, OJB, and KA conceptualised the study. HK conducted all analyses, coding, visualisation, and wrote the original draft. AL, AC, FJCG, HEC, JPC, JK, MA, PL, SLV, SYS, SMH, TCS, YE, ZMC, WJE, and SS contributed to the review and editing of the manuscript. All authors had access to the study dataset, which was verified by HK, OJB, and KA. All authors reviewed and approved the final version of the manuscript. The authors alone are responsible for the views expressed in this article, which do not necessarily reflect the views, decisions or policies of their affiliated organisations or the study funders. HK is the guarantor for the overall content of this article.

## Data sharing statement

All data supporting our findings are available in the public, open access repository (https://github.com/hyolimkang/CHIK_VIM).

## Declaration of interests

We declare no competing interests.
