# Supplementary material for: Age-specific chikungunya outbreak response immunisation strategies in Brazil: a modelling study
Source: eClinicalMedicine. 2025 Dec 5;90:103690. doi: 10.1016/j.eclinm.2025.103690 (PMC12766480; doi:10.1016/j.eclinm.2025.103690)
Supplement: Supplementary Figures and Tables [file mmc1.docx]

Modelling age-specific chikungunya outbreak response immunisation strategies in Brazil: broader implications for vaccine use case scenarios

**Supplementary materials**

**Figures**

[Supplementary figure 1 Model diagram and equation 3](#_Toc212121916)

[Supplementary figure 2 Reported chikungunya outbreak case data in Brazil by state (2022) 6](#_Toc212121917)

[Supplementary figure 3 Total case and peak symptomatic cases (per million) distribution and state classification 7](#_Toc212121918)

[Supplementary figure 4 Total cumulative reported cases and per capita cases by state in Brazil (2022). 8](#_Toc212121919)

[Supplementary figure 5 Model calibrated weekly transmission rates with 95% UI by sub-national state 10](#_Toc212121920)

[Supplementary figure 6 Predicted reporting rates by state (median and 95%UI) 11](#_Toc212121921)

[Supplementary figure 7 Model fitted epidemic curve with reported cumulative case data in Brazil (2022) by state 12](#_Toc212121922)

[Supplementary figure 8 Age-specific pre-vaccination symptomatic cases per million population 13](#_Toc212121923)

[Supplementary figure 9 DALY reduction from each age-specific vaccination strategy by component (acute, sub-acute, chronic YLDs, and YLL) 14](#_Toc212121924)

[Supplementary figure 10 Direct and indirect effects of vaccination strategy at national and sub-national levels. Vaccine protects against infection and disease, and vaccine coverage is 50% for each strategy 15](#_Toc212121925)

[Supplementary figure 11 Vaccine distribution process 36](#_Toc212121926)

[Supplementary figure 12 Vaccine doses by age group, week, and subnational state (target coverage 50%) 37](#_Toc212121927)

[Supplementary figure 13 State-level with- and without vaccination epidemic curves (disease-blocking efficacy) 38](#_Toc212121928)

[Supplementary figure 14 State-level with- and without vaccination epidemic curves (disease and infection blocking efficacy) 39](#_Toc212121929)

[Supplementary figure 15 Number needed to avert a single symptomatic, fatal case, and DALY by vaccination scenario, region, age group (Ixchiq) 40](#_Toc212121930)

[Supplementary figure 16 Number needed to avert a single symptomatic, fatal case, and DALY by vaccination scenario, region, age group (Vimkunya) 43](#_Toc212121931)

[Supplementary figure 17 One-way sensitivity results by vaccination strategy and region 46](#_Toc212121932)

[Supplementary figure 18 One-way sensitivity results by vaccination strategy at national level 47](#_Toc212121933)

[Supplementary figure 19Vaccine coverage and delay scenario analyses (deployment weeks varied from week1 to 52 by 1 week, and vaccine coverage varied from 10% to 100% by 10%) 48](#_Toc212121934)

**Tables**

[Supplementary table 1 Model predicted long-term average annual chikungunya symptomatic cases by age in Brazil 5](#_Toc212121935)

[Supplementary table 2 Model predicted vs. reported annual symptomatic cases from 2015-2024 5](#_Toc212121936)

[Supplementary table 3 Reported symptomatic cases, population, and group classification 9](#_Toc212121937)

[Supplementary table 4 Model parameters (disability-adjusted life years related parameters) 16](#_Toc212121938)

[Supplementary table 5 Posterior checks by state 21](#_Toc212121939)

[Supplementary table 6 NNVs at sub-national level (Ixchiq) 49](#_Toc212121940)

[Supplementary table 7 NNVs at sub-national level (Vimkunya) 63](#_Toc212121941)

Supplementary figure 1 Model diagram and equation

$$R_{a}$$

$$S_{a}$$

$$I_{a}$$

$$E_{a}$$

$$\gamma$$

$$\sigma$$

$$\lambda$$

$$v_{a}(t)$$

Symptomatic outcome

= (1 - $\mathrm{VE}_{\mathrm{block}}*coverage$) * $I_{a}$

$$V_{a}$$

Immunity acquisition

Vaccine campaign

Total supply

VE against disease ($\mathrm{VE}_{\mathrm{bl}ock}$)

VE against disease and infection ($\mathrm{VE}_{\inf}$)

$$\frac{dS_{a}}{dt}=-\beta\left( t \right)*\frac{I}{N} *S_{a}-v_{a}(t)*S_{a}$$

$\gamma$: recovery rate

$\lambda$*:* force of infection *(*$\beta\left( t \right) *\frac{I}{N} )$

$\sigma:$ latent to infectious rate

$\beta\left( t \right)$: transmission rate

$v_{a}(t)$: $\mathrm{VE}_{\inf}$x (vaccine coverage)

$\mathrm{ve}$: vaccine efficacy

$$\frac{dE_{a}}{dt}=\beta\left( t \right)*\frac{I}{N} *S_{a}- \sigma*E_{a}$$

$$\frac{dI_{a}}{dt}=\sigma*E_{a}-\gamma*I_{a}$$

$$\frac{dR_{a}}{dt}=\gamma*I_{a}$$

$$\frac{dV_{a}}{dt}=v_{a}(t)*S_{a}$$

We used age-structured dynamic transmission SEIRV model across 20 age groups (0 to > 80 years old; <1, 1-4, 5-9, 10-11, 12-17, 18-19, 20-24, 25-29, 30-34, 35-39, 40-44, 45-49, 50-54, 55-59, 60-65, 65-69, 70-75, 75-79, 80-84, 85+). We simulated transitions between susceptible $(S_{a})$, Exposed ${(E}_{a})$, infected $(I_{a})$, recovered $(R_{a})$ with added vaccinated $(V_{a})$ compartment. We assumed (i) disease blocking only efficacy, and (ii) disease and infection-blocking vaccine efficacy mechanisms.

Under disease blocking efficacy assumption, vaccine dose does not alter force of infection, and thus the total infections will remain the same. However, total symptomatic individuals will decrease by disease blocking efficacy and vaccine coverage. Under disease and infection blocking efficacy assumption, successfully vaccinated individuals acquire immunity after certain days post-vaccination (14-days for Ixchiq, 21-days for Vimkunya) and removed from susceptible compartment, thus changing the force of infection. Therefore, there were both direct impacts (on vaccinated) and indirect impacts (non-vaccinated) of vaccination. We assumed all-or-nothing vaccine, so successfully vaccinated individuals in the $V$ compartment will remain in the $V$ compartment, without moving into the $I$ compartment again. We assumed that transmission rate $(\beta_{t})$ is time-varying each week from week 1 to 52. We used literature-based information for the recovery rate $(\gamma)$. The reporting rate was calibrated by the difference between the long-term average annual total cumulative symptomatic cases (2015–2024) predicted from the chikungunya FOI map and the total symptomatic cases in 2022 from the counterfactual (without vaccination) scenario. [^1^](https://sciwheel.com/work/citation?ids=18332060&pre=&suf=&sa=0&dbf=0) Across all age groups, total aggregated symptomatic cases are predicted as 883,544 (95%UI: 684,889-1,079,146) (Table S1). Across 10 years, the average reported symptomatic cases are 222,468. Therefore, the mean reporting rate at the national level is 25% (95%UI: 21 – 32) and we used this as prior information using Beta distribution.

For calibration, weekly transmission rates ($\beta_{t}$) were treated as and initialised on [0,6] to regularise transmission rates to epidemiologically plausible values while allowing dispersion. Each parameter of interest for calibrations followed below distributions:

$$\beta_{t} \sim Lognormal \left( -1, 0.5 \right)$$

$$\rho_{s} \sim Beta (20, 60)$$

$$\gamma\sim normal (0.67, 0.02)$$

To validate the choice of prior distributions of transmission rates, we conducted prior-predictive simulation. We conducted 1,000 times of prior predictive simulations with the combinations of three parameters. From the 1,000 random draws, the infection curve exhibited realistic outbreak dynamics (timing, peak size, and duration), covering ranges of plausible infection peaks (max 10,000 cases as close to the maximum cases from the reported data).

To calibrate the recovery rate, we used evidence from literature that the duration of infection ranges from 7 to 10 days and converted this to weekly rate so that around 1-week time (= 7 to 10 days), the infectiousness resolves. Weekly calibrated transmission rates are provided in Figure S4.

Supplementary table 1 Model predicted long-term average annual chikungunya symptomatic cases by age in Brazil

The predicted symptomatic cases each show aggregated cases at the national level. Symptomatic case was estimated from global FOI prediction map which is predicted at 5x5 km [^1^](https://sciwheel.com/work/citation?ids=18332060&pre=&suf=&sa=0&dbf=0).

| **Age group** | **Total (95% median)** | **Total (95% lo)** | **Total (95% hi)** |
| --- | --- | --- | --- |
| [0,10) | 172,779 | 133,931 | 211,029 |
| [10,20) | 163,170 | 126,483 | 199,294 |
| [20,30) | 156,672 | 121,446 | 191,357 |
| [30,40) | 138,724 | 107,533 | 169,434 |
| [40,50) | 104,237 | 80,801 | 127,313 |
| [50,60) | 74,534 | 57,776 | 91,034 |
| [60,70) | 44,930 | 34,828 | 54,877 |
| [70,80) | 20,571 | 15,946 | 25,125 |
| [80,90) | 7,928 | 6,145 | 9,683 |

Supplementary table 2 Model predicted vs. reported annual symptomatic cases from 2015-2024

|  | reported symptomatic cases (surveillance) | predicted annual symptomatic cases (95% mid) | predicted annual symptomatic cases (95% lo) | predicted annual symptomatic cases (95% hi) |
| --- | --- | --- | --- | --- |
| 2015 | 16411 | 883544 | 684889 | 1079146 |
| 2016 | 558542 | 883544 | 684889 | 1079146 |
| 2017 | 195962 | 883544 | 684889 | 1079146 |
| 2018 | 87687 | 883544 | 684889 | 1079146 |
| 2019 | 178147 | 883544 | 684889 | 1079146 |
| 2020 | 98177 | 883544 | 684889 | 1079146 |
| 2021 | 132587 | 883544 | 684889 | 1079146 |
| 2022 | 265289 | 883544 | 684889 | 1079146 |
| 2023 | 266297 | 883544 | 684889 | 1079146 |
| 2024 | 425587 | 883544 | 684889 | 1079146 |

Supplementary figure 2 Reported chikungunya outbreak case data in Brazil by state (2022)





Supplementary figure 3 Total case and peak symptomatic cases (per million) distribution and state classification


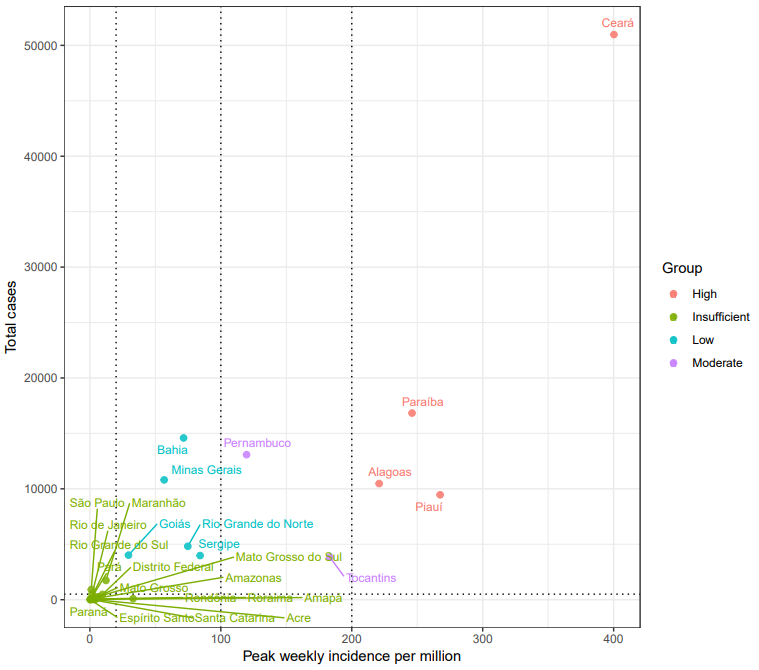


Supplementary figure 4 Total cumulative reported cases and per capita cases by state in Brazil (2022).


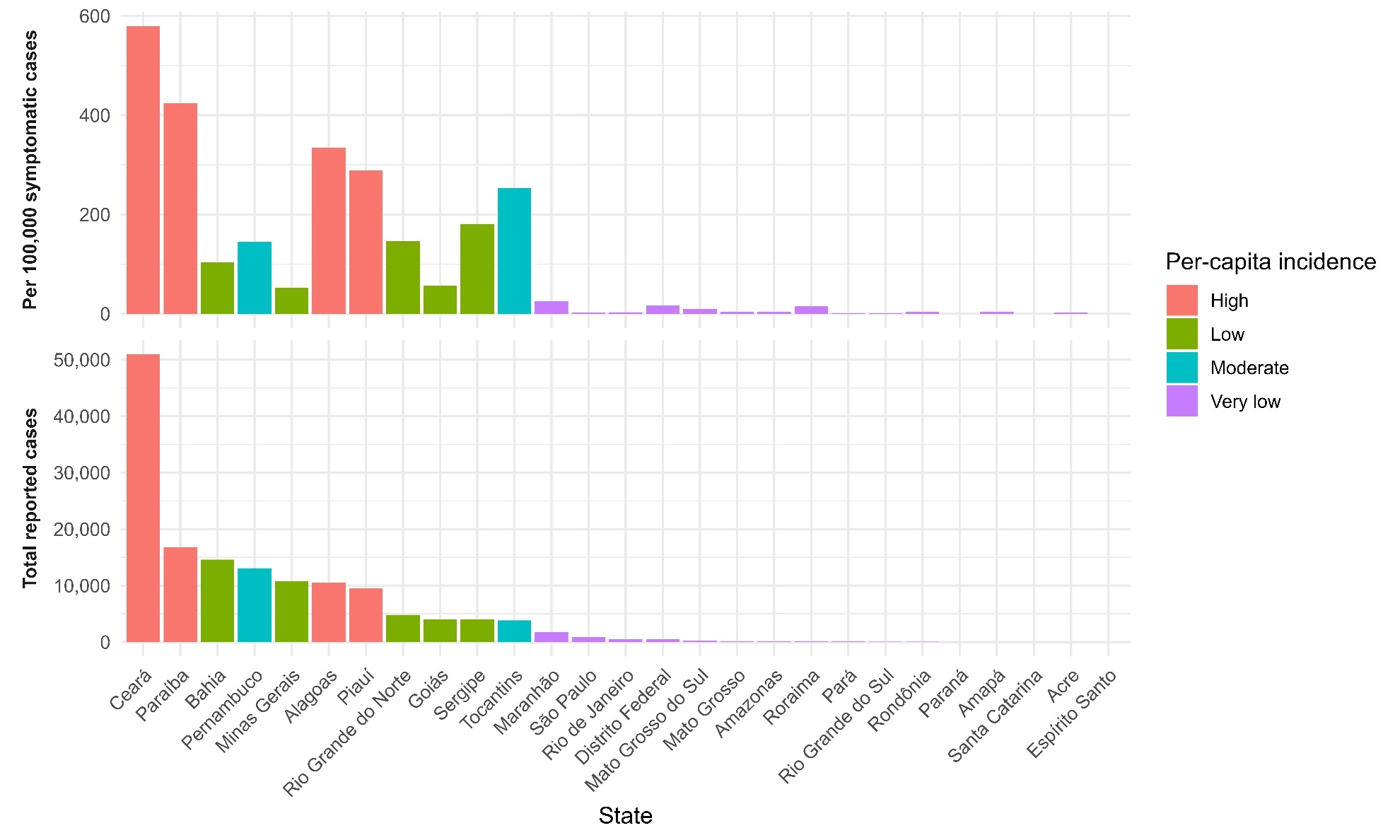


Supplementary table 3 Reported symptomatic cases, population, and group classification

We performed k-means clustering using peak incidence per million and total cases to examine the empirical clustering pattern of the states. The three clusters exhibited distinct median pack incidence of 73, 233, and 400 per million. These empirical cluster centroids support our choice of operational threshold for categorisation of <100 (low), 100-200 (moderate), >200 (high). We chose round number thresholds (100, 200) for interpretability while ensuring they are aligned with natural data structure.

|  | State | Peak cases | Total population | Peak cases per million | Total reported cases | Group |
| --- | --- | --- | --- | --- | --- | --- |
| 1 | Ceara | 3520 | 8794957 | 400 | 50975 | High |
| 2 | Minas Gerais | 1165 | 20539989 | 57 | 10806 | Low |
| 3 | Pernambuco | 1084 | 9058931 | 120 | 13080 | Moderate |
| 4 | Bahia | 1012 | 14141626 | 72 | 14589 | Low |
| 5 | Paraiba | 978 | 3974687 | 246 | 16834 | High |
| 6 | Piaui | 875 | 3271199 | 267 | 9462 | High |
| 7 | Alagoas | 691 | 3127683 | 221 | 10475 | High |
| 8 | Tocantins | 277 | 1511460 | 183 | 3835 | Moderate |
| 9 | Rio Grande do Norte | 247 | 3302729 | 75 | 4818 | Low |
| 10 | Goias | 208 | 7056495 | 29 | 4018 | Low |
| 11 | Sergipe | 186 | 2210004 | 84 | 3981 | Low |
| 12 | Maranhao | 83 | 6776699 | 12 | 1736 | Insufficient |
| 13 | Sao Paulo | 47 | 44411238 | 1 | 909 | Insufficient |
| 14 | Distrito Federal | 27 | 2817381 | 10 | 474 | Insufficient |
| 15 | Rio de Janeiro | 27 | 16055174 | 2 | 511 | Insufficient |
| 16 | Amazonas | 22 | 3941613 | 6 | 142 | Insufficient |
| 17 | Roraima | 21 | 636707 | 33 | 95 | Insufficient |
| 18 | Mato Grosso do Sul | 16 | 2757013 | 6 | 260 | Insufficient |
| 19 | Mato Grosso | 13 | 3658649 | 4 | 155 | Insufficient |
| 20 | Para | 7 | 8120131 | 1 | 87 | Insufficient |
| 21 | Rio Grande do Sul | 6 | 10882965 | 1 | 66 | Insufficient |
| 22 | Rondonia | 6 | 1581196 | 4 | 64 | Insufficient |
| 23 | Santa Catarina | 5 | 7610361 | 1 | 25 | Insufficient |
| 24 | Amapa | 3 | 733759 | 4 | 25 | Insufficient |
| 25 | Paraa | 3 | 11444380 | 0 | 28 | Insufficient |
| 26 | Acre | 2 | 830018 | 2 | 17 | Insufficient |
| 27 | Esperito Santo | 1 | 3833712 | 0 | 2 | Insufficient |

Supplementary figure 5 Model calibrated weekly transmission rates with 95% UI by sub-national state


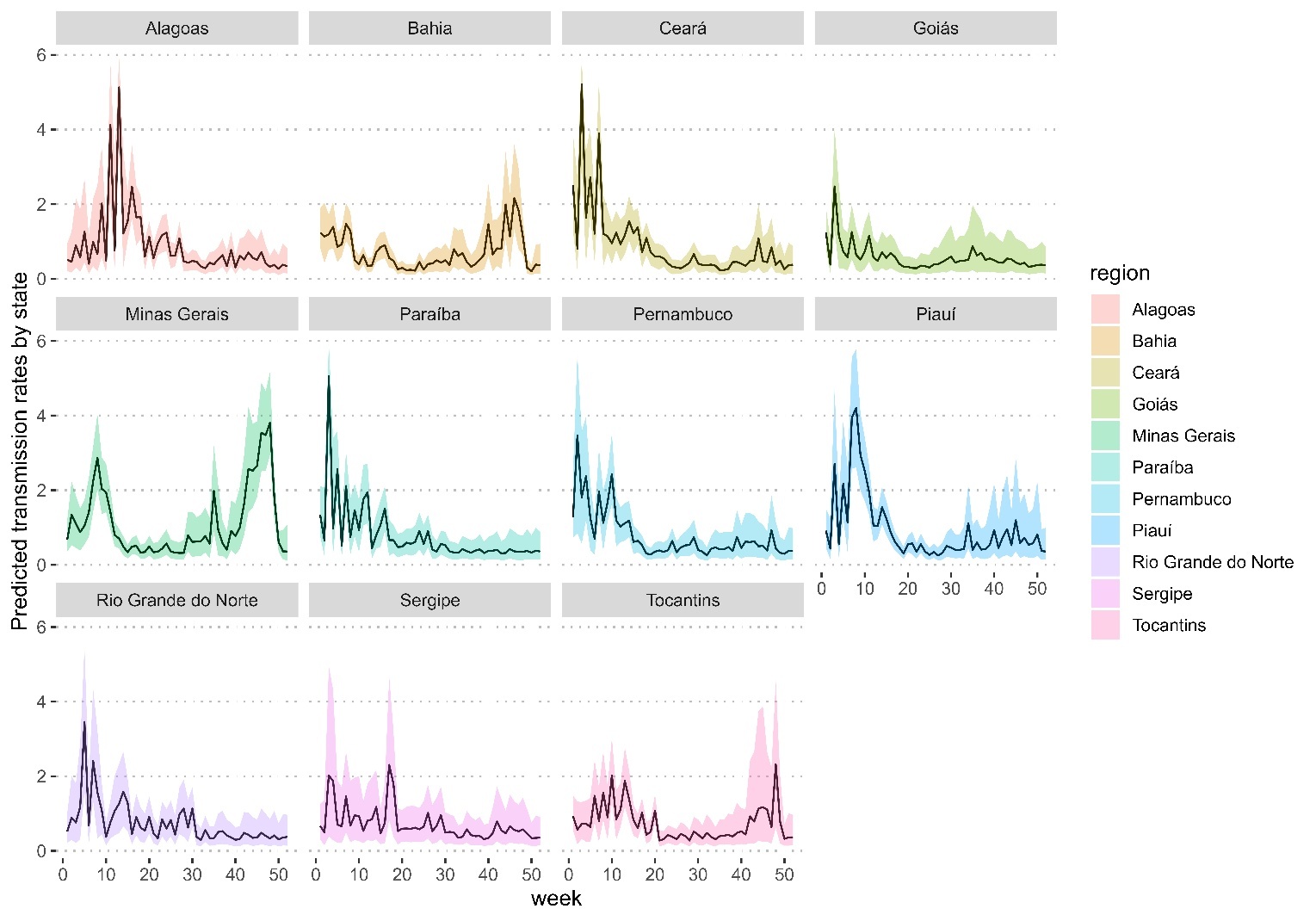


Supplementary figure 6 Predicted reporting rates by state (median and 95%UI)


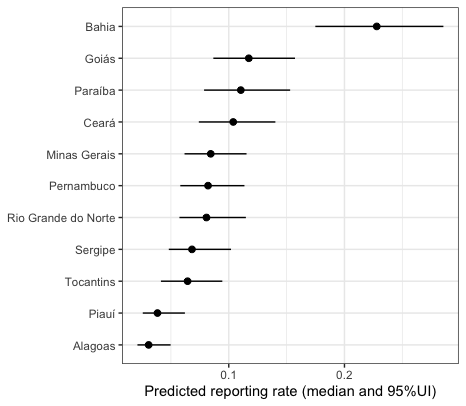


Supplementary figure 7 Model fitted epidemic curve with reported cumulative case data in Brazil (2022) by state

**
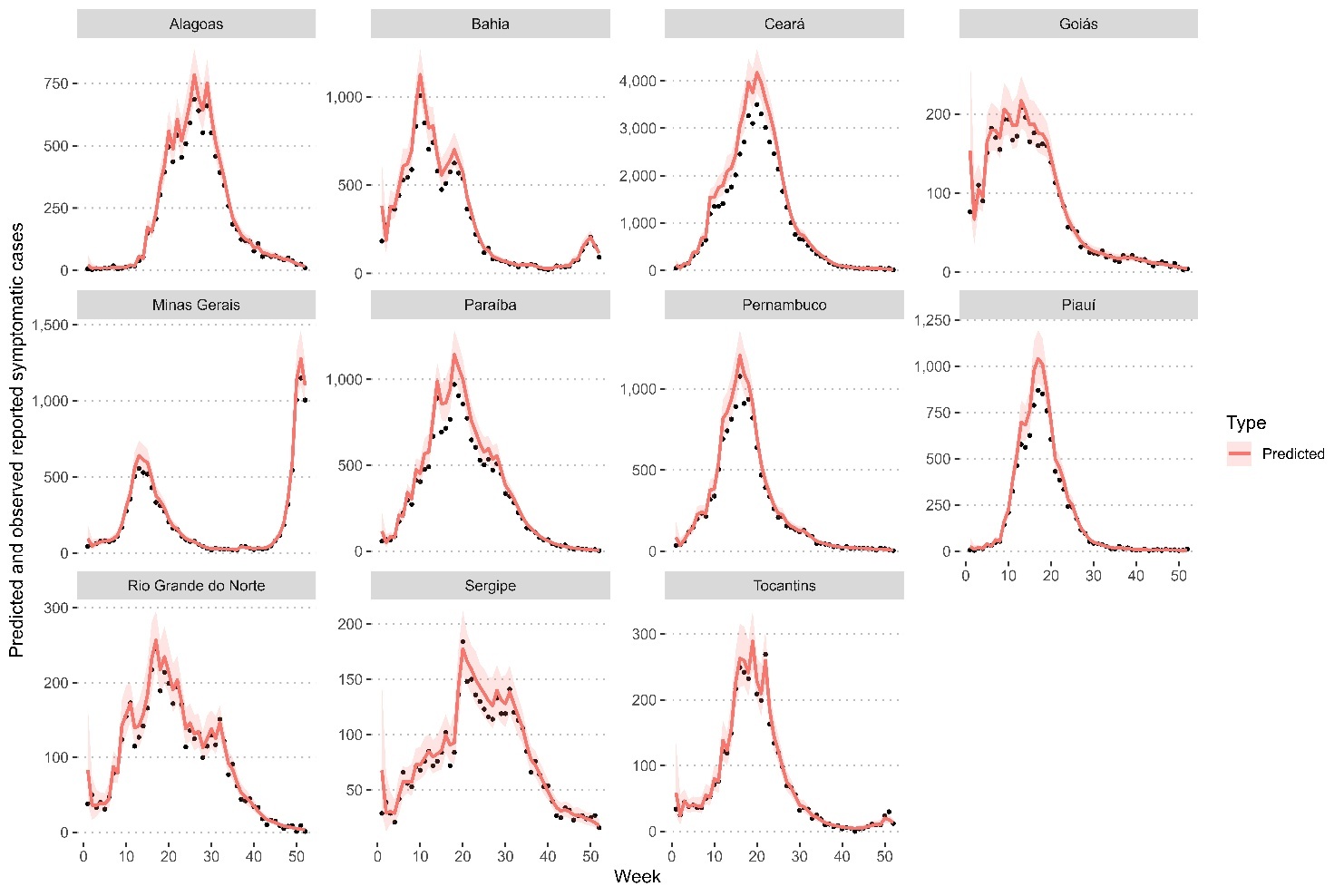
**

Supplementary figure 8 Age-specific pre-vaccination symptomatic cases per million population

**
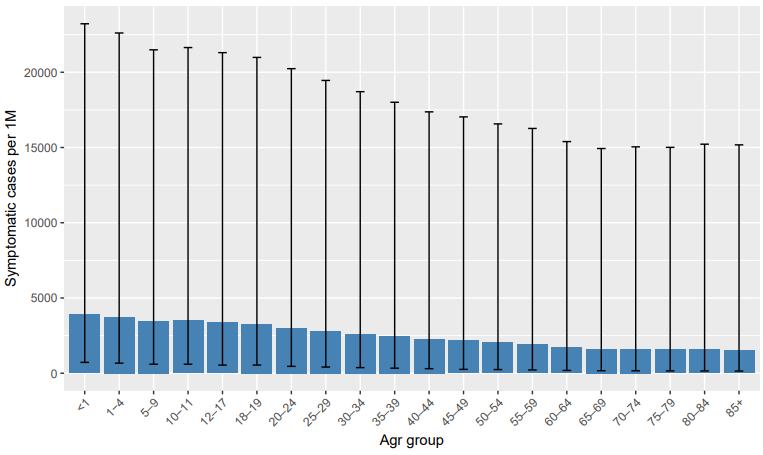
**

Supplementary figure 9 DALY reduction from each age-specific vaccination strategy by component (acute, sub-acute, chronic YLDs, and YLL)

DALY reduction by component when vaccine protection against both disease and infection and vaccine coverage is 50%. The first panel shows averted DALYs per million vaccinated population, and the second panel shows percent reduction in total DALYs for each vaccination strategy.


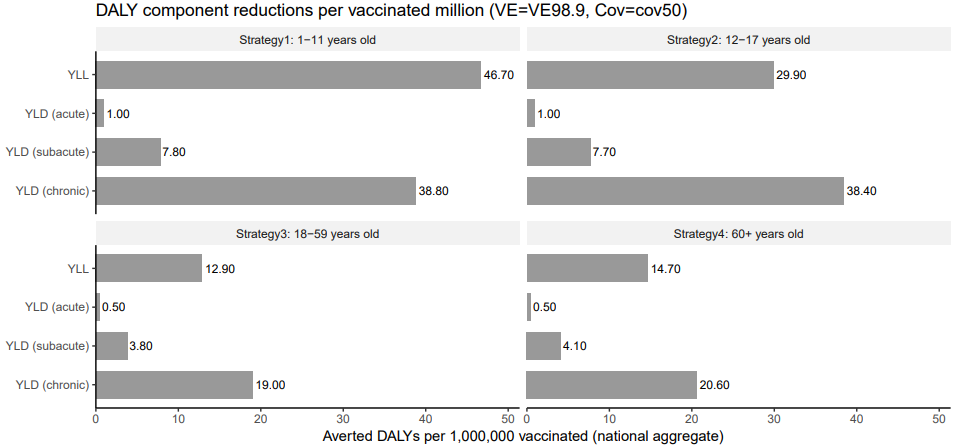


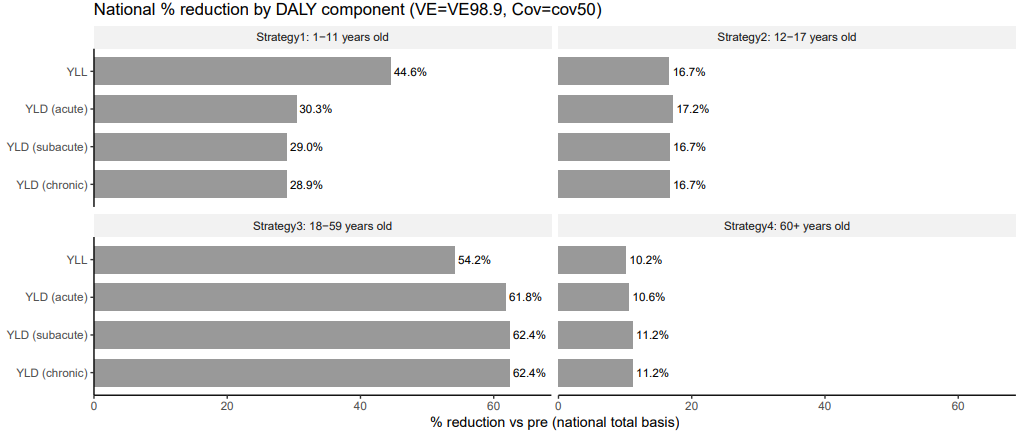


Supplementary figure 10 Direct and indirect effects of vaccination strategy at national and sub-national levels. Vaccine protects against infection and disease, and vaccine coverage is 50% for each strategy


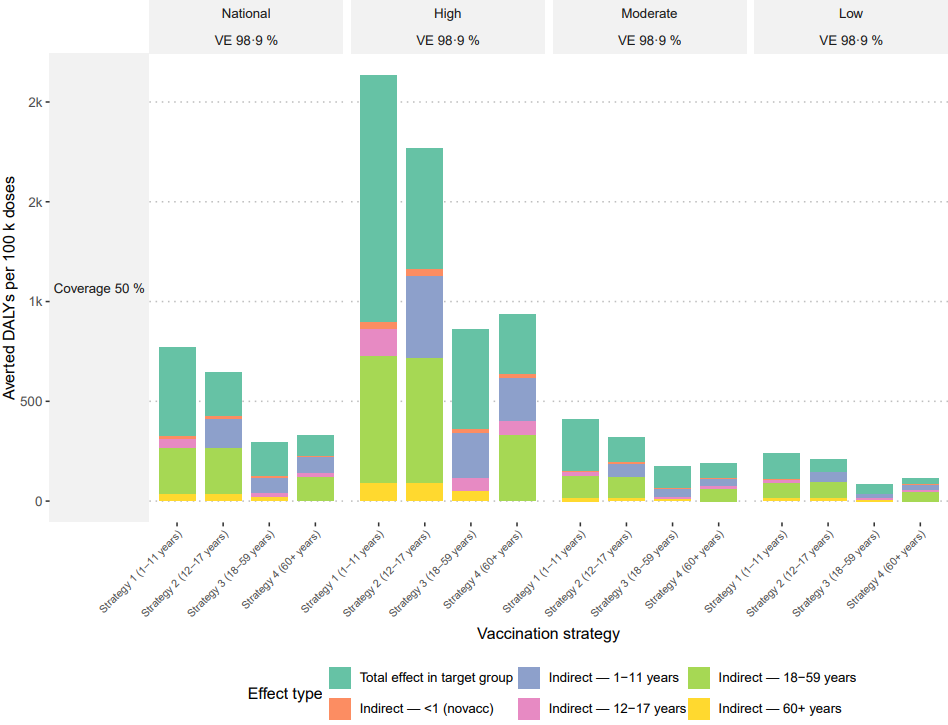


**Uncertainty analysis**

We propagated parameter uncertainty via Latin Hypercube Sampling (LHS). For each state, we generated 1,000 samples from the joint parameter space and ran the model for each draw, summarising the median and 95% uncertainty intervals (UIs) across runs. Parameters sampled included initial infections, transmission rate, reporting rate, recovery rate, latent period, long-term average FOI, vaccine efficacy, vaccine coverage, weekly delivery speed, time to acquisition of immunity, and DALY parameters. To support reproducibility, we have deposited the state-specific LHS matrices and code in our GitHub repository and fixed random seeds for the reported analyses (CHIK_VIM/01_Script/lhs_samples_sir.R).

Supplementary table 4 Model parameters (disability-adjusted life years related parameters)

| Parameter | Median (95% UI) | Uncertainty distribution | Reference |
| --- | --- | --- | --- |
| **Disease progression probabilities** | | | |
| Probability of symptomatic cases among infections | Overall:  52.4% (40.6, 64.1) | Beta  [α=35.84, β=32.56] | [^2^](https://sciwheel.com/work/citation?ids=6089155&pre=&suf=&sa=0&dbf=0) |
|  | Asia:  49% (48.4, 69.4) | Beta  [α=49.1, β=34.1] |  |
|  | Africa:  51.7% (39.7, 63.6) | Beta  [α=34.2, β=31.9] |  |
|  | Americas:  52.9% (41.2, 64.5) | Beta  [α=36.8, β=32.7] |  |
| Probability of hospitalisation among symptomatic cases | 4% (3, 5) | Beta  [α=59.0, β=1415.2] | [^3^](https://sciwheel.com/work/citation?ids=16033691&pre=&suf=&sa=0&dbf=0) |
| Probability of death among hospitalised cases | Age [0, 10):  1.8% (1.2, 2.5) | Beta  [α=26.7, β=1455.7] | [^4^](https://sciwheel.com/work/citation?ids=16987000&pre=&suf=&sa=0&dbf=0) |
|  | Age [10, 20):  1.5% (1.3, 1.8) | Beta  [α=133.0, β=8443.6] |  |
|  | Age [20, 30):  1.8% (1.6, 2.0) | Beta  [α=407.9, β=22412.5] |  |
|  | Age [30, 40):  1.9% (1.7, 2.1) | Beta  [α=493.2, β=25471.5] |  |
|  | Age [40, 50):  2.3% (2.2, 2.5) | Beta  [α=789.5, β=33197.9] |  |
|  | Age [50, 60):  2.7% (2.5, 2.9) | Beta  [α=944.9, β=34239.3] |  |
|  | Age [60, 70):  4.5% (4.3, 4.8) | Beta  [α=1805.2, β=37879.3] |  |
|  | Age [70, 80):  10.8% (10.5, 11.1) | Beta  [α=4466.9, β=36999.1] |  |
|  | Age [80, 90):  18.8% (18.2, 19.3) | Beta  [α=3801.2, β=16439.2] |  |
| Probability of death among non-hospitalised cases | Age [0, 10):  0.03% (0.014, 0.05) | Beta  [α=9.7, β=30950.3] | [^4^](https://sciwheel.com/work/citation?ids=16987000&pre=&suf=&sa=0&dbf=0) |
|  | Age [10, 20):  0.01% (0.004, 0.02) | Beta  [α=6.1, β=54677.4] |  |
|  | Age [20, 30):  0.003% (0.0006, 0.009) | Beta  [α=2.6, β=70752.9] |  |
|  | Age [30, 40):  0.01% (0.005, 0.02) | Beta  [α=9.7, β=93187.2] |  |
|  | Age [40, 50):  0.004% (0.001, 0.01) | Beta  [α=3.4, β=76516.7] |  |
|  | Age [50, 60):  0.02% (0.011, 0.03) | Beta  [α=16.2, β=79639.98] |  |
|  | Age [60, 70):  0.04% (0.02, .0.05) | Beta  [α=19.9, β=54639.2] |  |
|  | Age [70, 80):  0.1% (0.06, 0.1) | Beta  [α=27.5, β=28387.1] |  |
|  | Age [80, 90):  0.4% (0.29, 0.53) | Beta  [α=39.98, β=9914.8] |  |
| Probability of recovery within 14 days after onset of symptoms | age < 40 years:  41.9% (38.7, 45.0) | Beta  [α=393.5, β=547.0] | [^5^](https://sciwheel.com/work/citation?ids=12383358&pre=&suf=&sa=0&dbf=0) |
|  | age > 40 years:  34.3% (31.6, 37.1) | Beta  [α=388.3, β=742.5] |  |
| Probability of recovery within 90 days after acute period | age < 40 years:  29.5% (29.1, 29.8) | Beta  [α=17875.9, β=42754.6] | [^5^](https://sciwheel.com/work/citation?ids=12383358&pre=&suf=&sa=0&dbf=0) |
|  | age > 40 years:  27.8% (26.9, 28.8) | Beta  [α=2487.1, β=6450.8] |  |
| Probability of recovery within 6 months after sub-acute period | age < 40 years:  21.7% (21.2, 22.2) | Beta  [α=799.3, β=3307.0] | [^5^](https://sciwheel.com/work/citation?ids=12383358&pre=&suf=&sa=0&dbf=0) |
|  | age > 40 years:  19.5% (18.3, 20.7) | Beta  [α=5998.9, β=21654.9] |  |
| Probability of recovery within 12 months after 6 months of chronicity | age < 40 years:  8.5% (6.8, 10.4) | Beta  [α=77.6, β=836.7] | [^5^](https://sciwheel.com/work/citation?ids=12383358&pre=&suf=&sa=0&dbf=0) |
|  | age > 40 years:  13.2% (11.5, 15.0) | Beta  [α=184.7, β=1216.1] |  |
| Probability of recovery within 30 months after 12 months of chronicity | age < 40 years:  0.7% (0.3, 1.3) | Beta  [α=7.6, β=1075.0] | [^5^](https://sciwheel.com/work/citation?ids=12383358&pre=&suf=&sa=0&dbf=0) |
|  | age > 40 years:  2.9% (1.7, 4.6) | Beta  [α=15.8, β=516.3] |  |

|  | Parameter | Median (95%UI) | Uncertainty distribution  (parameters) | Description | Ref |
| --- | --- | --- | --- | --- | --- |
| **Disability weights and duration of illness** | | | | | |
|  | Disability weight for mild and moderate chikungunya | 0.051 (95%UI: 0.032, 0.074) | Beta  [α= 32.45,  β= 399.2] | We took a disability weight for acute myocarditis from IHME as a proxy for acute moderate/mild chikungunya: has a fever and aches, and feels weak, which causes some difficulty with daily activities. | [^6^](https://sciwheel.com/work/citation?ids=13449189&pre=&suf=&sa=0&dbf=0) |
|  | Disability weight for severe chikungunya | 0.133 (95%UI: 0.088, 0.19) | Beta  [α=22.52, β=146.80] | We took a disability weight for severe malaria described in IHME as a proxy for severe (hospitalized) chikungunya: has a high fever and pain, and feels very weak, which causes great difficulty with daily activities | [^6^](https://sciwheel.com/work/citation?ids=13449189&pre=&suf=&sa=0&dbf=0) |
|  | Disability weight for chronic chikungunya | 0.317 (95%UI: 0.117, 0.581) | Beta  [α=4.58, β=9.87] | We took a disability weight from rheumatoid arthritis from OECD Public health explorer as a proxy for chronic chikungunya. Moderate DW was taken for median estimate and mild and severe DWs for upper and lower limit values. | [^7^](https://sciwheel.com/work/citation?ids=16665325&pre=&suf=&sa=0&dbf=0) |
|  | Duration of illness for mild and moderate chikungunya (years) | 0.016 (95%UI: 0.005, 0.058) | Lognormal  (meanlog =  -4.1, SDlog= 0.599) | Acutely infected symptomatic chikungunya patients (without further chronic or severe symptoms) resolve within 7-10 days from multiple sources; CDC Yellow Book 2024, WHO Chikungunya fact sheet, PAHO) | [^8,9^](https://sciwheel.com/work/citation?ids=13353318,15507432&pre=&pre=&suf=&suf=&sa=0,0&dbf=0&dbf=0) |
|  | Duration of illness for severe chikungunya (years) | 0.019 (95%UI: 0.008, 0.04) | Lognormal  (meanlog =  -3.73, SDlog= 0.39) | We took multiple studies that report chikungunya hospitalization and reported duration of stay until discharge and estimated median, and 95%UI ranges. We used R metfor package to obtain pooled estimate of median and 95%UI of duration of hospital stays |  |
|  | Duration of illness for chronic chikungunya (years) | 0.53 (95%UI: 0.46, 0.64) | Lognormal  (meanlog =  -0.6309,  SDlog = 0.0852) | We used arthralgia resolution rate following chikungunya virus infection for chronic chikungunya duration. A review of cohort studies show that expected median time to arthralgia resolution is 6.39months ranging between 5.48 to 7.66 months. | [^5^](https://sciwheel.com/work/citation?ids=12383358&pre=&suf=&sa=0&dbf=0) |
| **Age-related parameters** | | | | | |
|  | Remaining life-years | Age [0, 10):  70.9  Age [10, 20):  61.5  Age [20, 30):  52.2  Age [30, 40):  42.9  Age [40, 50):  33.9  Age [50, 60):  25.3  Age [60, 70):  17.6  Age [70, 80):  11  Age [80, 90):  6.1 | Age [0, 10):  (meanlog =  4.27,  Sdlog=  0.05)  Age [10, 20):  (meanlog=  4.12,  Sdlog=  0.05)  Age [20, 30):  (meanlog=  3.96,  Sdlog=  0.05)  Age [30, 40):  (meanlog=  3.76,  Sdlog=  0.05)  Age [40, 50):  (meanlog=  3.52,  Sdlog=  0.05)  Age [50, 60):  (meanlog=  3.23,  Sdlog=  0.05)  Age [60, 70):  (meanlog=  2.87,  Sdlog=  0.05)  Age [70, 80):  (meanlog=  2.39,  Sdlog=  0.05)  Age [80, 90):  (meanlog=  1.81,  Sdlog=  0.05) | We used World Population Prospects from UN (UNWPP) for age-specific life-expectancies for each age group. We took the median age within each age group, and computed remaining life years by deducting median age at death from life-expectancy | [^10^](https://sciwheel.com/work/citation?ids=8436701&pre=&suf=&sa=0&dbf=0) |

Supplementary table5 Posterior checks by state

ESS: Effective sample size, and median, lower_95, uppoer_95 each refers to median, 2.5% and 97.5% from 95% uncertainty distributions

| **parameter** | **median** | **lower_95** | **upper_95** | **ESS** | **Rhat** | **region** |
| --- | --- | --- | --- | --- | --- | --- |
| beta_week1 | 0.514 | 0.207 | 0.943 | 370.887 | 0.999 | Alagoas |
| beta_week2 | 0.455 | 0.165 | 1.266 | 384.243 | 1.002 | Alagoas |
| beta_week3 | 0.892 | 0.300 | 2.158 | 387.107 | 1.001 | Alagoas |
| beta_week4 | 0.575 | 0.186 | 1.855 | 495.529 | 1.001 | Alagoas |
| beta_week5 | 1.258 | 0.441 | 2.701 | 373.413 | 0.998 | Alagoas |
| beta_week6 | 0.401 | 0.133 | 1.156 | 416.559 | 1.007 | Alagoas |
| beta_week7 | 0.985 | 0.340 | 2.150 | 354.387 | 0.999 | Alagoas |
| beta_week8 | 0.659 | 0.198 | 2.505 | 301.967 | 0.999 | Alagoas |
| beta_week9 | 2.016 | 0.585 | 3.548 | 349.245 | 0.998 | Alagoas |
| beta_week10 | 0.489 | 0.168 | 1.444 | 211.996 | 1.018 | Alagoas |
| beta_week11 | 4.120 | 2.094 | 5.799 | 246.471 | 1.000 | Alagoas |
| beta_week12 | 0.762 | 0.206 | 3.212 | 336.764 | 0.999 | Alagoas |
| beta_week13 | 5.130 | 3.586 | 5.957 | 267.106 | 1.009 | Alagoas |
| beta_week14 | 1.212 | 0.306 | 3.413 | 347.387 | 1.001 | Alagoas |
| beta_week15 | 1.533 | 1.028 | 2.343 | 279.317 | 0.999 | Alagoas |
| beta_week16 | 2.470 | 1.616 | 3.594 | 400.460 | 1.000 | Alagoas |
| beta_week17 | 1.648 | 0.898 | 2.504 | 359.364 | 0.998 | Alagoas |
| beta_week18 | 1.653 | 1.073 | 2.239 | 328.982 | 0.998 | Alagoas |
| beta_week19 | 0.596 | 0.254 | 1.046 | 261.298 | 1.000 | Alagoas |
| beta_week20 | 1.124 | 0.764 | 1.541 | 430.937 | 1.000 | Alagoas |
| beta_week21 | 0.548 | 0.241 | 0.957 | 441.981 | 0.998 | Alagoas |
| beta_week22 | 0.955 | 0.637 | 1.350 | 414.776 | 0.999 | Alagoas |
| beta_week23 | 1.170 | 0.704 | 1.693 | 326.151 | 0.998 | Alagoas |
| beta_week24 | 1.237 | 0.740 | 1.746 | 417.394 | 1.005 | Alagoas |
| beta_week25 | 0.613 | 0.276 | 0.989 | 342.235 | 1.026 | Alagoas |
| beta_week26 | 0.616 | 0.335 | 0.941 | 440.605 | 0.999 | Alagoas |
| beta_week27 | 1.081 | 0.745 | 1.523 | 400.947 | 1.004 | Alagoas |
| beta_week28 | 0.473 | 0.203 | 0.805 | 327.582 | 1.001 | Alagoas |
| beta_week29 | 0.429 | 0.212 | 0.671 | 346.056 | 0.998 | Alagoas |
| beta_week30 | 0.477 | 0.230 | 0.784 | 406.396 | 0.999 | Alagoas |
| beta_week31 | 0.460 | 0.229 | 0.740 | 318.424 | 1.000 | Alagoas |
| beta_week32 | 0.329 | 0.167 | 0.519 | 379.450 | 1.007 | Alagoas |
| beta_week33 | 0.283 | 0.122 | 0.487 | 387.546 | 1.001 | Alagoas |
| beta_week34 | 0.432 | 0.187 | 0.703 | 373.019 | 0.998 | Alagoas |
| beta_week35 | 0.386 | 0.170 | 0.743 | 407.462 | 1.000 | Alagoas |
| beta_week36 | 0.525 | 0.240 | 0.847 | 340.911 | 1.003 | Alagoas |
| beta_week37 | 0.638 | 0.320 | 1.077 | 419.307 | 0.998 | Alagoas |
| beta_week38 | 0.339 | 0.139 | 0.736 | 322.060 | 1.000 | Alagoas |
| beta_week39 | 0.772 | 0.344 | 1.248 | 346.862 | 0.998 | Alagoas |
| beta_week40 | 0.305 | 0.125 | 0.607 | 291.019 | 0.998 | Alagoas |
| beta_week41 | 0.615 | 0.302 | 1.075 | 386.267 | 0.998 | Alagoas |
| beta_week42 | 0.516 | 0.209 | 1.123 | 420.354 | 0.999 | Alagoas |
| beta_week43 | 0.705 | 0.287 | 1.332 | 284.522 | 1.002 | Alagoas |
| beta_week44 | 0.564 | 0.215 | 1.171 | 463.956 | 0.999 | Alagoas |
| beta_week45 | 0.509 | 0.217 | 1.039 | 338.207 | 1.008 | Alagoas |
| beta_week46 | 0.715 | 0.278 | 1.319 | 412.356 | 1.017 | Alagoas |
| beta_week47 | 0.414 | 0.161 | 0.905 | 384.907 | 0.998 | Alagoas |
| beta_week48 | 0.320 | 0.143 | 0.615 | 373.204 | 0.999 | Alagoas |
| beta_week49 | 0.375 | 0.163 | 0.812 | 377.628 | 0.999 | Alagoas |
| beta_week50 | 0.268 | 0.099 | 0.629 | 323.726 | 0.998 | Alagoas |
| beta_week51 | 0.376 | 0.155 | 0.950 | 306.639 | 1.000 | Alagoas |
| beta_week52 | 0.338 | 0.145 | 0.814 | 381.031 | 1.000 | Alagoas |
| beta_week1 | 1.238 | 0.835 | 1.885 | 352.921 | 1.002 | Bahia |
| beta_week2 | 1.132 | 0.422 | 2.041 | 281.516 | 1.001 | Bahia |
| beta_week3 | 1.193 | 0.777 | 1.714 | 486.756 | 1.000 | Bahia |
| beta_week4 | 1.392 | 0.812 | 2.062 | 365.993 | 0.999 | Bahia |
| beta_week5 | 0.860 | 0.443 | 1.251 | 418.262 | 0.998 | Bahia |
| beta_week6 | 0.922 | 0.597 | 1.375 | 443.217 | 0.998 | Bahia |
| beta_week7 | 1.468 | 1.011 | 2.028 | 487.016 | 0.998 | Bahia |
| beta_week8 | 1.289 | 0.826 | 1.794 | 477.925 | 0.999 | Bahia |
| beta_week9 | 0.506 | 0.257 | 0.809 | 552.705 | 0.999 | Bahia |
| beta_week10 | 0.393 | 0.205 | 0.627 | 557.148 | 1.007 | Bahia |
| beta_week11 | 0.633 | 0.380 | 0.870 | 532.533 | 1.005 | Bahia |
| beta_week12 | 0.348 | 0.161 | 0.552 | 423.065 | 0.999 | Bahia |
| beta_week13 | 0.356 | 0.198 | 0.581 | 502.088 | 1.000 | Bahia |
| beta_week14 | 0.712 | 0.444 | 1.060 | 500.598 | 0.998 | Bahia |
| beta_week15 | 0.846 | 0.513 | 1.249 | 451.034 | 0.999 | Bahia |
| beta_week16 | 0.900 | 0.551 | 1.259 | 518.524 | 0.998 | Bahia |
| beta_week17 | 0.556 | 0.293 | 0.901 | 425.609 | 1.001 | Bahia |
| beta_week18 | 0.484 | 0.278 | 0.706 | 454.336 | 1.000 | Bahia |
| beta_week19 | 0.248 | 0.119 | 0.421 | 426.387 | 0.998 | Bahia |
| beta_week20 | 0.296 | 0.161 | 0.482 | 435.183 | 0.999 | Bahia |
| beta_week21 | 0.230 | 0.104 | 0.386 | 417.011 | 0.998 | Bahia |
| beta_week22 | 0.241 | 0.111 | 0.401 | 371.508 | 1.003 | Bahia |
| beta_week23 | 0.217 | 0.111 | 0.392 | 529.150 | 0.998 | Bahia |
| beta_week24 | 0.448 | 0.206 | 0.701 | 449.885 | 1.000 | Bahia |
| beta_week25 | 0.245 | 0.112 | 0.468 | 410.881 | 0.998 | Bahia |
| beta_week26 | 0.396 | 0.178 | 0.681 | 524.175 | 0.998 | Bahia |
| beta_week27 | 0.409 | 0.184 | 0.778 | 500.779 | 0.998 | Bahia |
| beta_week28 | 0.494 | 0.205 | 0.861 | 475.993 | 0.998 | Bahia |
| beta_week29 | 0.432 | 0.178 | 0.804 | 530.687 | 1.000 | Bahia |
| beta_week30 | 0.497 | 0.203 | 0.949 | 522.058 | 0.998 | Bahia |
| beta_week31 | 0.369 | 0.163 | 0.763 | 242.396 | 0.999 | Bahia |
| beta_week32 | 0.792 | 0.324 | 1.387 | 467.064 | 0.998 | Bahia |
| beta_week33 | 0.611 | 0.234 | 1.297 | 521.289 | 0.998 | Bahia |
| beta_week34 | 0.692 | 0.285 | 1.336 | 506.039 | 0.999 | Bahia |
| beta_week35 | 0.454 | 0.192 | 0.870 | 507.994 | 0.999 | Bahia |
| beta_week36 | 0.323 | 0.136 | 0.645 | 488.832 | 1.003 | Bahia |
| beta_week37 | 0.391 | 0.156 | 0.779 | 463.305 | 1.000 | Bahia |
| beta_week38 | 0.515 | 0.200 | 1.132 | 319.664 | 1.001 | Bahia |
| beta_week39 | 0.645 | 0.251 | 1.557 | 451.442 | 0.998 | Bahia |
| beta_week40 | 1.460 | 0.603 | 2.532 | 504.917 | 0.998 | Bahia |
| beta_week41 | 0.650 | 0.204 | 1.574 | 628.350 | 0.998 | Bahia |
| beta_week42 | 0.814 | 0.343 | 1.594 | 371.224 | 1.004 | Bahia |
| beta_week43 | 0.807 | 0.269 | 1.777 | 489.032 | 0.998 | Bahia |
| beta_week44 | 1.985 | 1.036 | 3.406 | 443.560 | 0.999 | Bahia |
| beta_week45 | 1.139 | 0.314 | 2.300 | 399.979 | 1.001 | Bahia |
| beta_week46 | 2.163 | 1.327 | 3.597 | 448.749 | 1.007 | Bahia |
| beta_week47 | 1.837 | 0.795 | 2.976 | 455.805 | 0.998 | Bahia |
| beta_week48 | 1.127 | 0.642 | 1.629 | 430.357 | 0.999 | Bahia |
| beta_week49 | 0.292 | 0.139 | 0.547 | 501.968 | 1.002 | Bahia |
| beta_week50 | 0.202 | 0.102 | 0.384 | 482.001 | 1.010 | Bahia |
| beta_week51 | 0.383 | 0.145 | 0.910 | 466.132 | 0.998 | Bahia |
| beta_week52 | 0.354 | 0.142 | 0.936 | 489.791 | 0.999 | Bahia |
| beta_week1 | 2.516 | 1.281 | 3.886 | 76.807 | 1.007 | Ceará |
| beta_week2 | 0.806 | 0.207 | 2.735 | 66.657 | 0.996 | Ceará |
| beta_week3 | 5.213 | 3.021 | 5.898 | 69.572 | 0.996 | Ceará |
| beta_week4 | 1.638 | 0.406 | 3.421 | 69.476 | 0.996 | Ceará |
| beta_week5 | 2.727 | 1.733 | 4.050 | 90.453 | 0.996 | Ceará |
| beta_week6 | 1.208 | 0.405 | 2.191 | 121.222 | 1.003 | Ceará |
| beta_week7 | 3.896 | 2.588 | 5.353 | 100.275 | 1.005 | Ceará |
| beta_week8 | 1.205 | 0.334 | 2.248 | 109.312 | 1.004 | Ceará |
| beta_week9 | 1.140 | 0.712 | 1.874 | 126.728 | 1.026 | Ceará |
| beta_week10 | 0.953 | 0.396 | 1.502 | 88.688 | 1.019 | Ceará |
| beta_week11 | 1.235 | 0.776 | 1.817 | 149.707 | 1.003 | Ceará |
| beta_week12 | 0.929 | 0.462 | 1.587 | 181.741 | 1.015 | Ceará |
| beta_week13 | 1.168 | 0.653 | 1.713 | 162.266 | 1.007 | Ceará |
| beta_week14 | 1.550 | 1.022 | 2.229 | 168.889 | 1.018 | Ceará |
| beta_week15 | 1.221 | 0.671 | 1.818 | 154.922 | 0.995 | Ceará |
| beta_week16 | 1.377 | 0.938 | 1.896 | 174.335 | 0.995 | Ceará |
| beta_week17 | 0.710 | 0.297 | 1.170 | 201.715 | 0.995 | Ceará |
| beta_week18 | 1.094 | 0.635 | 1.520 | 191.770 | 0.998 | Ceará |
| beta_week19 | 0.714 | 0.358 | 1.146 | 194.287 | 1.018 | Ceará |
| beta_week20 | 0.596 | 0.292 | 0.950 | 179.301 | 0.999 | Ceará |
| beta_week21 | 0.605 | 0.239 | 0.932 | 179.435 | 0.996 | Ceará |
| beta_week22 | 0.549 | 0.333 | 0.874 | 213.052 | 1.000 | Ceará |
| beta_week23 | 0.414 | 0.184 | 0.687 | 159.613 | 1.006 | Ceará |
| beta_week24 | 0.322 | 0.124 | 0.517 | 129.071 | 1.001 | Ceará |
| beta_week25 | 0.303 | 0.126 | 0.515 | 154.444 | 0.996 | Ceará |
| beta_week26 | 0.280 | 0.140 | 0.485 | 127.421 | 0.995 | Ceará |
| beta_week27 | 0.348 | 0.178 | 0.606 | 173.546 | 1.022 | Ceará |
| beta_week28 | 0.433 | 0.202 | 0.765 | 189.849 | 1.002 | Ceará |
| beta_week29 | 0.664 | 0.324 | 0.987 | 138.137 | 0.995 | Ceará |
| beta_week30 | 0.406 | 0.160 | 0.742 | 194.295 | 0.996 | Ceará |
| beta_week31 | 0.371 | 0.176 | 0.610 | 207.125 | 0.996 | Ceará |
| beta_week32 | 0.364 | 0.208 | 0.637 | 202.338 | 1.012 | Ceará |
| beta_week33 | 0.380 | 0.168 | 0.637 | 177.335 | 0.996 | Ceará |
| beta_week34 | 0.351 | 0.172 | 0.637 | 151.790 | 1.000 | Ceará |
| beta_week35 | 0.235 | 0.102 | 0.420 | 82.582 | 1.045 | Ceará |
| beta_week36 | 0.234 | 0.114 | 0.435 | 197.720 | 0.995 | Ceará |
| beta_week37 | 0.264 | 0.128 | 0.491 | 208.919 | 1.002 | Ceará |
| beta_week38 | 0.446 | 0.189 | 0.843 | 189.995 | 0.995 | Ceará |
| beta_week39 | 0.452 | 0.184 | 0.898 | 220.618 | 0.996 | Ceará |
| beta_week40 | 0.402 | 0.166 | 0.775 | 158.133 | 0.998 | Ceará |
| beta_week41 | 0.349 | 0.151 | 0.793 | 229.256 | 0.997 | Ceará |
| beta_week42 | 0.476 | 0.231 | 0.941 | 184.278 | 1.005 | Ceará |
| beta_week43 | 0.490 | 0.185 | 1.227 | 148.836 | 1.001 | Ceará |
| beta_week44 | 1.081 | 0.294 | 2.014 | 169.640 | 1.007 | Ceará |
| beta_week45 | 0.475 | 0.163 | 1.123 | 237.966 | 1.002 | Ceará |
| beta_week46 | 0.445 | 0.176 | 1.234 | 145.934 | 1.004 | Ceará |
| beta_week47 | 0.840 | 0.337 | 1.630 | 140.741 | 1.021 | Ceará |
| beta_week48 | 0.370 | 0.152 | 0.812 | 92.342 | 1.048 | Ceará |
| beta_week49 | 0.488 | 0.153 | 1.002 | 202.562 | 0.995 | Ceará |
| beta_week50 | 0.253 | 0.107 | 0.621 | 150.953 | 1.009 | Ceará |
| beta_week51 | 0.366 | 0.141 | 0.967 | 162.041 | 0.998 | Ceará |
| beta_week52 | 0.378 | 0.131 | 0.875 | 128.223 | 0.999 | Ceará |
| beta_week1 | 0.675 | 0.356 | 1.201 | 441.217 | 0.998 | Minas Gerais |
| beta_week2 | 1.341 | 0.527 | 2.247 | 332.783 | 1.003 | Minas Gerais |
| beta_week3 | 1.103 | 0.455 | 1.857 | 402.426 | 1.010 | Minas Gerais |
| beta_week4 | 0.874 | 0.384 | 1.516 | 441.681 | 0.998 | Minas Gerais |
| beta_week5 | 1.047 | 0.546 | 1.741 | 312.028 | 0.999 | Minas Gerais |
| beta_week6 | 1.404 | 0.750 | 2.326 | 387.064 | 0.998 | Minas Gerais |
| beta_week7 | 2.243 | 1.427 | 3.166 | 477.973 | 0.998 | Minas Gerais |
| beta_week8 | 2.869 | 2.024 | 4.051 | 441.193 | 1.003 | Minas Gerais |
| beta_week9 | 2.037 | 1.359 | 2.876 | 396.983 | 1.000 | Minas Gerais |
| beta_week10 | 1.942 | 1.411 | 2.713 | 395.226 | 1.004 | Minas Gerais |
| beta_week11 | 1.416 | 0.944 | 2.030 | 372.564 | 0.999 | Minas Gerais |
| beta_week12 | 0.796 | 0.493 | 1.151 | 443.114 | 1.000 | Minas Gerais |
| beta_week13 | 0.708 | 0.441 | 0.972 | 460.410 | 0.999 | Minas Gerais |
| beta_week14 | 0.483 | 0.252 | 0.765 | 425.528 | 1.002 | Minas Gerais |
| beta_week15 | 0.340 | 0.173 | 0.533 | 453.374 | 0.999 | Minas Gerais |
| beta_week16 | 0.491 | 0.276 | 0.726 | 442.101 | 1.001 | Minas Gerais |
| beta_week17 | 0.510 | 0.270 | 0.789 | 477.234 | 1.001 | Minas Gerais |
| beta_week18 | 0.329 | 0.175 | 0.540 | 369.242 | 1.004 | Minas Gerais |
| beta_week19 | 0.344 | 0.163 | 0.577 | 319.703 | 1.002 | Minas Gerais |
| beta_week20 | 0.496 | 0.247 | 0.781 | 479.426 | 1.000 | Minas Gerais |
| beta_week21 | 0.338 | 0.151 | 0.585 | 420.159 | 1.001 | Minas Gerais |
| beta_week22 | 0.353 | 0.158 | 0.609 | 449.585 | 0.998 | Minas Gerais |
| beta_week23 | 0.435 | 0.207 | 0.778 | 465.593 | 1.000 | Minas Gerais |
| beta_week24 | 0.571 | 0.270 | 0.948 | 548.472 | 1.000 | Minas Gerais |
| beta_week25 | 0.357 | 0.166 | 0.679 | 457.400 | 0.998 | Minas Gerais |
| beta_week26 | 0.327 | 0.154 | 0.629 | 434.080 | 0.998 | Minas Gerais |
| beta_week27 | 0.317 | 0.144 | 0.604 | 503.433 | 0.998 | Minas Gerais |
| beta_week28 | 0.320 | 0.149 | 0.664 | 428.355 | 0.998 | Minas Gerais |
| beta_week29 | 0.791 | 0.358 | 1.413 | 389.316 | 1.004 | Minas Gerais |
| beta_week30 | 0.615 | 0.205 | 1.413 | 420.810 | 1.000 | Minas Gerais |
| beta_week31 | 0.623 | 0.237 | 1.281 | 428.994 | 0.998 | Minas Gerais |
| beta_week32 | 0.635 | 0.240 | 1.345 | 289.889 | 0.999 | Minas Gerais |
| beta_week33 | 0.774 | 0.331 | 1.537 | 325.634 | 0.998 | Minas Gerais |
| beta_week34 | 0.568 | 0.179 | 1.268 | 434.015 | 0.998 | Minas Gerais |
| beta_week35 | 1.978 | 0.881 | 3.193 | 437.840 | 0.999 | Minas Gerais |
| beta_week36 | 0.934 | 0.247 | 2.127 | 444.643 | 0.998 | Minas Gerais |
| beta_week37 | 0.578 | 0.241 | 1.182 | 372.465 | 1.001 | Minas Gerais |
| beta_week38 | 0.405 | 0.153 | 0.884 | 464.632 | 0.998 | Minas Gerais |
| beta_week39 | 0.909 | 0.397 | 1.717 | 331.763 | 1.003 | Minas Gerais |
| beta_week40 | 0.772 | 0.275 | 1.651 | 463.177 | 1.001 | Minas Gerais |
| beta_week41 | 1.001 | 0.421 | 2.085 | 377.550 | 0.998 | Minas Gerais |
| beta_week42 | 1.636 | 0.796 | 2.940 | 509.538 | 1.005 | Minas Gerais |
| beta_week43 | 2.568 | 1.414 | 4.246 | 454.082 | 0.998 | Minas Gerais |
| beta_week44 | 2.522 | 1.534 | 3.776 | 336.949 | 0.998 | Minas Gerais |
| beta_week45 | 2.653 | 1.811 | 3.843 | 519.231 | 1.001 | Minas Gerais |
| beta_week46 | 3.541 | 2.509 | 4.892 | 286.533 | 1.001 | Minas Gerais |
| beta_week47 | 3.478 | 2.514 | 4.652 | 439.390 | 1.001 | Minas Gerais |
| beta_week48 | 3.805 | 2.946 | 5.163 | 253.922 | 0.998 | Minas Gerais |
| beta_week49 | 1.793 | 1.187 | 2.456 | 330.755 | 0.999 | Minas Gerais |
| beta_week50 | 0.616 | 0.357 | 0.899 | 523.316 | 0.998 | Minas Gerais |
| beta_week51 | 0.363 | 0.144 | 0.940 | 428.257 | 0.998 | Minas Gerais |
| beta_week52 | 0.358 | 0.116 | 1.069 | 343.912 | 0.999 | Minas Gerais |
| beta_week1 | 1.272 | 0.720 | 2.518 | 307.998 | 1.005 | Pernambuco |
| beta_week2 | 3.473 | 0.846 | 5.651 | 317.082 | 0.998 | Pernambuco |
| beta_week3 | 1.809 | 0.569 | 3.578 | 332.571 | 1.000 | Pernambuco |
| beta_week4 | 2.373 | 1.206 | 3.978 | 342.831 | 0.998 | Pernambuco |
| beta_week5 | 1.256 | 0.386 | 2.193 | 456.134 | 1.004 | Pernambuco |
| beta_week6 | 0.703 | 0.277 | 1.257 | 364.873 | 1.005 | Pernambuco |
| beta_week7 | 1.963 | 1.334 | 3.122 | 273.907 | 1.003 | Pernambuco |
| beta_week8 | 1.124 | 0.371 | 1.989 | 394.416 | 1.002 | Pernambuco |
| beta_week9 | 1.676 | 1.157 | 2.471 | 411.421 | 1.001 | Pernambuco |
| beta_week10 | 2.435 | 1.635 | 3.526 | 338.992 | 1.001 | Pernambuco |
| beta_week11 | 1.185 | 0.488 | 1.812 | 446.692 | 1.005 | Pernambuco |
| beta_week12 | 1.027 | 0.605 | 1.539 | 298.289 | 1.005 | Pernambuco |
| beta_week13 | 1.116 | 0.649 | 1.660 | 359.428 | 0.998 | Pernambuco |
| beta_week14 | 1.187 | 0.757 | 1.709 | 468.599 | 0.999 | Pernambuco |
| beta_week15 | 0.614 | 0.288 | 1.050 | 393.693 | 1.000 | Pernambuco |
| beta_week16 | 0.644 | 0.337 | 0.932 | 407.698 | 1.000 | Pernambuco |
| beta_week17 | 0.527 | 0.254 | 0.785 | 370.179 | 0.998 | Pernambuco |
| beta_week18 | 0.302 | 0.146 | 0.520 | 505.911 | 0.998 | Pernambuco |
| beta_week19 | 0.278 | 0.137 | 0.459 | 487.258 | 0.998 | Pernambuco |
| beta_week20 | 0.355 | 0.175 | 0.594 | 470.548 | 0.999 | Pernambuco |
| beta_week21 | 0.385 | 0.177 | 0.640 | 534.892 | 1.007 | Pernambuco |
| beta_week22 | 0.345 | 0.167 | 0.600 | 383.493 | 0.999 | Pernambuco |
| beta_week23 | 0.394 | 0.188 | 0.684 | 475.917 | 1.005 | Pernambuco |
| beta_week24 | 0.640 | 0.337 | 0.991 | 339.392 | 1.001 | Pernambuco |
| beta_week25 | 0.339 | 0.141 | 0.628 | 451.121 | 1.001 | Pernambuco |
| beta_week26 | 0.467 | 0.240 | 0.784 | 503.791 | 1.001 | Pernambuco |
| beta_week27 | 0.522 | 0.234 | 0.885 | 509.241 | 0.998 | Pernambuco |
| beta_week28 | 0.533 | 0.226 | 1.006 | 537.921 | 1.000 | Pernambuco |
| beta_week29 | 0.739 | 0.317 | 1.216 | 477.308 | 0.998 | Pernambuco |
| beta_week30 | 0.400 | 0.167 | 0.714 | 621.715 | 1.000 | Pernambuco |
| beta_week31 | 0.353 | 0.141 | 0.651 | 418.425 | 1.013 | Pernambuco |
| beta_week32 | 0.250 | 0.107 | 0.467 | 381.527 | 0.998 | Pernambuco |
| beta_week33 | 0.427 | 0.185 | 0.767 | 402.591 | 0.999 | Pernambuco |
| beta_week34 | 0.467 | 0.192 | 0.946 | 535.397 | 0.999 | Pernambuco |
| beta_week35 | 0.417 | 0.173 | 0.847 | 483.955 | 0.998 | Pernambuco |
| beta_week36 | 0.420 | 0.161 | 0.881 | 463.631 | 1.001 | Pernambuco |
| beta_week37 | 0.476 | 0.185 | 0.882 | 470.633 | 0.998 | Pernambuco |
| beta_week38 | 0.370 | 0.143 | 0.889 | 481.058 | 0.998 | Pernambuco |
| beta_week39 | 0.750 | 0.252 | 1.466 | 363.505 | 1.000 | Pernambuco |
| beta_week40 | 0.452 | 0.167 | 1.056 | 454.634 | 0.998 | Pernambuco |
| beta_week41 | 0.624 | 0.217 | 1.398 | 449.577 | 1.000 | Pernambuco |
| beta_week42 | 0.608 | 0.202 | 1.389 | 489.141 | 0.999 | Pernambuco |
| beta_week43 | 0.635 | 0.221 | 1.330 | 519.914 | 0.999 | Pernambuco |
| beta_week44 | 0.494 | 0.185 | 1.163 | 473.594 | 0.999 | Pernambuco |
| beta_week45 | 0.514 | 0.191 | 1.075 | 402.817 | 0.999 | Pernambuco |
| beta_week46 | 0.383 | 0.129 | 0.892 | 471.487 | 0.998 | Pernambuco |
| beta_week47 | 0.927 | 0.302 | 1.868 | 388.472 | 0.999 | Pernambuco |
| beta_week48 | 0.445 | 0.154 | 1.155 | 526.791 | 0.999 | Pernambuco |
| beta_week49 | 0.322 | 0.127 | 0.745 | 470.321 | 0.999 | Pernambuco |
| beta_week50 | 0.296 | 0.117 | 0.665 | 522.910 | 1.001 | Pernambuco |
| beta_week51 | 0.378 | 0.151 | 0.993 | 468.831 | 0.999 | Pernambuco |
| beta_week52 | 0.379 | 0.144 | 0.984 | 480.167 | 0.999 | Pernambuco |
| beta_week1 | 0.918 | 0.454 | 1.508 | 428.157 | 0.999 | Piauí |
| beta_week2 | 0.435 | 0.150 | 1.249 | 432.461 | 0.998 | Piauí |
| beta_week3 | 2.708 | 1.516 | 4.719 | 384.779 | 0.998 | Piauí |
| beta_week4 | 0.551 | 0.175 | 1.764 | 425.383 | 1.000 | Piauí |
| beta_week5 | 2.174 | 1.251 | 3.891 | 432.118 | 1.002 | Piauí |
| beta_week6 | 1.139 | 0.325 | 2.602 | 458.322 | 0.999 | Piauí |
| beta_week7 | 3.941 | 2.579 | 5.569 | 290.811 | 1.002 | Piauí |
| beta_week8 | 4.208 | 2.609 | 5.781 | 430.732 | 1.001 | Piauí |
| beta_week9 | 2.936 | 1.977 | 4.059 | 480.446 | 0.998 | Piauí |
| beta_week10 | 2.510 | 1.707 | 3.413 | 448.966 | 0.999 | Piauí |
| beta_week11 | 1.953 | 1.352 | 2.663 | 500.348 | 1.003 | Piauí |
| beta_week12 | 1.056 | 0.616 | 1.549 | 537.740 | 1.000 | Piauí |
| beta_week13 | 1.042 | 0.702 | 1.480 | 447.161 | 0.998 | Piauí |
| beta_week14 | 1.543 | 1.094 | 2.086 | 527.584 | 0.999 | Piauí |
| beta_week15 | 1.238 | 0.792 | 1.720 | 399.925 | 1.002 | Piauí |
| beta_week16 | 0.873 | 0.570 | 1.254 | 335.471 | 1.003 | Piauí |
| beta_week17 | 0.612 | 0.379 | 0.886 | 462.877 | 1.005 | Piauí |
| beta_week18 | 0.465 | 0.278 | 0.687 | 425.607 | 0.999 | Piauí |
| beta_week19 | 0.301 | 0.153 | 0.487 | 417.814 | 0.999 | Piauí |
| beta_week20 | 0.551 | 0.346 | 0.768 | 463.442 | 0.998 | Piauí |
| beta_week21 | 0.574 | 0.281 | 0.884 | 495.065 | 1.002 | Piauí |
| beta_week22 | 0.352 | 0.166 | 0.577 | 424.582 | 0.999 | Piauí |
| beta_week23 | 0.552 | 0.320 | 0.841 | 440.995 | 0.999 | Piauí |
| beta_week24 | 0.354 | 0.168 | 0.602 | 491.739 | 0.999 | Piauí |
| beta_week25 | 0.269 | 0.129 | 0.459 | 381.793 | 0.998 | Piauí |
| beta_week26 | 0.343 | 0.188 | 0.570 | 306.026 | 0.998 | Piauí |
| beta_week27 | 0.251 | 0.115 | 0.468 | 388.613 | 0.999 | Piauí |
| beta_week28 | 0.320 | 0.137 | 0.593 | 459.425 | 1.000 | Piauí |
| beta_week29 | 0.506 | 0.222 | 0.862 | 485.290 | 1.010 | Piauí |
| beta_week30 | 0.458 | 0.192 | 0.987 | 414.571 | 1.003 | Piauí |
| beta_week31 | 0.400 | 0.174 | 0.813 | 413.226 | 1.001 | Piauí |
| beta_week32 | 0.399 | 0.181 | 0.810 | 418.970 | 0.999 | Piauí |
| beta_week33 | 0.428 | 0.169 | 1.030 | 436.299 | 1.009 | Piauí |
| beta_week34 | 1.121 | 0.438 | 2.159 | 520.146 | 1.004 | Piauí |
| beta_week35 | 0.402 | 0.151 | 1.058 | 442.100 | 0.998 | Piauí |
| beta_week36 | 0.598 | 0.217 | 1.226 | 332.740 | 1.002 | Piauí |
| beta_week37 | 0.408 | 0.157 | 0.962 | 352.088 | 1.000 | Piauí |
| beta_week38 | 0.426 | 0.156 | 1.050 | 373.726 | 0.999 | Piauí |
| beta_week39 | 0.540 | 0.188 | 1.552 | 353.069 | 0.999 | Piauí |
| beta_week40 | 0.935 | 0.310 | 2.188 | 487.123 | 1.002 | Piauí |
| beta_week41 | 0.453 | 0.158 | 1.196 | 479.456 | 1.005 | Piauí |
| beta_week42 | 0.745 | 0.222 | 1.973 | 467.329 | 0.998 | Piauí |
| beta_week43 | 0.957 | 0.263 | 2.423 | 410.241 | 1.000 | Piauí |
| beta_week44 | 0.526 | 0.193 | 1.430 | 495.136 | 0.998 | Piauí |
| beta_week45 | 1.194 | 0.360 | 2.877 | 507.722 | 0.998 | Piauí |
| beta_week46 | 0.583 | 0.184 | 1.652 | 439.910 | 0.999 | Piauí |
| beta_week47 | 0.726 | 0.232 | 1.909 | 330.045 | 0.998 | Piauí |
| beta_week48 | 0.534 | 0.193 | 1.374 | 391.865 | 0.998 | Piauí |
| beta_week49 | 0.581 | 0.183 | 1.511 | 487.875 | 0.999 | Piauí |
| beta_week50 | 0.818 | 0.236 | 2.242 | 418.722 | 1.000 | Piauí |
| beta_week51 | 0.371 | 0.134 | 0.952 | 366.587 | 0.999 | Piauí |
| beta_week52 | 0.357 | 0.144 | 0.998 | 396.349 | 0.999 | Piauí |
| beta_week1 | 1.247 | 0.804 | 1.930 | 420.672 | 0.999 | Goiás |
| beta_week2 | 0.381 | 0.145 | 1.046 | 384.681 | 0.999 | Goiás |
| beta_week3 | 2.474 | 1.319 | 4.032 | 255.862 | 1.000 | Goiás |
| beta_week4 | 1.153 | 0.266 | 2.512 | 408.568 | 0.999 | Goiás |
| beta_week5 | 0.730 | 0.273 | 1.342 | 495.886 | 1.000 | Goiás |
| beta_week6 | 0.572 | 0.216 | 1.244 | 453.684 | 0.999 | Goiás |
| beta_week7 | 1.253 | 0.525 | 1.976 | 360.466 | 1.004 | Goiás |
| beta_week8 | 0.640 | 0.236 | 1.358 | 500.797 | 0.999 | Goiás |
| beta_week9 | 0.517 | 0.176 | 1.001 | 369.027 | 1.003 | Goiás |
| beta_week10 | 0.732 | 0.264 | 1.498 | 346.187 | 1.001 | Goiás |
| beta_week11 | 1.150 | 0.460 | 1.786 | 391.363 | 0.998 | Goiás |
| beta_week12 | 0.570 | 0.217 | 1.096 | 491.014 | 1.001 | Goiás |
| beta_week13 | 0.481 | 0.194 | 0.951 | 482.299 | 0.998 | Goiás |
| beta_week14 | 0.707 | 0.289 | 1.273 | 410.637 | 0.998 | Goiás |
| beta_week15 | 0.560 | 0.218 | 1.094 | 373.997 | 1.004 | Goiás |
| beta_week16 | 0.696 | 0.285 | 1.201 | 367.946 | 0.998 | Goiás |
| beta_week17 | 0.588 | 0.223 | 1.037 | 546.205 | 0.998 | Goiás |
| beta_week18 | 0.399 | 0.167 | 0.757 | 336.263 | 1.001 | Goiás |
| beta_week19 | 0.324 | 0.150 | 0.593 | 373.539 | 0.998 | Goiás |
| beta_week20 | 0.319 | 0.137 | 0.603 | 362.925 | 0.998 | Goiás |
| beta_week21 | 0.289 | 0.126 | 0.572 | 391.623 | 0.999 | Goiás |
| beta_week22 | 0.284 | 0.120 | 0.519 | 373.931 | 0.998 | Goiás |
| beta_week23 | 0.352 | 0.154 | 0.691 | 458.485 | 1.006 | Goiás |
| beta_week24 | 0.338 | 0.153 | 0.657 | 379.949 | 0.999 | Goiás |
| beta_week25 | 0.302 | 0.128 | 0.651 | 403.330 | 0.999 | Goiás |
| beta_week26 | 0.386 | 0.161 | 0.848 | 405.890 | 0.998 | Goiás |
| beta_week27 | 0.390 | 0.158 | 0.835 | 325.262 | 1.000 | Goiás |
| beta_week28 | 0.451 | 0.179 | 1.048 | 356.509 | 0.998 | Goiás |
| beta_week29 | 0.521 | 0.167 | 1.258 | 195.836 | 1.005 | Goiás |
| beta_week30 | 0.597 | 0.229 | 1.439 | 322.842 | 0.998 | Goiás |
| beta_week31 | 0.430 | 0.192 | 0.999 | 370.441 | 1.000 | Goiás |
| beta_week32 | 0.477 | 0.172 | 1.144 | 414.656 | 0.999 | Goiás |
| beta_week33 | 0.485 | 0.181 | 1.142 | 316.186 | 0.999 | Goiás |
| beta_week34 | 0.511 | 0.178 | 1.364 | 432.724 | 0.999 | Goiás |
| beta_week35 | 0.870 | 0.233 | 1.987 | 387.845 | 0.999 | Goiás |
| beta_week36 | 0.622 | 0.165 | 1.754 | 377.846 | 0.998 | Goiás |
| beta_week37 | 0.696 | 0.192 | 1.573 | 323.157 | 0.998 | Goiás |
| beta_week38 | 0.503 | 0.189 | 1.105 | 381.587 | 0.998 | Goiás |
| beta_week39 | 0.547 | 0.171 | 1.270 | 326.891 | 0.998 | Goiás |
| beta_week40 | 0.494 | 0.177 | 1.199 | 413.030 | 1.001 | Goiás |
| beta_week41 | 0.435 | 0.160 | 1.065 | 364.235 | 1.000 | Goiás |
| beta_week42 | 0.427 | 0.155 | 0.980 | 294.178 | 1.003 | Goiás |
| beta_week43 | 0.542 | 0.172 | 1.313 | 401.227 | 0.998 | Goiás |
| beta_week44 | 0.522 | 0.211 | 1.284 | 321.283 | 1.001 | Goiás |
| beta_week45 | 0.436 | 0.162 | 1.085 | 429.583 | 1.001 | Goiás |
| beta_week46 | 0.391 | 0.153 | 1.022 | 417.944 | 0.998 | Goiás |
| beta_week47 | 0.427 | 0.155 | 0.961 | 420.592 | 0.999 | Goiás |
| beta_week48 | 0.323 | 0.121 | 0.796 | 367.644 | 0.998 | Goiás |
| beta_week49 | 0.337 | 0.128 | 0.812 | 243.401 | 1.001 | Goiás |
| beta_week50 | 0.371 | 0.148 | 0.868 | 297.449 | 0.998 | Goiás |
| beta_week51 | 0.377 | 0.154 | 0.990 | 369.961 | 1.000 | Goiás |
| beta_week52 | 0.362 | 0.143 | 0.858 | 432.813 | 0.998 | Goiás |
| beta_week1 | 0.518 | 0.215 | 1.002 | 328.798 | 1.008 | Rio Grande do Norte |
| beta_week2 | 0.892 | 0.298 | 2.018 | 437.707 | 0.998 | Rio Grande do Norte |
| beta_week3 | 0.760 | 0.207 | 1.799 | 394.661 | 0.998 | Rio Grande do Norte |
| beta_week4 | 1.179 | 0.313 | 2.891 | 344.787 | 1.000 | Rio Grande do Norte |
| beta_week5 | 3.458 | 1.595 | 5.477 | 389.484 | 1.011 | Rio Grande do Norte |
| beta_week6 | 0.679 | 0.194 | 1.982 | 360.117 | 1.016 | Rio Grande do Norte |
| beta_week7 | 2.415 | 1.335 | 4.347 | 213.593 | 1.002 | Rio Grande do Norte |
| beta_week8 | 1.565 | 0.330 | 3.233 | 376.195 | 0.998 | Rio Grande do Norte |
| beta_week9 | 1.089 | 0.450 | 1.821 | 353.348 | 1.003 | Rio Grande do Norte |
| beta_week10 | 0.373 | 0.147 | 0.767 | 444.999 | 0.999 | Rio Grande do Norte |
| beta_week11 | 0.763 | 0.333 | 1.344 | 422.510 | 0.999 | Rio Grande do Norte |
| beta_week12 | 1.071 | 0.419 | 2.016 | 267.823 | 1.000 | Rio Grande do Norte |
| beta_week13 | 1.262 | 0.472 | 2.321 | 413.347 | 0.999 | Rio Grande do Norte |
| beta_week14 | 1.587 | 0.657 | 2.660 | 376.072 | 1.001 | Rio Grande do Norte |
| beta_week15 | 1.289 | 0.529 | 1.949 | 340.603 | 1.002 | Rio Grande do Norte |
| beta_week16 | 0.454 | 0.179 | 0.927 | 446.875 | 0.998 | Rio Grande do Norte |
| beta_week17 | 0.908 | 0.412 | 1.527 | 482.217 | 1.003 | Rio Grande do Norte |
| beta_week18 | 0.624 | 0.235 | 1.168 | 470.307 | 1.002 | Rio Grande do Norte |
| beta_week19 | 0.542 | 0.227 | 1.003 | 421.753 | 0.999 | Rio Grande do Norte |
| beta_week20 | 0.914 | 0.461 | 1.456 | 437.202 | 0.998 | Rio Grande do Norte |
| beta_week21 | 0.474 | 0.204 | 0.917 | 452.494 | 1.001 | Rio Grande do Norte |
| beta_week22 | 0.336 | 0.142 | 0.685 | 494.350 | 1.000 | Rio Grande do Norte |
| beta_week23 | 0.848 | 0.389 | 1.315 | 470.724 | 1.004 | Rio Grande do Norte |
| beta_week24 | 0.598 | 0.230 | 1.125 | 458.771 | 1.001 | Rio Grande do Norte |
| beta_week25 | 0.828 | 0.386 | 1.350 | 461.567 | 1.004 | Rio Grande do Norte |
| beta_week26 | 0.438 | 0.167 | 0.963 | 480.536 | 0.999 | Rio Grande do Norte |
| beta_week27 | 0.976 | 0.450 | 1.687 | 438.298 | 0.998 | Rio Grande do Norte |
| beta_week28 | 1.137 | 0.378 | 1.914 | 388.727 | 1.002 | Rio Grande do Norte |
| beta_week29 | 0.627 | 0.244 | 1.379 | 448.929 | 0.998 | Rio Grande do Norte |
| beta_week30 | 1.138 | 0.626 | 1.702 | 464.557 | 0.998 | Rio Grande do Norte |
| beta_week31 | 0.371 | 0.151 | 0.757 | 465.266 | 0.998 | Rio Grande do Norte |
| beta_week32 | 0.293 | 0.122 | 0.615 | 484.938 | 1.001 | Rio Grande do Norte |
| beta_week33 | 0.561 | 0.234 | 0.942 | 421.957 | 1.001 | Rio Grande do Norte |
| beta_week34 | 0.344 | 0.139 | 0.660 | 459.001 | 1.001 | Rio Grande do Norte |
| beta_week35 | 0.342 | 0.143 | 0.655 | 393.712 | 1.000 | Rio Grande do Norte |
| beta_week36 | 0.506 | 0.182 | 0.999 | 253.922 | 1.000 | Rio Grande do Norte |
| beta_week37 | 0.530 | 0.185 | 1.079 | 463.670 | 0.999 | Rio Grande do Norte |
| beta_week38 | 0.413 | 0.172 | 0.832 | 553.203 | 1.000 | Rio Grande do Norte |
| beta_week39 | 0.363 | 0.147 | 0.756 | 307.146 | 1.003 | Rio Grande do Norte |
| beta_week40 | 0.295 | 0.119 | 0.622 | 508.676 | 0.998 | Rio Grande do Norte |
| beta_week41 | 0.320 | 0.124 | 0.670 | 529.066 | 0.998 | Rio Grande do Norte |
| beta_week42 | 0.483 | 0.179 | 1.044 | 473.340 | 0.999 | Rio Grande do Norte |
| beta_week43 | 0.433 | 0.170 | 1.023 | 467.326 | 0.998 | Rio Grande do Norte |
| beta_week44 | 0.349 | 0.130 | 0.909 | 498.954 | 1.000 | Rio Grande do Norte |
| beta_week45 | 0.360 | 0.148 | 0.838 | 492.875 | 0.998 | Rio Grande do Norte |
| beta_week46 | 0.484 | 0.173 | 1.136 | 366.661 | 1.005 | Rio Grande do Norte |
| beta_week47 | 0.403 | 0.156 | 0.997 | 472.082 | 0.998 | Rio Grande do Norte |
| beta_week48 | 0.333 | 0.120 | 0.850 | 422.093 | 1.007 | Rio Grande do Norte |
| beta_week49 | 0.415 | 0.164 | 1.073 | 486.373 | 0.998 | Rio Grande do Norte |
| beta_week50 | 0.309 | 0.119 | 0.739 | 448.809 | 0.998 | Rio Grande do Norte |
| beta_week51 | 0.358 | 0.144 | 0.979 | 453.026 | 1.000 | Rio Grande do Norte |
| beta_week52 | 0.376 | 0.139 | 0.968 | 486.923 | 0.998 | Rio Grande do Norte |
| beta_week1 | 0.933 | 0.550 | 1.488 | 415.583 | 0.999 | Tocantins |
| beta_week2 | 0.570 | 0.176 | 1.321 | 421.908 | 1.007 | Tocantins |
| beta_week3 | 0.721 | 0.321 | 1.311 | 437.864 | 1.001 | Tocantins |
| beta_week4 | 0.734 | 0.246 | 1.346 | 501.246 | 0.998 | Tocantins |
| beta_week5 | 0.638 | 0.239 | 1.421 | 481.682 | 0.999 | Tocantins |
| beta_week6 | 1.477 | 0.756 | 2.432 | 468.259 | 0.998 | Tocantins |
| beta_week7 | 0.804 | 0.245 | 1.706 | 447.569 | 0.998 | Tocantins |
| beta_week8 | 1.560 | 0.833 | 2.660 | 310.379 | 1.001 | Tocantins |
| beta_week9 | 0.901 | 0.303 | 1.727 | 326.495 | 1.000 | Tocantins |
| beta_week10 | 2.028 | 1.294 | 3.043 | 316.242 | 1.001 | Tocantins |
| beta_week11 | 0.851 | 0.304 | 1.623 | 481.675 | 0.999 | Tocantins |
| beta_week12 | 1.103 | 0.645 | 1.817 | 427.645 | 1.004 | Tocantins |
| beta_week13 | 1.875 | 1.177 | 2.766 | 424.507 | 0.998 | Tocantins |
| beta_week14 | 1.380 | 0.788 | 2.159 | 447.546 | 0.999 | Tocantins |
| beta_week15 | 0.828 | 0.416 | 1.242 | 419.169 | 0.998 | Tocantins |
| beta_week16 | 0.614 | 0.330 | 0.994 | 462.030 | 1.007 | Tocantins |
| beta_week17 | 1.024 | 0.679 | 1.417 | 457.074 | 0.998 | Tocantins |
| beta_week18 | 0.432 | 0.195 | 0.791 | 528.318 | 1.000 | Tocantins |
| beta_week19 | 0.513 | 0.266 | 0.840 | 476.013 | 0.998 | Tocantins |
| beta_week20 | 1.083 | 0.733 | 1.515 | 533.827 | 1.000 | Tocantins |
| beta_week21 | 0.279 | 0.115 | 0.561 | 385.499 | 0.998 | Tocantins |
| beta_week22 | 0.313 | 0.155 | 0.527 | 469.313 | 1.000 | Tocantins |
| beta_week23 | 0.425 | 0.212 | 0.687 | 447.348 | 1.000 | Tocantins |
| beta_week24 | 0.375 | 0.178 | 0.636 | 354.687 | 1.003 | Tocantins |
| beta_week25 | 0.314 | 0.139 | 0.586 | 490.779 | 1.000 | Tocantins |
| beta_week26 | 0.455 | 0.219 | 0.767 | 422.721 | 1.005 | Tocantins |
| beta_week27 | 0.399 | 0.178 | 0.699 | 501.075 | 1.001 | Tocantins |
| beta_week28 | 0.278 | 0.117 | 0.554 | 498.315 | 0.998 | Tocantins |
| beta_week29 | 0.518 | 0.229 | 0.908 | 465.266 | 0.998 | Tocantins |
| beta_week30 | 0.434 | 0.157 | 0.845 | 493.187 | 1.004 | Tocantins |
| beta_week31 | 0.328 | 0.139 | 0.679 | 495.006 | 0.998 | Tocantins |
| beta_week32 | 0.482 | 0.195 | 0.979 | 265.872 | 0.998 | Tocantins |
| beta_week33 | 0.376 | 0.145 | 0.835 | 494.676 | 0.998 | Tocantins |
| beta_week34 | 0.310 | 0.125 | 0.640 | 584.982 | 0.999 | Tocantins |
| beta_week35 | 0.394 | 0.181 | 0.837 | 368.758 | 1.002 | Tocantins |
| beta_week36 | 0.409 | 0.162 | 0.837 | 467.195 | 1.002 | Tocantins |
| beta_week37 | 0.432 | 0.178 | 0.998 | 617.032 | 0.998 | Tocantins |
| beta_week38 | 0.382 | 0.154 | 0.970 | 421.434 | 1.000 | Tocantins |
| beta_week39 | 0.484 | 0.167 | 1.294 | 418.849 | 0.999 | Tocantins |
| beta_week40 | 0.524 | 0.186 | 1.138 | 426.793 | 0.998 | Tocantins |
| beta_week41 | 0.441 | 0.172 | 1.125 | 481.101 | 0.998 | Tocantins |
| beta_week42 | 0.922 | 0.207 | 2.415 | 337.444 | 0.998 | Tocantins |
| beta_week43 | 0.788 | 0.191 | 2.668 | 314.622 | 1.001 | Tocantins |
| beta_week44 | 1.135 | 0.278 | 3.745 | 306.871 | 1.000 | Tocantins |
| beta_week45 | 1.173 | 0.241 | 3.876 | 312.413 | 0.998 | Tocantins |
| beta_week46 | 1.106 | 0.253 | 2.643 | 227.738 | 1.002 | Tocantins |
| beta_week47 | 0.638 | 0.196 | 2.234 | 324.425 | 0.998 | Tocantins |
| beta_week48 | 2.318 | 0.615 | 4.567 | 318.626 | 1.008 | Tocantins |
| beta_week49 | 0.783 | 0.198 | 2.013 | 425.203 | 1.000 | Tocantins |
| beta_week50 | 0.324 | 0.139 | 0.715 | 437.372 | 0.999 | Tocantins |
| beta_week51 | 0.363 | 0.139 | 1.010 | 535.530 | 1.000 | Tocantins |
| beta_week52 | 0.366 | 0.151 | 0.964 | 435.189 | 1.000 | Tocantins |
| beta_week1 | 1.346 | 0.777 | 2.102 | 285.119 | 0.998 | Paraíba |
| beta_week2 | 0.645 | 0.211 | 2.078 | 411.422 | 0.998 | Paraíba |
| beta_week3 | 5.058 | 3.209 | 5.978 | 239.979 | 0.998 | Paraíba |
| beta_week4 | 0.966 | 0.235 | 3.394 | 381.981 | 1.004 | Paraíba |
| beta_week5 | 2.574 | 1.783 | 3.583 | 399.629 | 1.003 | Paraíba |
| beta_week6 | 0.504 | 0.189 | 1.206 | 441.993 | 0.998 | Paraíba |
| beta_week7 | 2.115 | 1.411 | 3.048 | 272.556 | 0.999 | Paraíba |
| beta_week8 | 0.744 | 0.267 | 1.773 | 406.203 | 0.999 | Paraíba |
| beta_week9 | 1.468 | 0.774 | 2.179 | 357.289 | 1.010 | Paraíba |
| beta_week10 | 0.935 | 0.341 | 1.739 | 445.108 | 0.998 | Paraíba |
| beta_week11 | 1.753 | 1.058 | 2.674 | 316.302 | 1.010 | Paraíba |
| beta_week12 | 1.945 | 1.116 | 2.717 | 428.754 | 0.999 | Paraíba |
| beta_week13 | 0.442 | 0.173 | 0.910 | 508.373 | 1.001 | Paraíba |
| beta_week14 | 0.793 | 0.432 | 1.346 | 382.795 | 1.001 | Paraíba |
| beta_week15 | 1.073 | 0.482 | 1.735 | 377.080 | 0.999 | Paraíba |
| beta_week16 | 1.512 | 0.918 | 2.103 | 410.117 | 1.000 | Paraíba |
| beta_week17 | 0.664 | 0.243 | 1.206 | 466.665 | 0.999 | Paraíba |
| beta_week18 | 0.660 | 0.316 | 1.070 | 466.373 | 0.998 | Paraíba |
| beta_week19 | 0.477 | 0.202 | 0.836 | 350.473 | 0.998 | Paraíba |
| beta_week20 | 0.484 | 0.241 | 0.863 | 428.598 | 0.998 | Paraíba |
| beta_week21 | 0.595 | 0.280 | 0.974 | 434.127 | 1.000 | Paraíba |
| beta_week22 | 0.563 | 0.245 | 0.962 | 438.757 | 1.003 | Paraíba |
| beta_week23 | 0.610 | 0.281 | 1.051 | 357.606 | 1.000 | Paraíba |
| beta_week24 | 0.910 | 0.408 | 1.404 | 371.397 | 0.999 | Paraíba |
| beta_week25 | 0.558 | 0.239 | 1.068 | 455.881 | 0.999 | Paraíba |
| beta_week26 | 0.906 | 0.469 | 1.343 | 344.683 | 1.000 | Paraíba |
| beta_week27 | 0.410 | 0.158 | 0.804 | 460.572 | 1.000 | Paraíba |
| beta_week28 | 0.355 | 0.177 | 0.640 | 458.863 | 0.998 | Paraíba |
| beta_week29 | 0.563 | 0.276 | 0.912 | 506.908 | 1.001 | Paraíba |
| beta_week30 | 0.524 | 0.221 | 0.885 | 350.099 | 0.998 | Paraíba |
| beta_week31 | 0.362 | 0.160 | 0.628 | 363.034 | 0.998 | Paraíba |
| beta_week32 | 0.331 | 0.145 | 0.610 | 457.679 | 0.998 | Paraíba |
| beta_week33 | 0.321 | 0.147 | 0.561 | 453.692 | 0.998 | Paraíba |
| beta_week34 | 0.426 | 0.178 | 0.771 | 396.456 | 1.000 | Paraíba |
| beta_week35 | 0.367 | 0.164 | 0.717 | 458.533 | 1.004 | Paraíba |
| beta_week36 | 0.323 | 0.133 | 0.655 | 446.445 | 0.998 | Paraíba |
| beta_week37 | 0.381 | 0.160 | 0.701 | 491.151 | 0.999 | Paraíba |
| beta_week38 | 0.417 | 0.160 | 0.822 | 491.753 | 0.999 | Paraíba |
| beta_week39 | 0.316 | 0.124 | 0.622 | 488.781 | 1.000 | Paraíba |
| beta_week40 | 0.379 | 0.146 | 0.749 | 476.775 | 0.998 | Paraíba |
| beta_week41 | 0.380 | 0.149 | 0.799 | 423.927 | 1.000 | Paraíba |
| beta_week42 | 0.399 | 0.152 | 0.896 | 379.527 | 1.019 | Paraíba |
| beta_week43 | 0.308 | 0.121 | 0.637 | 447.234 | 0.999 | Paraíba |
| beta_week44 | 0.318 | 0.133 | 0.712 | 493.604 | 0.999 | Paraíba |
| beta_week45 | 0.423 | 0.175 | 0.972 | 418.180 | 0.998 | Paraíba |
| beta_week46 | 0.361 | 0.135 | 0.892 | 468.089 | 0.998 | Paraíba |
| beta_week47 | 0.355 | 0.127 | 0.805 | 361.027 | 0.998 | Paraíba |
| beta_week48 | 0.350 | 0.129 | 0.790 | 505.768 | 1.002 | Paraíba |
| beta_week49 | 0.374 | 0.146 | 0.959 | 367.380 | 0.999 | Paraíba |
| beta_week50 | 0.327 | 0.136 | 0.792 | 500.383 | 0.998 | Paraíba |
| beta_week51 | 0.380 | 0.142 | 0.993 | 394.176 | 0.998 | Paraíba |
| beta_week52 | 0.358 | 0.134 | 0.891 | 356.409 | 1.001 | Paraíba |
| beta_week1 | 0.669 | 0.305 | 1.258 | 268.443 | 0.998 | Sergipe |
| beta_week2 | 0.492 | 0.151 | 1.370 | 445.793 | 0.999 | Sergipe |
| beta_week3 | 2.026 | 0.384 | 4.950 | 307.677 | 0.998 | Sergipe |
| beta_week4 | 1.867 | 0.286 | 4.324 | 341.440 | 0.999 | Sergipe |
| beta_week5 | 0.703 | 0.222 | 1.885 | 460.422 | 0.998 | Sergipe |
| beta_week6 | 0.656 | 0.209 | 1.704 | 435.198 | 1.002 | Sergipe |
| beta_week7 | 1.459 | 0.495 | 2.656 | 528.103 | 0.998 | Sergipe |
| beta_week8 | 0.674 | 0.232 | 1.877 | 403.997 | 1.001 | Sergipe |
| beta_week9 | 0.949 | 0.293 | 1.969 | 308.017 | 1.002 | Sergipe |
| beta_week10 | 0.938 | 0.262 | 1.997 | 363.309 | 0.999 | Sergipe |
| beta_week11 | 0.533 | 0.207 | 1.325 | 491.166 | 0.998 | Sergipe |
| beta_week12 | 0.812 | 0.293 | 1.805 | 392.651 | 1.003 | Sergipe |
| beta_week13 | 0.842 | 0.260 | 1.969 | 415.553 | 0.998 | Sergipe |
| beta_week14 | 1.179 | 0.309 | 2.170 | 396.792 | 0.998 | Sergipe |
| beta_week15 | 0.467 | 0.183 | 1.124 | 401.011 | 0.999 | Sergipe |
| beta_week16 | 0.750 | 0.200 | 1.813 | 454.995 | 0.998 | Sergipe |
| beta_week17 | 2.306 | 0.832 | 4.687 | 320.174 | 0.998 | Sergipe |
| beta_week18 | 1.796 | 0.376 | 3.208 | 307.601 | 1.000 | Sergipe |
| beta_week19 | 0.532 | 0.168 | 1.136 | 445.589 | 0.998 | Sergipe |
| beta_week20 | 0.596 | 0.248 | 1.094 | 440.496 | 1.003 | Sergipe |
| beta_week21 | 0.601 | 0.245 | 1.180 | 505.570 | 0.999 | Sergipe |
| beta_week22 | 0.591 | 0.230 | 1.205 | 412.896 | 1.003 | Sergipe |
| beta_week23 | 0.622 | 0.243 | 1.200 | 386.210 | 0.998 | Sergipe |
| beta_week24 | 0.580 | 0.236 | 1.148 | 501.967 | 1.004 | Sergipe |
| beta_week25 | 0.649 | 0.249 | 1.329 | 348.736 | 1.001 | Sergipe |
| beta_week26 | 1.019 | 0.332 | 1.773 | 288.838 | 0.998 | Sergipe |
| beta_week27 | 0.527 | 0.183 | 1.233 | 496.636 | 1.006 | Sergipe |
| beta_week28 | 0.670 | 0.227 | 1.429 | 556.329 | 1.000 | Sergipe |
| beta_week29 | 0.958 | 0.315 | 1.718 | 500.656 | 1.001 | Sergipe |
| beta_week30 | 0.491 | 0.168 | 1.034 | 424.401 | 0.998 | Sergipe |
| beta_week31 | 0.509 | 0.204 | 0.974 | 406.438 | 1.000 | Sergipe |
| beta_week32 | 0.498 | 0.179 | 0.934 | 325.190 | 0.999 | Sergipe |
| beta_week33 | 0.352 | 0.163 | 0.687 | 453.781 | 1.002 | Sergipe |
| beta_week34 | 0.377 | 0.165 | 0.735 | 464.522 | 0.998 | Sergipe |
| beta_week35 | 0.577 | 0.223 | 1.071 | 356.835 | 0.998 | Sergipe |
| beta_week36 | 0.422 | 0.147 | 0.911 | 419.730 | 0.999 | Sergipe |
| beta_week37 | 0.395 | 0.160 | 0.828 | 383.291 | 1.000 | Sergipe |
| beta_week38 | 0.400 | 0.152 | 0.911 | 362.131 | 0.998 | Sergipe |
| beta_week39 | 0.313 | 0.133 | 0.709 | 401.052 | 0.999 | Sergipe |
| beta_week40 | 0.348 | 0.143 | 0.727 | 388.995 | 1.000 | Sergipe |
| beta_week41 | 0.474 | 0.159 | 1.151 | 441.797 | 0.998 | Sergipe |
| beta_week42 | 0.790 | 0.254 | 1.723 | 451.229 | 0.998 | Sergipe |
| beta_week43 | 0.551 | 0.186 | 1.395 | 413.280 | 1.000 | Sergipe |
| beta_week44 | 0.471 | 0.158 | 1.083 | 406.649 | 0.998 | Sergipe |
| beta_week45 | 0.657 | 0.229 | 1.506 | 501.743 | 0.998 | Sergipe |
| beta_week46 | 0.562 | 0.200 | 1.332 | 457.994 | 0.998 | Sergipe |
| beta_week47 | 0.525 | 0.184 | 1.232 | 392.919 | 1.000 | Sergipe |
| beta_week48 | 0.575 | 0.194 | 1.425 | 426.344 | 0.999 | Sergipe |
| beta_week49 | 0.461 | 0.167 | 1.110 | 317.792 | 0.998 | Sergipe |
| beta_week50 | 0.342 | 0.132 | 0.760 | 378.162 | 0.998 | Sergipe |
| beta_week51 | 0.346 | 0.132 | 0.938 | 315.145 | 1.002 | Sergipe |
| beta_week52 | 0.357 | 0.154 | 0.913 | 389.921 | 1.001 | Sergipe |

Supplementary figure11 Vaccine distribution process

The figure illustrates the vaccine distribution process. The total vaccine stockpile (available at each geographic region) is first determined by the vaccine coverage. In our base-case analysis, we assumed 50% of vaccine coverage of the total targeted population size in each state. Age-specific total amount of vaccines is determined by the demographic structure. Each week, vaccine is distributed based on the weekly delivery speed (base-case: 10%) to each age group, following pro-rata strategy.


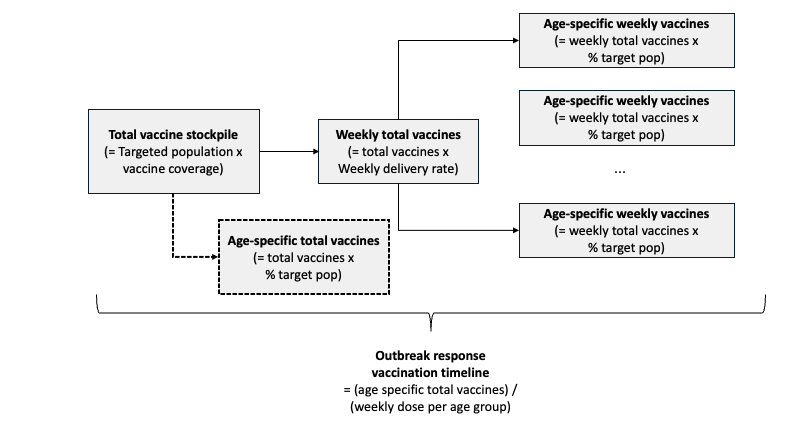


Supplementary figure 12 Vaccine doses by age group, week, and subnational state (target coverage 50%)


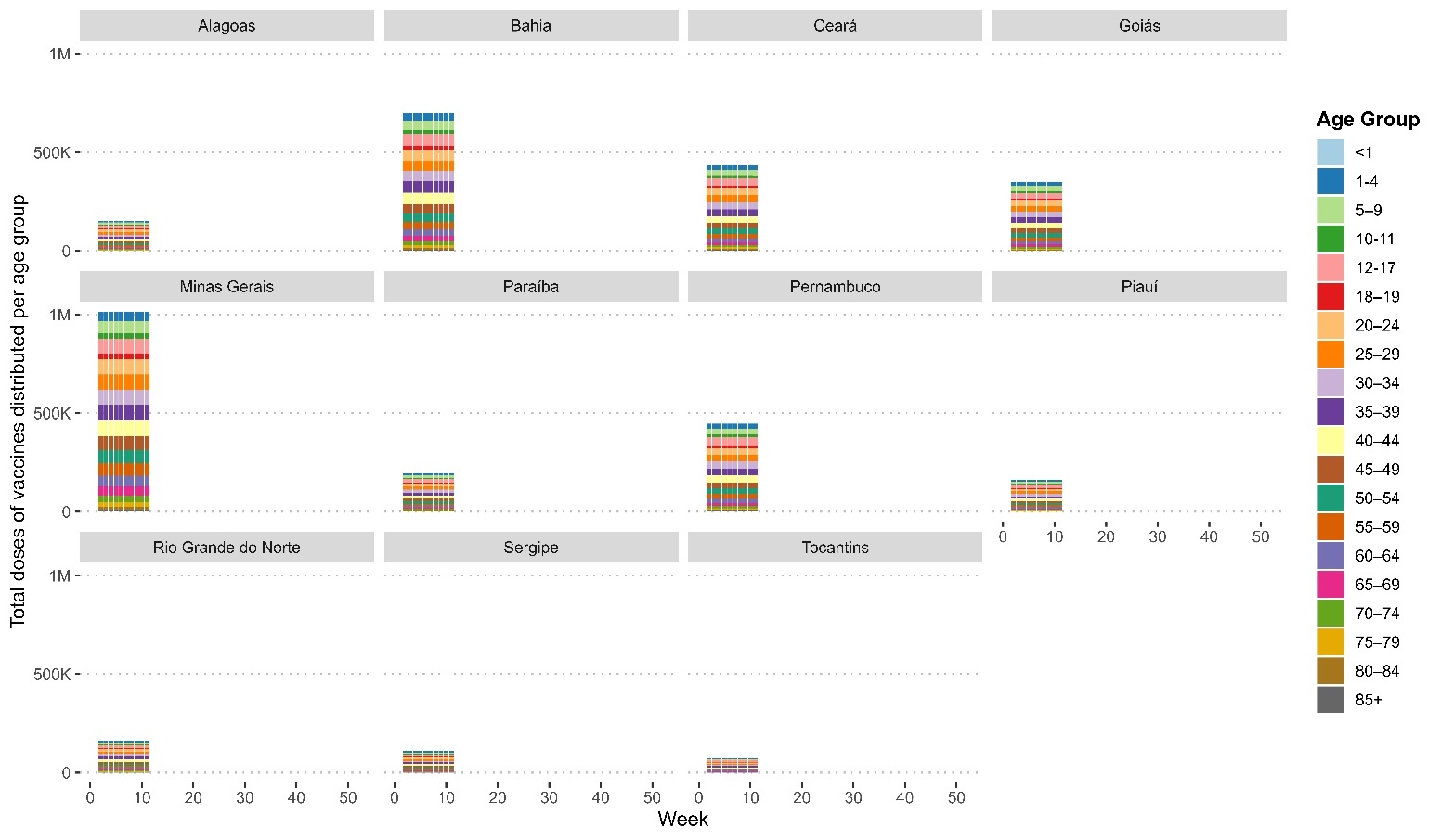


Supplementary figure13 State-level with- and without vaccination epidemic curves (disease-blocking efficacy)


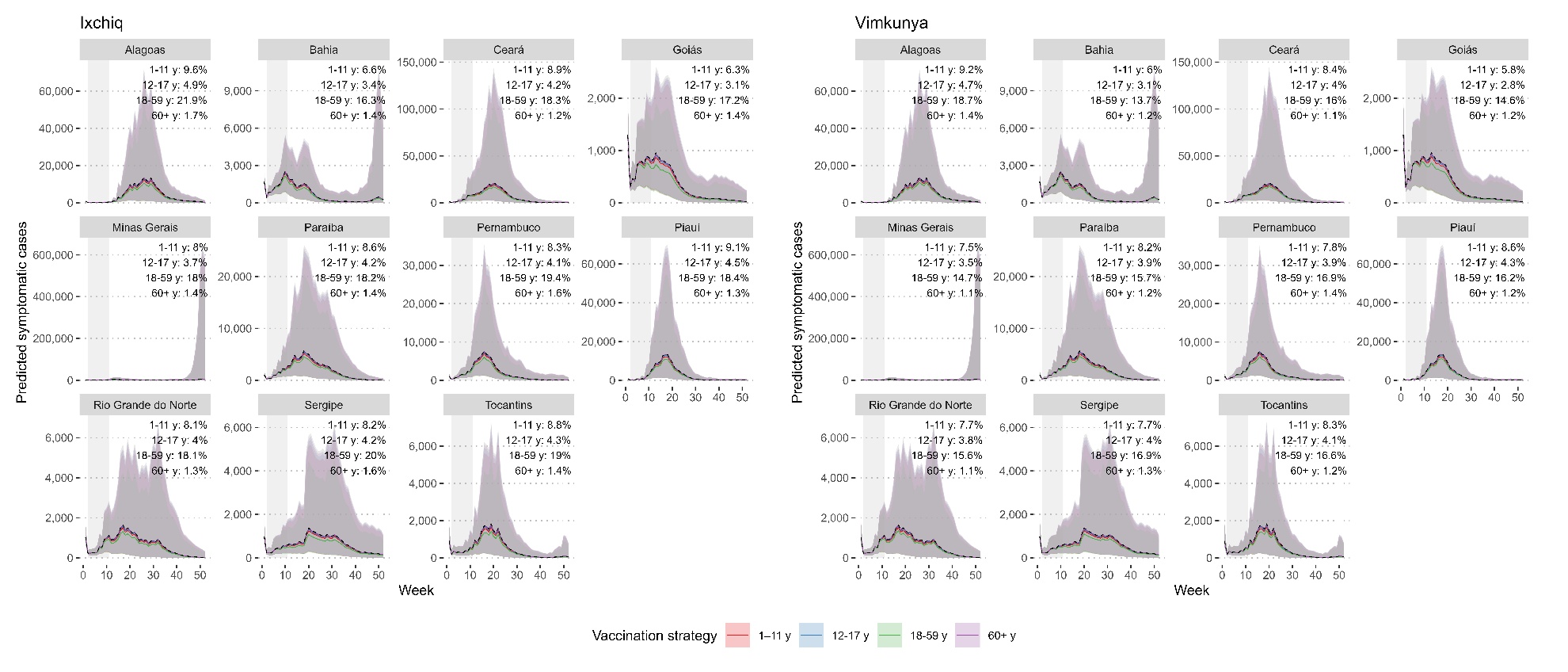


Supplementary figure14 State-level with- and without vaccination epidemic curves (disease and infection blocking efficacy)


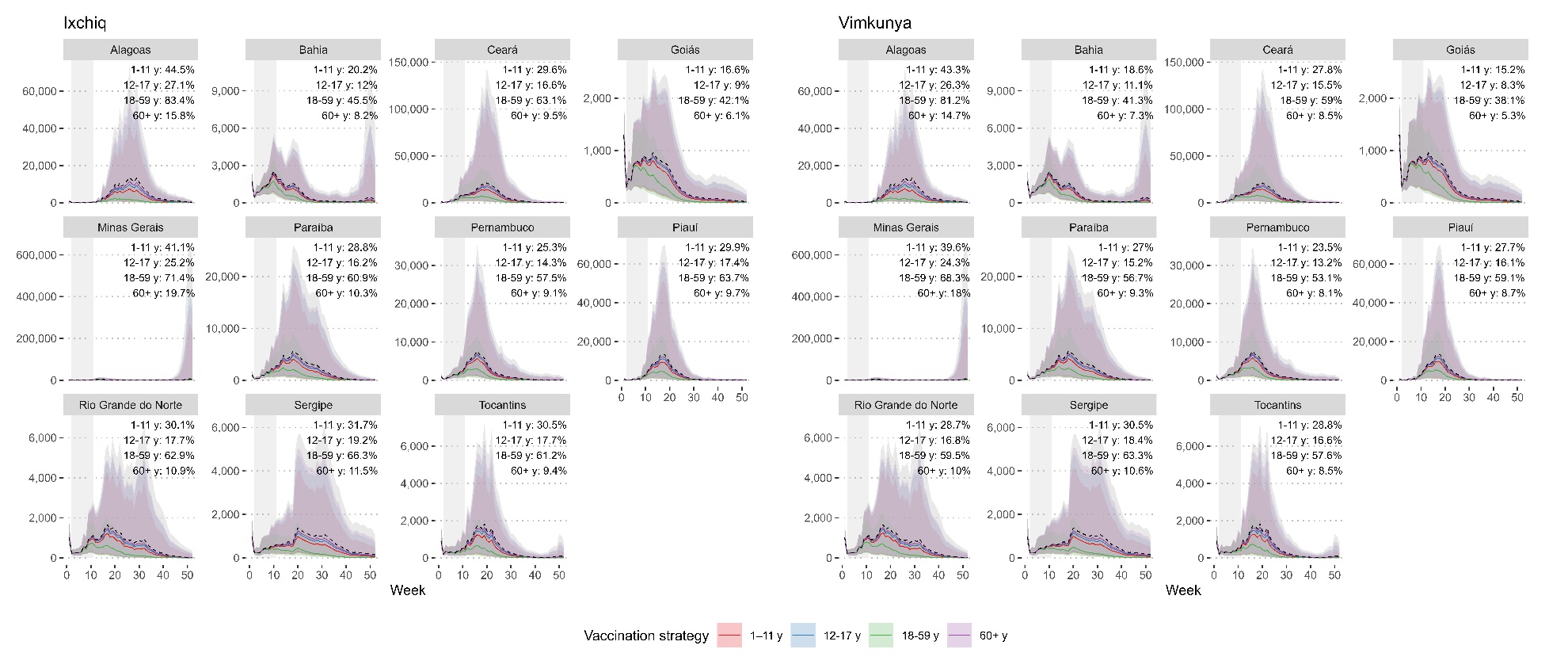


Supplementary figure15 Number needed to avert a single symptomatic, fatal case, and DALY by vaccination scenario, region, age group (Ixchiq)


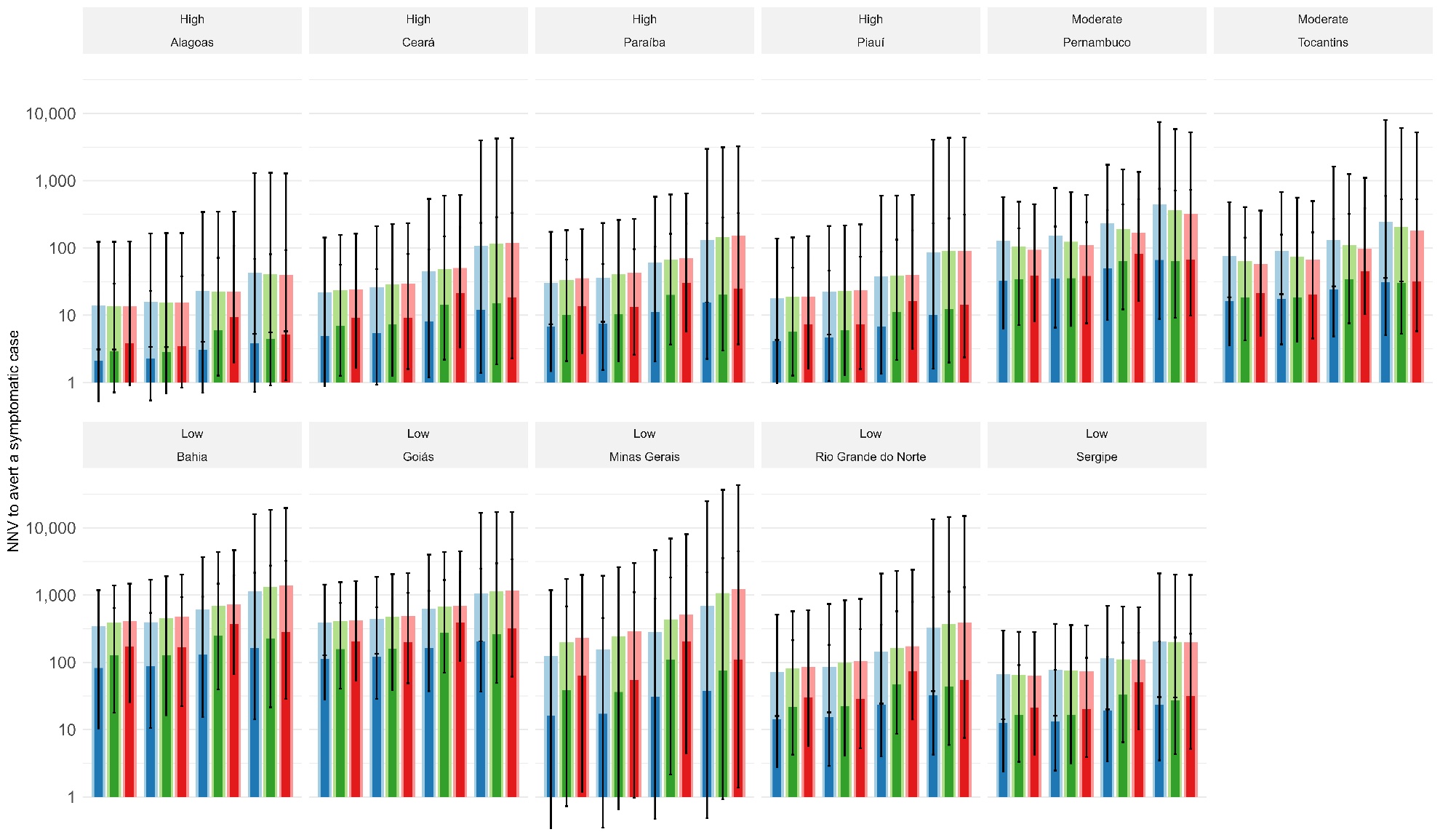


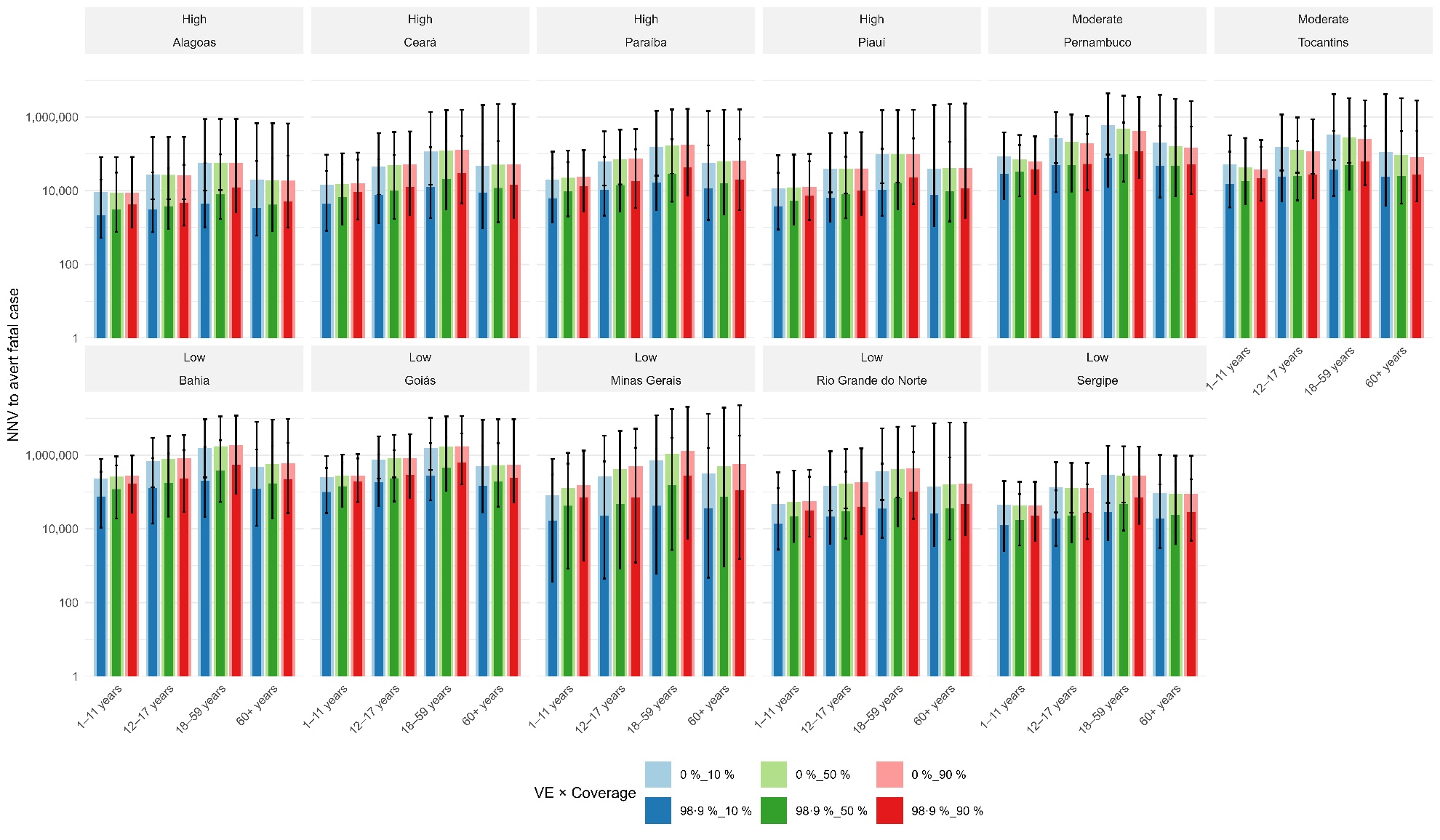


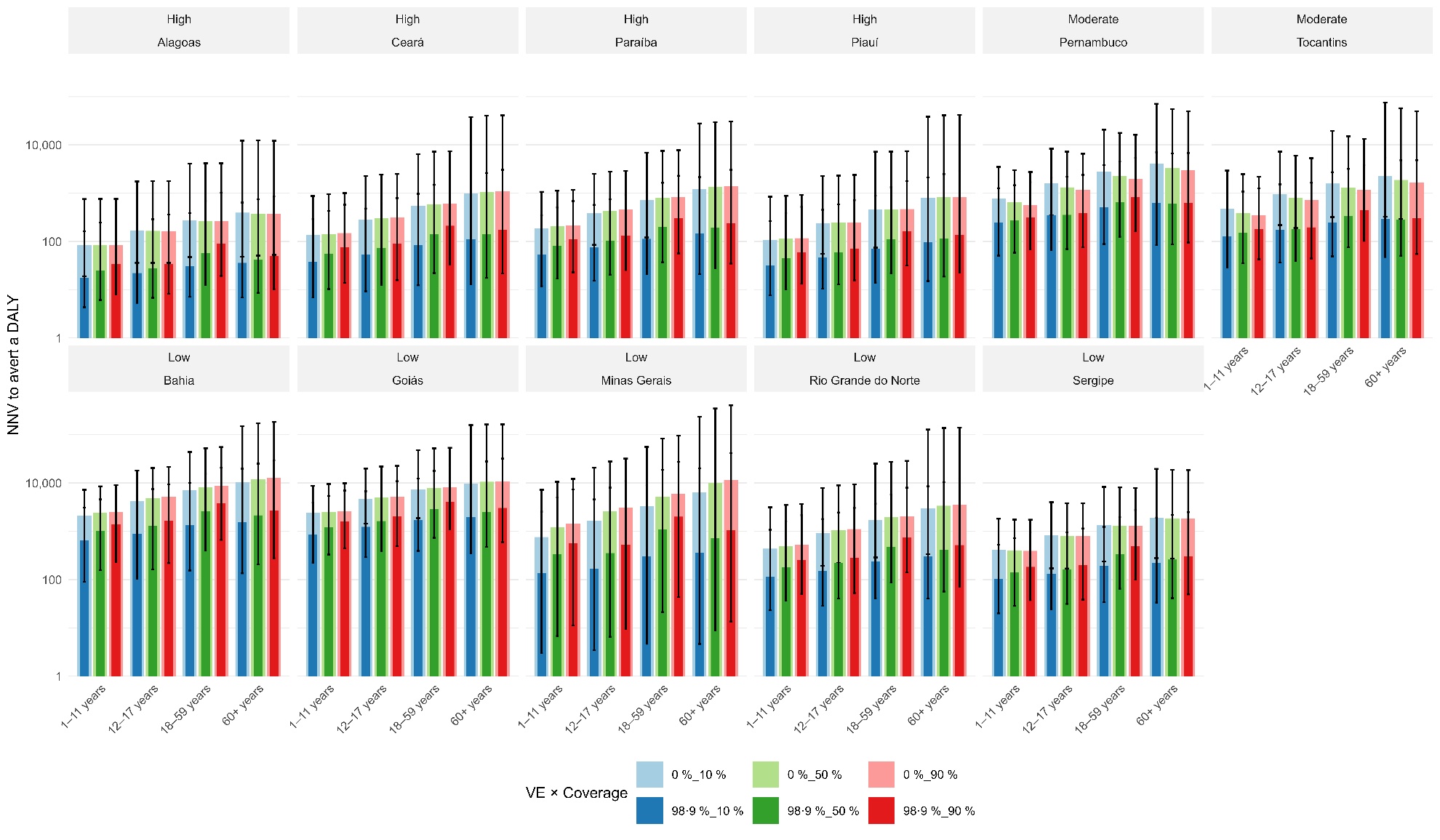


Supplementary figure16 Number needed to avert a single symptomatic, fatal case, and DALY by vaccination scenario, region, age group (Vimkunya)


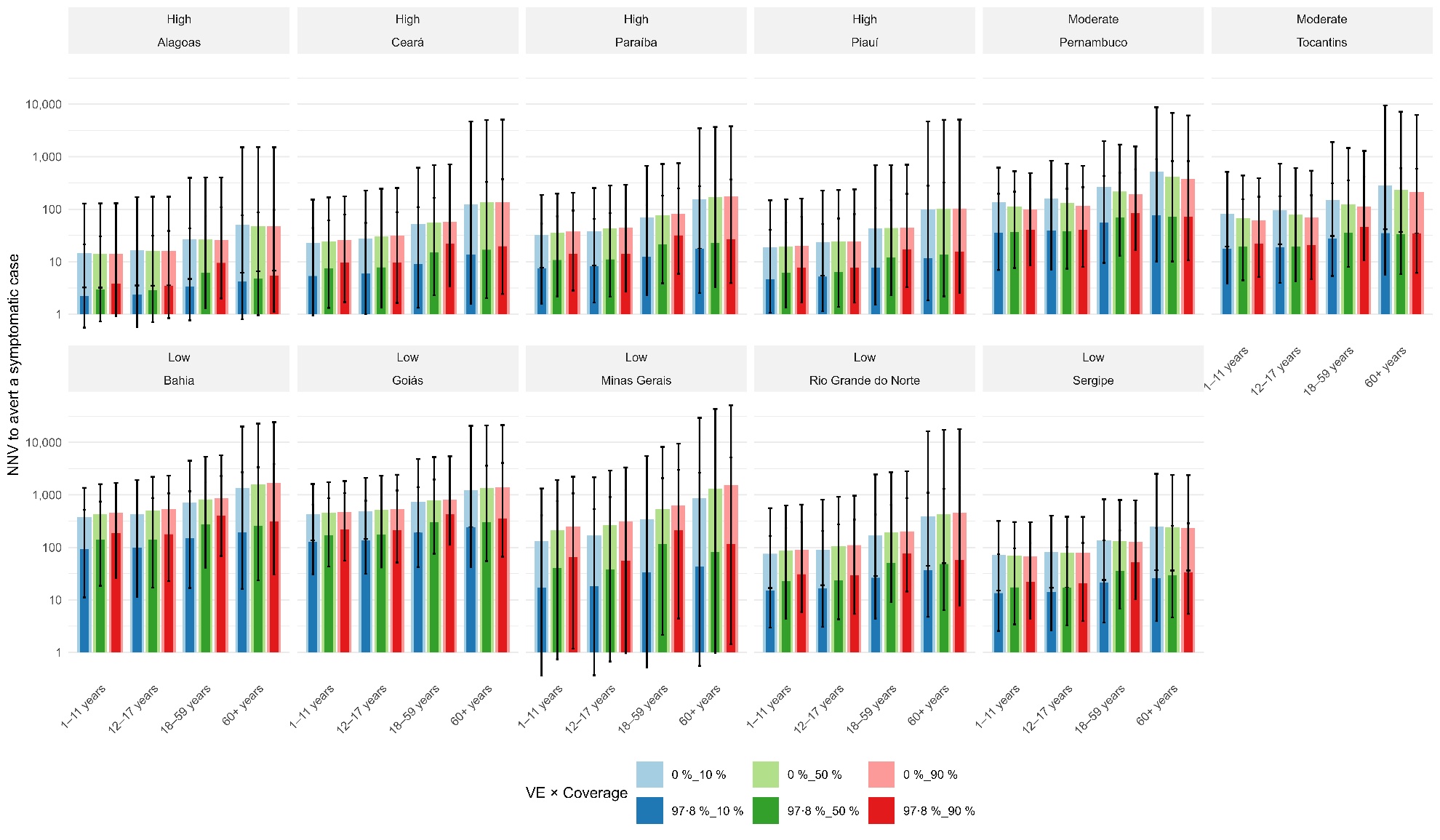


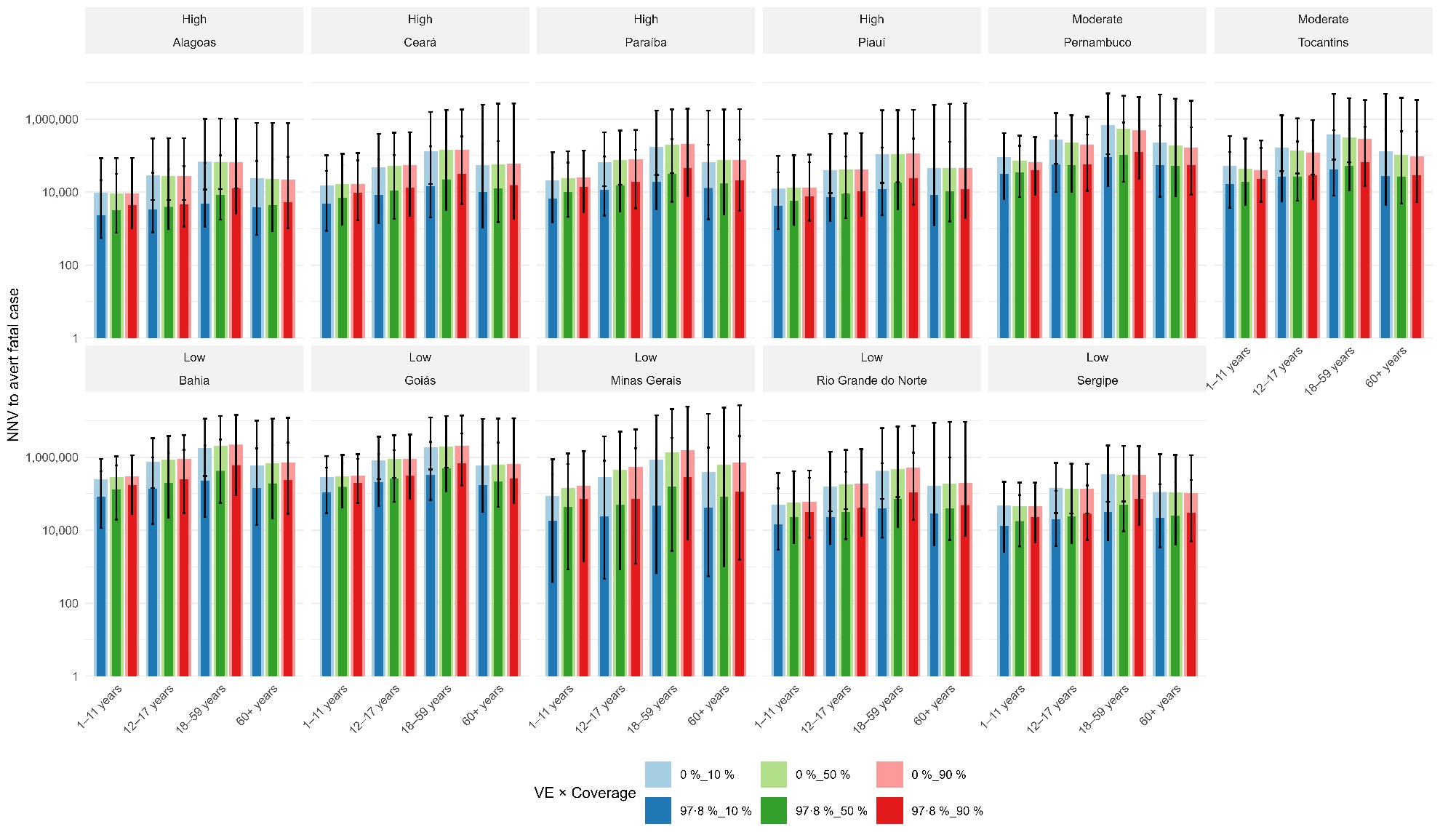


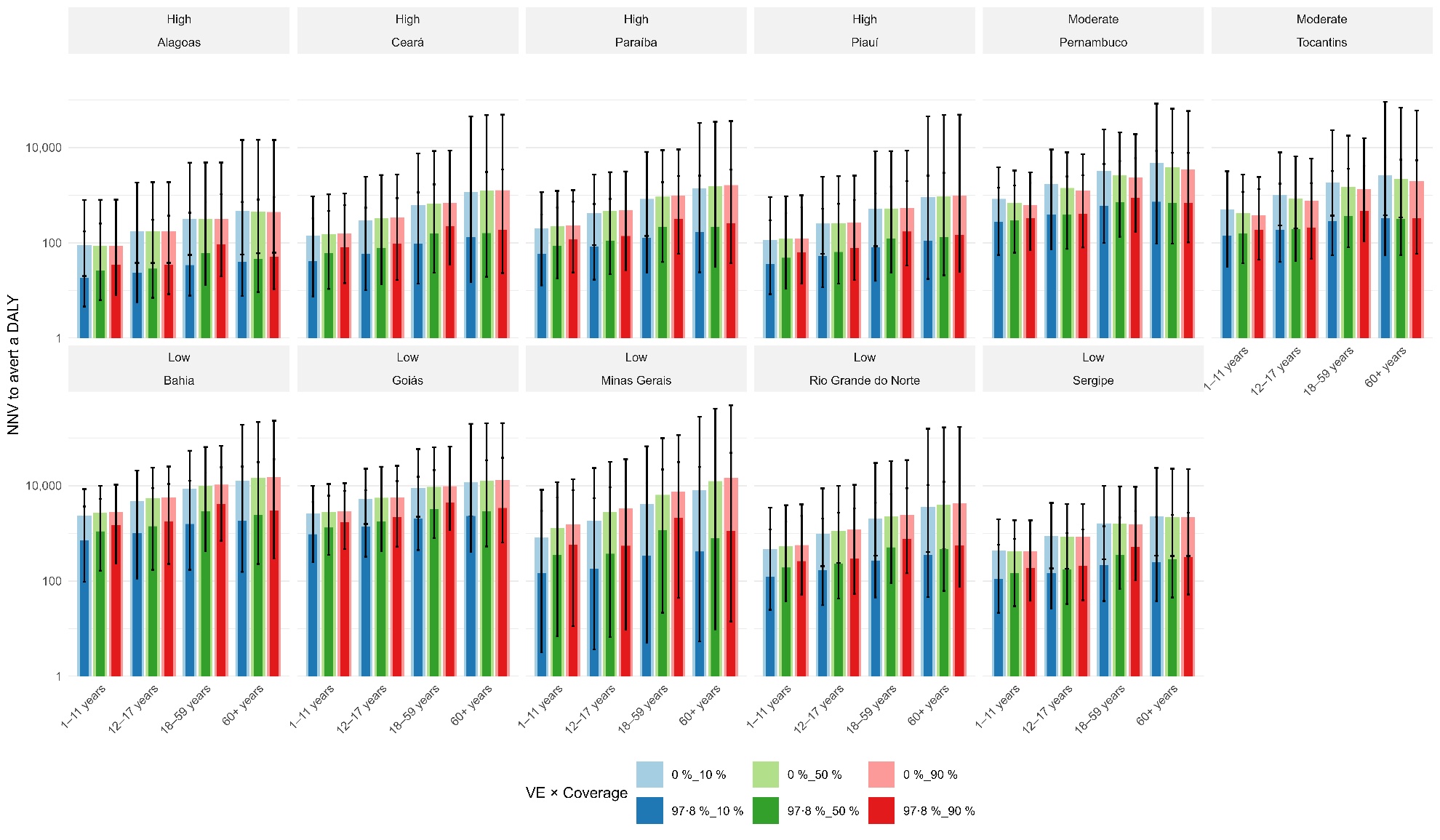


Supplementary figure17 One-way sensitivity results by vaccination strategy and region

For the base-case vaccine impact values (% case reduction), we used 95% median of FOI as underlying immunity, 75% as vaccine efficacy, and 10% as weekly delivery speed. To test the model sensitivity to each parameter, we used 95% UI values (2.5^th^ and 97.5^th^ percentiles) of FOI, 96.7% and 99.8% for vaccine efficacy, and ±10% around weekly delivery speed, and vaccine coverage. Time until immunity and delay were varied by 1 week (lower bound) and 3 weeks (upper bound).

**
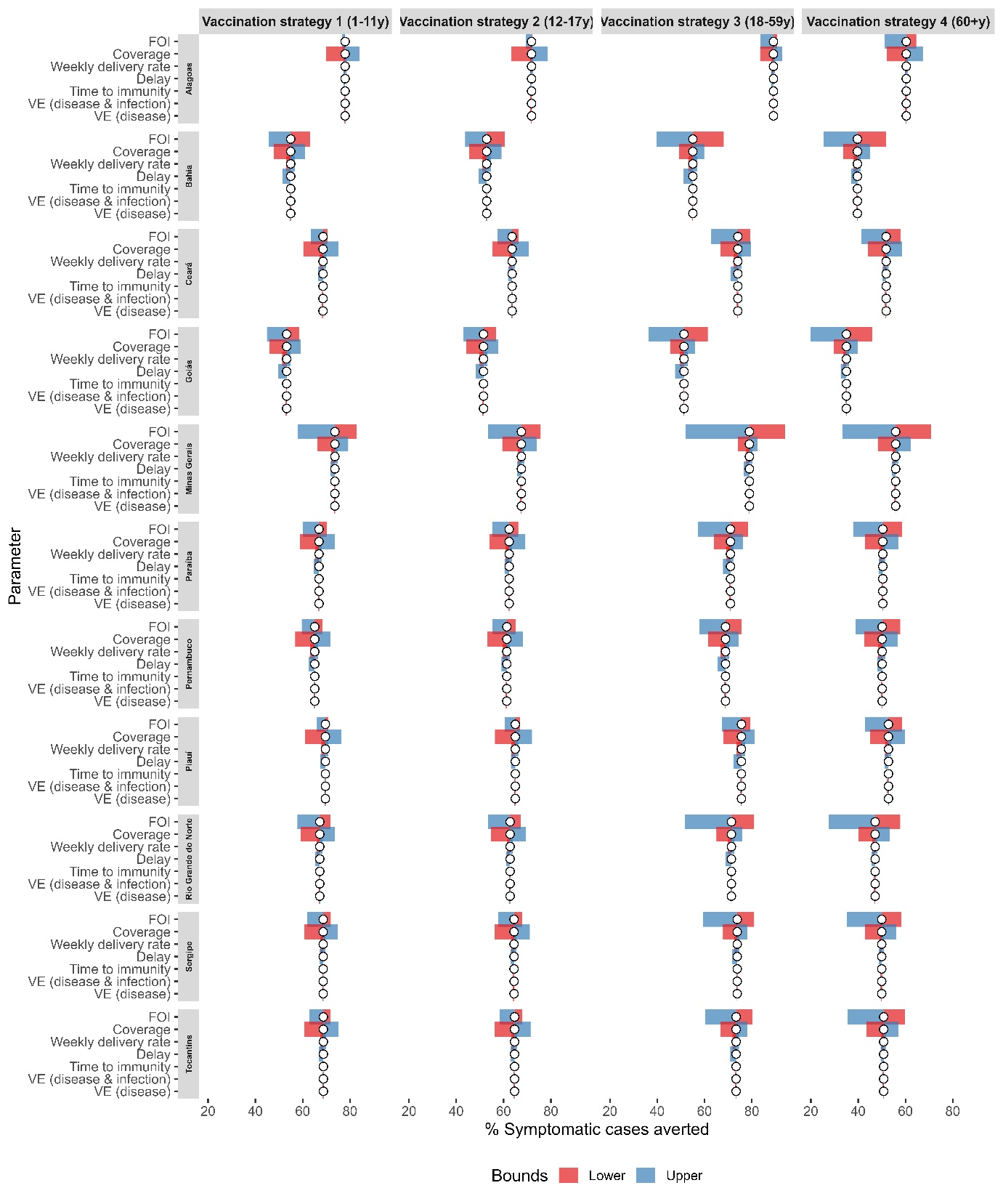
**

Supplementary figure 18 One-way sensitivity results by vaccination strategy at national level


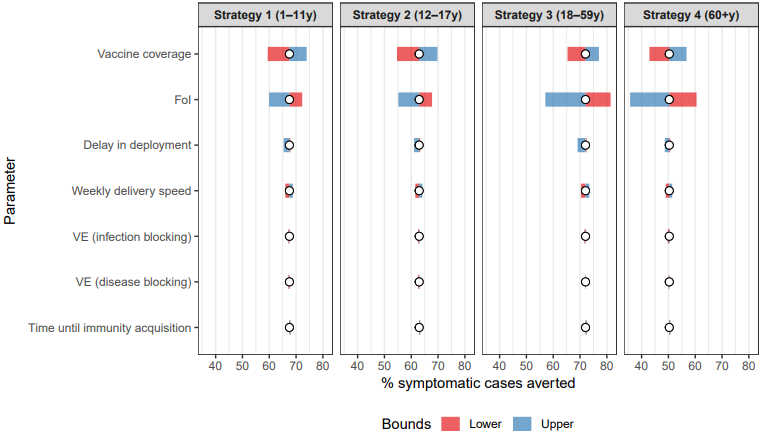


Supplementary figure 19 Vaccine coverage and delay scenario analyses (deployment weeks varied from week1 to 52 by 1 week, and vaccine coverage varied from 10% to 100% by 10%)


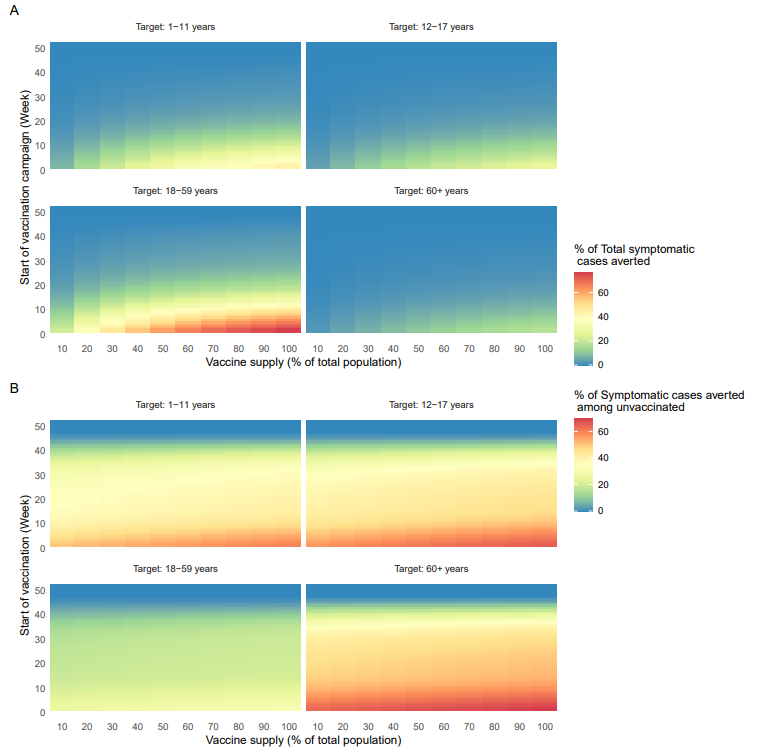


Supplementary table6 NNVs at sub-national level (Ixchiq)

| **Setting** | **Scenario** | **Region** | **Total vaccines** | **NNV (infection)** | **NNV (infection; 95% lo)** | **NNV (infection; 95% hi)** | **NNV (symptomatic)** | **NNV (symptomatic; 95% lo)** | **NNV (symptomatic; 95% hi)** | **NNV (Death)** | **NNV (Death; 95% lo)** | **NNV (Death; 95% hi)** | **NNV (DALY)** | **NNV (DALY; 95% lo)** | **NNV (DALY; 95% hi)** | **VEVC** |
| --- | --- | --- | --- | --- | --- | --- | --- | --- | --- | --- | --- | --- | --- | --- | --- | --- |
| High | 1–11 years | Alagoas | 52,717 | Inf | Inf | Inf | 14 | 3 | 123 | 9,228 | 2,059 | 81,763 | 86 | 19 | 760 | 0 %_10 % |
| High | 1–11 years | Ceará | 115,930 | Inf | Inf | Inf | 22 | 4 | 143 | 14,594 | 2,598 | 94,570 | 136 | 24 | 877 | 0 %_10 % |
| High | 1–11 years | Paraíba | 49,504 | Inf | Inf | Inf | 30 | 7 | 174 | 20,106 | 4,875 | 115,269 | 187 | 45 | 1,070 | 0 %_10 % |
| High | 1–11 years | Piauí | 45,738 | Inf | Inf | Inf | 18 | 4 | 138 | 11,745 | 2,843 | 91,613 | 109 | 26 | 850 | 0 %_10 % |
| High | 12–17 years | Alagoas | 31,096 | Inf | Inf | Inf | 16 | 3 | 164 | 27,100 | 5,855 | 283,413 | 168 | 36 | 1,759 | 0 %_10 % |
| High | 12–17 years | Ceará | 67,573 | Inf | Inf | Inf | 26 | 4 | 211 | 45,452 | 7,539 | 365,327 | 282 | 47 | 2,267 | 0 %_10 % |
| High | 12–17 years | Paraíba | 28,709 | Inf | Inf | Inf | 36 | 8 | 235 | 62,863 | 13,759 | 405,809 | 390 | 85 | 2,518 | 0 %_10 % |
| High | 12–17 years | Piauí | 27,894 | Inf | Inf | Inf | 22 | 5 | 212 | 38,644 | 8,968 | 366,885 | 240 | 56 | 2,277 | 0 %_10 % |
| High | 18–59 years | Alagoas | 201,650 | Inf | Inf | Inf | 23 | 4 | 344 | 57,811 | 10,084 | 878,332 | 274 | 48 | 4,132 | 0 %_10 % |
| High | 18–59 years | Ceará | 499,520 | Inf | Inf | Inf | 45 | 6 | 536 | 115,041 | 14,549 | 1,370,062 | 543 | 69 | 6,480 | 0 %_10 % |
| High | 18–59 years | Paraíba | 207,118 | Inf | Inf | Inf | 60 | 10 | 577 | 152,518 | 25,557 | 1,472,118 | 722 | 121 | 6,960 | 0 %_10 % |
| High | 18–59 years | Piauí | 192,626 | Inf | Inf | Inf | 38 | 6 | 600 | 96,499 | 15,795 | 1,516,268 | 457 | 75 | 7,235 | 0 %_10 % |
| High | 60+ years | Alagoas | 28,494 | Inf | Inf | Inf | 42 | 5 | 1,300 | 19,979 | 2,332 | 676,479 | 396 | 48 | 12,323 | 0 %_10 % |
| High | 60+ years | Ceará | 80,143 | Inf | Inf | Inf | 108 | 9 | 3,987 | 47,436 | 3,644 | 2,111,396 | 994 | 82 | 37,912 | 0 %_10 % |
| High | 60+ years | Paraíba | 35,889 | Inf | Inf | Inf | 132 | 15 | 2,976 | 57,502 | 6,237 | 1,452,313 | 1,211 | 138 | 27,898 | 0 %_10 % |
| High | 60+ years | Piauí | 32,227 | Inf | Inf | Inf | 87 | 9 | 4,067 | 39,415 | 3,900 | 2,108,122 | 801 | 84 | 38,538 | 0 %_10 % |
| Low | 1–11 years | Bahia | 167,710 | Inf | Inf | Inf | 344 | 72 | 1,181 | 228,255 | 48,024 | 783,272 | 2,123 | 447 | 7,278 | 0 %_10 % |
| Low | 1–11 years | Goiás | 94,119 | Inf | Inf | Inf | 390 | 127 | 1,435 | 258,596 | 84,153 | 951,419 | 2,403 | 783 | 8,836 | 0 %_10 % |
| Low | 1–11 years | Minas Gerais | 141,693 | Inf | Inf | Inf | 124 | 4 | 1,187 | 82,110 | 2,693 | 787,225 | 763 | 25 | 7,303 | 0 %_10 % |
| Low | 1–11 years | Rio Grande do Norte | 39,752 | Inf | Inf | Inf | 71 | 16 | 512 | 47,237 | 10,550 | 339,144 | 439 | 98 | 3,149 | 0 %_10 % |
| Low | 1–11 years | Sergipe | 35,934 | Inf | Inf | Inf | 67 | 14 | 297 | 44,502 | 9,454 | 197,011 | 414 | 88 | 1,831 | 0 %_10 % |
| Low | 12–17 years | Bahia | 100,511 | Inf | Inf | Inf | 396 | 77 | 1,691 | 684,282 | 133,583 | 2,923,218 | 4,247 | 829 | 18,142 | 0 %_10 % |
| Low | 12–17 years | Goiás | 52,263 | Inf | Inf | Inf | 444 | 135 | 1,865 | 767,082 | 232,813 | 3,225,250 | 4,761 | 1,445 | 20,016 | 0 %_10 % |
| Low | 12–17 years | Minas Gerais | 81,631 | Inf | Inf | Inf | 156 | 5 | 1,938 | 269,644 | 7,888 | 3,350,285 | 1,673 | 49 | 20,792 | 0 %_10 % |
| Low | 12–17 years | Rio Grande do Norte | 23,721 | Inf | Inf | Inf | 85 | 18 | 737 | 146,834 | 31,165 | 1,274,684 | 911 | 193 | 7,911 | 0 %_10 % |
| Low | 12–17 years | Sergipe | 21,541 | Inf | Inf | Inf | 77 | 16 | 374 | 132,867 | 27,734 | 646,204 | 825 | 172 | 4,010 | 0 %_10 % |
| Low | 18–59 years | Bahia | 734,611 | Inf | Inf | Inf | 608 | 100 | 3,684 | 1,531,213 | 249,224 | 9,380,111 | 7,237 | 1,183 | 44,317 | 0 %_10 % |
| Low | 18–59 years | Goiás | 415,700 | Inf | Inf | Inf | 625 | 158 | 3,993 | 1,577,959 | 395,126 | 10,244,657 | 7,448 | 1,873 | 48,123 | 0 %_10 % |
| Low | 18–59 years | Minas Gerais | 701,046 | Inf | Inf | Inf | 280 | 6 | 4,677 | 708,948 | 15,126 | 11,950,743 | 3,347 | 72 | 56,386 | 0 %_10 % |
| Low | 18–59 years | Rio Grande do Norte | 179,202 | Inf | Inf | Inf | 144 | 24 | 2,089 | 363,524 | 61,033 | 5,340,147 | 1,722 | 290 | 25,259 | 0 %_10 % |
| Low | 18–59 years | Sergipe | 150,723 | Inf | Inf | Inf | 114 | 20 | 696 | 287,975 | 50,378 | 1,774,379 | 1,359 | 238 | 8,371 | 0 %_10 % |
| Low | 60+ years | Bahia | 121,111 | Inf | Inf | Inf | 1,134 | 139 | 16,024 | 488,271 | 56,058 | 8,049,543 | 10,377 | 1,253 | 151,018 | 0 %_10 % |
| Low | 60+ years | Goiás | 57,694 | Inf | Inf | Inf | 1,051 | 203 | 16,701 | 492,530 | 91,027 | 9,053,839 | 9,776 | 1,875 | 159,470 | 0 %_10 % |
| Low | 60+ years | Minas Gerais | 132,262 | Inf | Inf | Inf | 697 | 9 | 24,883 | 323,635 | 3,652 | 13,153,721 | 6,474 | 80 | 236,604 | 0 %_10 % |
| Low | 60+ years | Rio Grande do Norte | 28,886 | Inf | Inf | Inf | 329 | 37 | 13,460 | 142,163 | 15,033 | 7,270,330 | 3,010 | 337 | 128,437 | 0 %_10 % |
| Low | 60+ years | Sergipe | 21,237 | Inf | Inf | Inf | 206 | 30 | 2,108 | 93,533 | 13,466 | 1,012,670 | 1,908 | 280 | 19,703 | 0 %_10 % |
| Moderate | 1–11 years | Pernambuco | 196,549 | Inf | Inf | Inf | 127 | 29 | 572 | 84,303 | 19,257 | 379,335 | 784 | 179 | 3,523 | 0 %_10 % |
| Moderate | 1–11 years | Tocantins | 35,864 | Inf | Inf | Inf | 77 | 18 | 478 | 50,884 | 12,258 | 317,020 | 473 | 114 | 2,944 | 0 %_10 % |
| Moderate | 12–17 years | Pernambuco | 114,240 | Inf | Inf | Inf | 152 | 32 | 781 | 262,753 | 56,137 | 1,349,835 | 1,631 | 348 | 8,377 | 0 %_10 % |
| Moderate | 12–17 years | Tocantins | 20,633 | Inf | Inf | Inf | 90 | 20 | 676 | 155,485 | 35,444 | 1,169,716 | 965 | 220 | 7,259 | 0 %_10 % |
| Moderate | 18–59 years | Pernambuco | 825,774 | Inf | Inf | Inf | 234 | 38 | 1,726 | 588,895 | 95,978 | 4,381,068 | 2,788 | 455 | 20,742 | 0 %_10 % |
| Moderate | 18–59 years | Tocantins | 133,047 | Inf | Inf | Inf | 133 | 27 | 1,628 | 336,276 | 67,648 | 4,163,303 | 1,585 | 320 | 19,627 | 0 %_10 % |
| Moderate | 60+ years | Pernambuco | 130,770 | Inf | Inf | Inf | 447 | 55 | 7,463 | 200,441 | 23,001 | 4,000,296 | 4,122 | 499 | 71,129 | 0 %_10 % |
| Moderate | 60+ years | Tocantins | 18,193 | Inf | Inf | Inf | 246 | 36 | 8,007 | 112,056 | 15,104 | 4,155,174 | 2,275 | 326 | 75,893 | 0 %_10 % |
| High | 1–11 years | Alagoas | 260,778 | Inf | Inf | Inf | 13 | 3 | 124 | 8,943 | 2,043 | 81,963 | 83 | 19 | 761 | 0 %_50 % |
| High | 1–11 years | Ceará | 627,876 | Inf | Inf | Inf | 23 | 4 | 156 | 15,509 | 2,899 | 103,511 | 144 | 27 | 961 | 0 %_50 % |
| High | 1–11 years | Paraíba | 280,516 | Inf | Inf | Inf | 34 | 8 | 183 | 22,341 | 5,069 | 121,555 | 208 | 47 | 1,128 | 0 %_50 % |
| High | 1–11 years | Piauí | 239,967 | Inf | Inf | Inf | 19 | 4 | 144 | 12,361 | 2,829 | 95,298 | 115 | 26 | 885 | 0 %_50 % |
| High | 12–17 years | Alagoas | 153,825 | Inf | Inf | Inf | 16 | 3 | 166 | 26,810 | 5,809 | 286,988 | 166 | 36 | 1,781 | 0 %_50 % |
| High | 12–17 years | Ceará | 365,978 | Inf | Inf | Inf | 29 | 5 | 226 | 49,468 | 8,411 | 390,926 | 307 | 52 | 2,426 | 0 %_50 % |
| High | 12–17 years | Paraíba | 162,680 | Inf | Inf | Inf | 41 | 8 | 262 | 70,176 | 14,697 | 452,243 | 436 | 91 | 2,807 | 0 %_50 % |
| High | 12–17 years | Piauí | 146,345 | Inf | Inf | Inf | 23 | 5 | 216 | 39,637 | 8,420 | 373,828 | 246 | 52 | 2,320 | 0 %_50 % |
| High | 18–59 years | Alagoas | 997,514 | Inf | Inf | Inf | 23 | 4 | 349 | 56,761 | 10,417 | 887,751 | 269 | 49 | 4,190 | 0 %_50 % |
| High | 18–59 years | Ceará | 2,705,400 | Inf | Inf | Inf | 49 | 6 | 600 | 123,919 | 16,109 | 1,534,329 | 585 | 76 | 7,257 | 0 %_50 % |
| High | 18–59 years | Paraíba | 1,173,650 | Inf | Inf | Inf | 67 | 11 | 625 | 169,321 | 28,288 | 1,597,554 | 801 | 134 | 7,534 | 0 %_50 % |
| High | 18–59 years | Piauí | 1,010,615 | Inf | Inf | Inf | 39 | 7 | 603 | 98,006 | 16,399 | 1,536,603 | 464 | 78 | 7,299 | 0 %_50 % |
| High | 60+ years | Alagoas | 140,951 | Inf | Inf | Inf | 41 | 6 | 1,310 | 19,086 | 2,468 | 671,429 | 378 | 51 | 12,393 | 0 %_50 % |
| High | 60+ years | Ceará | 434,053 | Inf | Inf | Inf | 116 | 10 | 4,248 | 50,687 | 4,104 | 2,243,751 | 1,064 | 90 | 40,380 | 0 %_50 % |
| High | 60+ years | Paraíba | 203,370 | Inf | Inf | Inf | 146 | 17 | 3,135 | 63,550 | 6,683 | 1,564,848 | 1,339 | 150 | 29,508 | 0 %_50 % |
| High | 60+ years | Piauí | 169,081 | Inf | Inf | Inf | 90 | 10 | 4,341 | 40,778 | 4,254 | 2,256,454 | 836 | 92 | 41,150 | 0 %_50 % |
| Low | 1–11 years | Bahia | 961,065 | Inf | Inf | Inf | 391 | 87 | 1,392 | 259,152 | 57,682 | 922,716 | 2,411 | 537 | 8,573 | 0 %_50 % |
| Low | 1–11 years | Goiás | 505,801 | Inf | Inf | Inf | 413 | 133 | 1,552 | 273,769 | 88,296 | 1,029,261 | 2,544 | 821 | 9,556 | 0 %_50 % |
| Low | 1–11 years | Minas Gerais | 1,100,714 | Inf | Inf | Inf | 197 | 6 | 1,734 | 130,918 | 3,791 | 1,149,597 | 1,217 | 35 | 10,668 | 0 %_50 % |
| Low | 1–11 years | Rio Grande do Norte | 222,882 | Inf | Inf | Inf | 81 | 19 | 575 | 53,836 | 12,407 | 380,939 | 501 | 115 | 3,537 | 0 %_50 % |
| Low | 1–11 years | Sergipe | 174,400 | Inf | Inf | Inf | 65 | 14 | 284 | 43,098 | 9,537 | 188,579 | 401 | 89 | 1,753 | 0 %_50 % |
| Low | 12–17 years | Bahia | 575,979 | Inf | Inf | Inf | 455 | 94 | 1,910 | 787,133 | 162,966 | 3,303,341 | 4,885 | 1,011 | 20,501 | 0 %_50 % |
| Low | 12–17 years | Goiás | 280,863 | Inf | Inf | Inf | 472 | 143 | 2,053 | 815,919 | 246,474 | 3,550,003 | 5,064 | 1,530 | 22,031 | 0 %_50 % |
| Low | 12–17 years | Minas Gerais | 634,133 | Inf | Inf | Inf | 245 | 6 | 2,609 | 423,685 | 10,820 | 4,510,382 | 2,629 | 67 | 27,992 | 0 %_50 % |
| Low | 12–17 years | Rio Grande do Norte | 132,998 | Inf | Inf | Inf | 98 | 21 | 839 | 170,143 | 36,091 | 1,450,513 | 1,056 | 224 | 9,002 | 0 %_50 % |
| Low | 12–17 years | Sergipe | 104,548 | Inf | Inf | Inf | 75 | 16 | 358 | 129,994 | 27,437 | 619,428 | 807 | 170 | 3,844 | 0 %_50 % |
| Low | 18–59 years | Bahia | 4,209,700 | Inf | Inf | Inf | 694 | 118 | 4,388 | 1,747,791 | 294,710 | 11,162,008 | 8,263 | 1,399 | 52,740 | 0 %_50 % |
| Low | 18–59 years | Goiás | 2,233,990 | Inf | Inf | Inf | 671 | 173 | 4,370 | 1,692,047 | 432,857 | 11,178,657 | 7,988 | 2,051 | 52,569 | 0 %_50 % |
| Low | 18–59 years | Minas Gerais | 5,445,941 | Inf | Inf | Inf | 437 | 8 | 6,945 | 1,101,394 | 20,280 | 17,717,911 | 5,212 | 97 | 83,783 | 0 %_50 % |
| Low | 18–59 years | Rio Grande do Norte | 1,004,748 | Inf | Inf | Inf | 164 | 28 | 2,300 | 415,483 | 69,501 | 5,877,885 | 1,965 | 330 | 27,774 | 0 %_50 % |
| Low | 18–59 years | Sergipe | 731,513 | Inf | Inf | Inf | 111 | 20 | 678 | 280,409 | 51,358 | 1,729,569 | 1,323 | 243 | 8,146 | 0 %_50 % |
| Low | 60+ years | Bahia | 694,029 | Inf | Inf | Inf | 1,317 | 162 | 18,505 | 569,723 | 65,821 | 9,142,856 | 12,067 | 1,462 | 173,869 | 0 %_50 % |
| Low | 60+ years | Goiás | 310,050 | Inf | Inf | Inf | 1,142 | 224 | 17,141 | 537,079 | 100,840 | 9,310,813 | 10,634 | 2,069 | 163,719 | 0 %_50 % |
| Low | 60+ years | Minas Gerais | 1,027,451 | Inf | Inf | Inf | 1,071 | 12 | 36,918 | 502,928 | 5,002 | 19,447,752 | 9,967 | 107 | 350,815 | 0 %_50 % |
| Low | 60+ years | Rio Grande do Norte | 161,960 | Inf | Inf | Inf | 370 | 42 | 14,529 | 161,483 | 17,194 | 7,661,252 | 3,398 | 381 | 138,066 | 0 %_50 % |
| Low | 60+ years | Sergipe | 103,072 | Inf | Inf | Inf | 200 | 30 | 2,014 | 89,808 | 13,051 | 966,976 | 1,846 | 275 | 18,823 | 0 %_50 % |
| Moderate | 1–11 years | Pernambuco | 809,462 | Inf | Inf | Inf | 105 | 25 | 488 | 69,559 | 16,289 | 323,474 | 647 | 152 | 3,004 | 0 %_50 % |
| Moderate | 1–11 years | Tocantins | 148,881 | Inf | Inf | Inf | 64 | 16 | 406 | 42,330 | 10,819 | 268,844 | 394 | 101 | 2,496 | 0 %_50 % |
| Moderate | 12–17 years | Pernambuco | 470,483 | Inf | Inf | Inf | 124 | 27 | 676 | 213,715 | 46,161 | 1,168,829 | 1,326 | 286 | 7,254 | 0 %_50 % |
| Moderate | 12–17 years | Tocantins | 85,653 | Inf | Inf | Inf | 74 | 18 | 560 | 128,384 | 30,720 | 968,435 | 797 | 191 | 6,010 | 0 %_50 % |
| Moderate | 18–59 years | Pernambuco | 3,400,843 | Inf | Inf | Inf | 189 | 33 | 1,477 | 477,336 | 81,877 | 3,761,632 | 2,258 | 389 | 17,773 | 0 %_50 % |
| Moderate | 18–59 years | Tocantins | 552,313 | Inf | Inf | Inf | 110 | 23 | 1,256 | 277,238 | 56,705 | 3,214,897 | 1,308 | 268 | 15,156 | 0 %_50 % |
| Moderate | 60+ years | Pernambuco | 538,561 | Inf | Inf | Inf | 364 | 45 | 5,844 | 163,273 | 19,066 | 3,070,556 | 3,356 | 410 | 55,503 | 0 %_50 % |
| Moderate | 60+ years | Tocantins | 75,526 | Inf | Inf | Inf | 204 | 32 | 6,074 | 92,258 | 13,645 | 3,203,312 | 1,886 | 291 | 57,724 | 0 %_50 % |
| High | 1–11 years | Alagoas | 466,969 | Inf | Inf | Inf | 13 | 3 | 124 | 8,934 | 2,043 | 82,410 | 83 | 19 | 766 | 0 %_90 % |
| High | 1–11 years | Ceará | 1,171,973 | Inf | Inf | Inf | 24 | 5 | 163 | 16,081 | 3,008 | 108,220 | 149 | 28 | 1,004 | 0 %_90 % |
| High | 1–11 years | Paraíba | 533,528 | Inf | Inf | Inf | 35 | 8 | 191 | 23,514 | 5,196 | 126,302 | 219 | 48 | 1,172 | 0 %_90 % |
| High | 1–11 years | Piauí | 441,713 | Inf | Inf | Inf | 19 | 4 | 150 | 12,632 | 2,814 | 99,311 | 117 | 26 | 922 | 0 %_90 % |
| High | 12–17 years | Alagoas | 275,450 | Inf | Inf | Inf | 15 | 3 | 166 | 26,727 | 5,820 | 287,582 | 166 | 36 | 1,785 | 0 %_90 % |
| High | 12–17 years | Ceará | 683,123 | Inf | Inf | Inf | 30 | 5 | 234 | 51,339 | 8,752 | 404,522 | 319 | 54 | 2,510 | 0 %_90 % |
| High | 12–17 years | Paraíba | 309,409 | Inf | Inf | Inf | 43 | 9 | 270 | 73,787 | 15,080 | 466,312 | 458 | 94 | 2,894 | 0 %_90 % |
| High | 12–17 years | Piauí | 269,381 | Inf | Inf | Inf | 23 | 5 | 224 | 40,307 | 8,354 | 387,360 | 250 | 52 | 2,404 | 0 %_90 % |
| High | 18–59 years | Alagoas | 1,786,221 | Inf | Inf | Inf | 22 | 4 | 348 | 56,126 | 10,572 | 885,132 | 266 | 50 | 4,180 | 0 %_90 % |
| High | 18–59 years | Ceará | 5,049,814 | Inf | Inf | Inf | 50 | 7 | 612 | 127,664 | 16,920 | 1,570,832 | 603 | 80 | 7,416 | 0 %_90 % |
| High | 18–59 years | Paraíba | 2,232,224 | Inf | Inf | Inf | 70 | 12 | 649 | 176,936 | 29,159 | 1,656,597 | 837 | 138 | 7,818 | 0 %_90 % |
| High | 18–59 years | Piauí | 1,860,260 | Inf | Inf | Inf | 39 | 7 | 616 | 99,413 | 16,610 | 1,572,089 | 470 | 79 | 7,450 | 0 %_90 % |
| High | 60+ years | Alagoas | 252,398 | Inf | Inf | Inf | 40 | 6 | 1,287 | 18,632 | 2,567 | 666,562 | 371 | 53 | 12,191 | 0 %_90 % |
| High | 60+ years | Ceará | 810,190 | Inf | Inf | Inf | 119 | 10 | 4,313 | 51,979 | 4,264 | 2,268,768 | 1,092 | 94 | 40,975 | 0 %_90 % |
| High | 60+ years | Paraíba | 386,799 | Inf | Inf | Inf | 152 | 17 | 3,244 | 65,713 | 7,031 | 1,608,538 | 1,393 | 157 | 30,496 | 0 %_90 % |
| High | 60+ years | Piauí | 311,230 | Inf | Inf | Inf | 91 | 10 | 4,398 | 40,886 | 4,384 | 2,321,467 | 840 | 95 | 41,806 | 0 %_90 % |
| Low | 1–11 years | Bahia | 1,836,096 | Inf | Inf | Inf | 415 | 93 | 1,477 | 275,048 | 61,949 | 979,126 | 2,558 | 577 | 9,097 | 0 %_90 % |
| Low | 1–11 years | Goiás | 940,954 | Inf | Inf | Inf | 426 | 137 | 1,611 | 282,118 | 90,908 | 1,068,190 | 2,622 | 845 | 9,918 | 0 %_90 % |
| Low | 1–11 years | Minas Gerais | 2,321,234 | Inf | Inf | Inf | 232 | 7 | 1,997 | 153,838 | 4,372 | 1,323,784 | 1,430 | 41 | 12,284 | 0 %_90 % |
| Low | 1–11 years | Rio Grande do Norte | 422,092 | Inf | Inf | Inf | 86 | 20 | 598 | 57,011 | 13,031 | 396,792 | 530 | 121 | 3,684 | 0 %_90 % |
| Low | 1–11 years | Sergipe | 309,353 | Inf | Inf | Inf | 64 | 14 | 283 | 42,397 | 9,490 | 187,477 | 394 | 88 | 1,743 | 0 %_90 % |
| Low | 12–17 years | Bahia | 1,100,397 | Inf | Inf | Inf | 483 | 101 | 2,021 | 835,272 | 175,376 | 3,494,954 | 5,184 | 1,088 | 21,690 | 0 %_90 % |
| Low | 12–17 years | Goiás | 522,497 | Inf | Inf | Inf | 486 | 147 | 2,117 | 840,251 | 254,915 | 3,660,206 | 5,215 | 1,582 | 22,715 | 0 %_90 % |
| Low | 12–17 years | Minas Gerais | 1,337,288 | Inf | Inf | Inf | 288 | 7 | 2,995 | 497,192 | 12,513 | 5,178,153 | 3,086 | 78 | 32,136 | 0 %_90 % |
| Low | 12–17 years | Rio Grande do Norte | 251,870 | Inf | Inf | Inf | 104 | 22 | 876 | 180,168 | 37,974 | 1,514,827 | 1,118 | 236 | 9,401 | 0 %_90 % |
| Low | 12–17 years | Sergipe | 185,449 | Inf | Inf | Inf | 74 | 16 | 355 | 127,883 | 27,285 | 613,904 | 794 | 169 | 3,810 | 0 %_90 % |
| Low | 18–59 years | Bahia | 8,042,554 | Inf | Inf | Inf | 734 | 127 | 4,645 | 1,846,643 | 316,407 | 11,825,395 | 8,730 | 1,502 | 55,859 | 0 %_90 % |
| Low | 18–59 years | Goiás | 4,155,940 | Inf | Inf | Inf | 691 | 180 | 4,477 | 1,743,206 | 450,505 | 11,447,128 | 8,231 | 2,135 | 53,867 | 0 %_90 % |
| Low | 18–59 years | Minas Gerais | 11,484,644 | Inf | Inf | Inf | 512 | 9 | 8,023 | 1,289,369 | 23,028 | 20,473,067 | 6,107 | 110 | 96,789 | 0 %_90 % |
| Low | 18–59 years | Rio Grande do Norte | 1,902,786 | Inf | Inf | Inf | 174 | 29 | 2,388 | 438,957 | 73,810 | 6,100,999 | 2,076 | 350 | 28,844 | 0 %_90 % |
| Low | 18–59 years | Sergipe | 1,297,570 | Inf | Inf | Inf | 109 | 20 | 662 | 275,288 | 51,382 | 1,689,306 | 1,299 | 243 | 7,950 | 0 %_90 % |
| Low | 60+ years | Bahia | 1,325,930 | Inf | Inf | Inf | 1,391 | 173 | 19,694 | 601,542 | 70,846 | 9,665,966 | 12,740 | 1,571 | 184,824 | 0 %_90 % |
| Low | 60+ years | Goiás | 576,792 | Inf | Inf | Inf | 1,169 | 235 | 17,198 | 549,585 | 105,692 | 9,398,181 | 10,883 | 2,168 | 164,428 | 0 %_90 % |
| Low | 60+ years | Minas Gerais | 2,166,736 | Inf | Inf | Inf | 1,233 | 13 | 43,154 | 580,725 | 5,599 | 22,478,748 | 11,481 | 120 | 409,278 | 0 %_90 % |
| Low | 60+ years | Rio Grande do Norte | 306,719 | Inf | Inf | Inf | 391 | 45 | 14,980 | 170,420 | 18,393 | 7,719,354 | 3,585 | 408 | 141,796 | 0 %_90 % |
| Low | 60+ years | Sergipe | 182,831 | Inf | Inf | Inf | 198 | 30 | 1,997 | 88,702 | 13,053 | 958,885 | 1,823 | 276 | 18,663 | 0 %_90 % |
| Moderate | 1–11 years | Pernambuco | 1,306,851 | Inf | Inf | Inf | 94 | 22 | 448 | 62,193 | 14,735 | 296,828 | 578 | 137 | 2,757 | 0 %_90 % |
| Moderate | 1–11 years | Tocantins | 241,606 | Inf | Inf | Inf | 58 | 15 | 359 | 38,151 | 9,966 | 238,119 | 355 | 93 | 2,211 | 0 %_90 % |
| Moderate | 12–17 years | Pernambuco | 759,581 | Inf | Inf | Inf | 110 | 24 | 615 | 190,773 | 41,825 | 1,063,930 | 1,184 | 260 | 6,603 | 0 %_90 % |
| Moderate | 12–17 years | Tocantins | 138,998 | Inf | Inf | Inf | 67 | 16 | 496 | 115,629 | 28,343 | 857,957 | 718 | 176 | 5,325 | 0 %_90 % |
| Moderate | 18–59 years | Pernambuco | 5,490,560 | Inf | Inf | Inf | 169 | 30 | 1,354 | 425,786 | 75,060 | 3,446,870 | 2,014 | 356 | 16,275 | 0 %_90 % |
| Moderate | 18–59 years | Tocantins | 896,301 | Inf | Inf | Inf | 98 | 21 | 1,106 | 248,900 | 52,320 | 2,828,622 | 1,174 | 247 | 13,338 | 0 %_90 % |
| Moderate | 60+ years | Pernambuco | 869,490 | Inf | Inf | Inf | 324 | 41 | 5,260 | 145,221 | 17,451 | 2,710,069 | 2,990 | 376 | 49,791 | 0 %_90 % |
| Moderate | 60+ years | Tocantins | 122,565 | Inf | Inf | Inf | 182 | 29 | 5,253 | 82,486 | 12,613 | 2,788,471 | 1,686 | 268 | 49,977 | 0 %_90 % |
| High | 1–11 years | Alagoas | 52,717 | 1 | 0 | 12 | 2 | 1 | 20 | 2,167 | 529 | 20,101 | 18 | 4 | 163 | 98·9 %_10 % |
| High | 1–11 years | Ceará | 115,930 | 3 | 1 | 27 | 5 | 1 | 39 | 4,521 | 817 | 34,470 | 38 | 7 | 292 | 98·9 %_10 % |
| High | 1–11 years | Paraíba | 49,504 | 4 | 1 | 31 | 7 | 1 | 47 | 6,246 | 1,388 | 40,791 | 53 | 12 | 350 | 98·9 %_10 % |
| High | 1–11 years | Piauí | 45,738 | 3 | 1 | 25 | 4 | 1 | 36 | 3,804 | 891 | 30,924 | 32 | 8 | 265 | 98·9 %_10 % |
| High | 12–17 years | Alagoas | 31,096 | 1 | 0 | 14 | 2 | 1 | 23 | 3,095 | 750 | 31,582 | 22 | 5 | 223 | 98·9 %_10 % |
| High | 12–17 years | Ceará | 67,573 | 3 | 1 | 32 | 5 | 1 | 49 | 7,628 | 1,295 | 70,547 | 54 | 9 | 482 | 98·9 %_10 % |
| High | 12–17 years | Paraíba | 28,709 | 5 | 1 | 37 | 7 | 2 | 58 | 10,404 | 2,090 | 82,569 | 75 | 15 | 574 | 98·9 %_10 % |
| High | 12–17 years | Piauí | 27,894 | 3 | 1 | 30 | 5 | 1 | 46 | 6,522 | 1,464 | 65,378 | 46 | 11 | 453 | 98·9 %_10 % |
| High | 18–59 years | Alagoas | 201,650 | 2 | 0 | 23 | 3 | 1 | 39 | 4,456 | 1,020 | 56,511 | 31 | 7 | 387 | 98·9 %_10 % |
| High | 18–59 years | Ceará | 499,520 | 5 | 1 | 57 | 8 | 1 | 95 | 12,483 | 1,803 | 151,777 | 84 | 12 | 971 | 98·9 %_10 % |
| High | 18–59 years | Paraíba | 207,118 | 7 | 1 | 63 | 11 | 2 | 104 | 16,590 | 2,989 | 167,654 | 114 | 21 | 1,079 | 98·9 %_10 % |
| High | 18–59 years | Piauí | 192,626 | 4 | 1 | 54 | 7 | 1 | 88 | 10,425 | 2,053 | 137,759 | 70 | 14 | 898 | 98·9 %_10 % |
| High | 60+ years | Alagoas | 28,494 | 2 | 0 | 38 | 4 | 1 | 69 | 3,425 | 604 | 64,092 | 36 | 7 | 642 | 98·9 %_10 % |
| High | 60+ years | Ceará | 80,143 | 7 | 1 | 132 | 12 | 1 | 236 | 8,993 | 978 | 182,549 | 113 | 13 | 2,171 | 98·9 %_10 % |
| High | 60+ years | Paraíba | 35,889 | 9 | 1 | 133 | 15 | 2 | 233 | 11,461 | 1,607 | 170,119 | 146 | 21 | 2,158 | 98·9 %_10 % |
| High | 60+ years | Piauí | 32,227 | 6 | 1 | 127 | 10 | 2 | 233 | 7,541 | 1,109 | 175,488 | 96 | 15 | 2,122 | 98·9 %_10 % |
| Low | 1–11 years | Bahia | 167,710 | 54 | 6 | 361 | 83 | 10 | 441 | 75,717 | 10,800 | 354,057 | 648 | 90 | 3,108 | 98·9 %_10 % |
| Low | 1–11 years | Goiás | 94,119 | 81 | 20 | 448 | 113 | 28 | 559 | 100,039 | 26,833 | 449,922 | 858 | 225 | 3,931 | 98·9 %_10 % |
| Low | 1–11 years | Minas Gerais | 141,693 | 10 | 0 | 225 | 16 | 0 | 352 | 16,964 | 365 | 296,227 | 138 | 3 | 2,559 | 98·9 %_10 % |
| Low | 1–11 years | Rio Grande do Norte | 39,752 | 9 | 2 | 102 | 14 | 3 | 147 | 13,605 | 2,756 | 126,948 | 114 | 23 | 1,085 | 98·9 %_10 % |
| Low | 1–11 years | Sergipe | 35,934 | 8 | 2 | 49 | 13 | 2 | 68 | 12,415 | 2,456 | 63,088 | 103 | 20 | 530 | 98·9 %_10 % |
| Low | 12–17 years | Bahia | 100,511 | 57 | 7 | 426 | 88 | 11 | 544 | 125,417 | 13,975 | 826,624 | 893 | 106 | 5,534 | 98·9 %_10 % |
| Low | 12–17 years | Goiás | 52,263 | 86 | 20 | 521 | 121 | 29 | 662 | 181,033 | 41,634 | 1,034,722 | 1,242 | 292 | 6,792 | 98·9 %_10 % |
| Low | 12–17 years | Minas Gerais | 81,631 | 11 | 0 | 265 | 17 | 0 | 457 | 22,874 | 447 | 675,885 | 169 | 3 | 4,597 | 98·9 %_10 % |
| Low | 12–17 years | Rio Grande do Norte | 23,721 | 10 | 2 | 122 | 15 | 3 | 182 | 21,363 | 3,886 | 262,466 | 154 | 29 | 1,801 | 98·9 %_10 % |
| Low | 12–17 years | Sergipe | 21,541 | 8 | 2 | 55 | 13 | 2 | 77 | 18,766 | 3,451 | 110,671 | 134 | 25 | 770 | 98·9 %_10 % |
| Low | 18–59 years | Bahia | 734,611 | 79 | 9 | 695 | 130 | 15 | 951 | 201,540 | 21,134 | 1,677,730 | 1,355 | 156 | 10,139 | 98·9 %_10 % |
| Low | 18–59 years | Goiás | 415,700 | 110 | 26 | 817 | 164 | 37 | 1,155 | 278,183 | 59,315 | 2,119,463 | 1,751 | 393 | 12,462 | 98·9 %_10 % |
| Low | 18–59 years | Minas Gerais | 701,046 | 18 | 0 | 508 | 31 | 0 | 885 | 42,263 | 608 | 1,480,682 | 305 | 5 | 9,315 | 98·9 %_10 % |
| Low | 18–59 years | Rio Grande do Norte | 179,202 | 14 | 2 | 223 | 24 | 4 | 362 | 35,174 | 5,698 | 587,035 | 242 | 41 | 3,733 | 98·9 %_10 % |
| Low | 18–59 years | Sergipe | 150,723 | 11 | 2 | 83 | 19 | 3 | 120 | 28,518 | 4,933 | 188,913 | 195 | 34 | 1,238 | 98·9 %_10 % |
| Low | 60+ years | Bahia | 121,111 | 98 | 9 | 1,324 | 164 | 14 | 2,153 | 121,439 | 12,158 | 1,412,043 | 1,557 | 136 | 19,971 | 98·9 %_10 % |
| Low | 60+ years | Goiás | 57,694 | 133 | 25 | 1,533 | 206 | 37 | 2,481 | 147,642 | 28,269 | 1,766,183 | 1,968 | 354 | 23,471 | 98·9 %_10 % |
| Low | 60+ years | Minas Gerais | 132,262 | 22 | 0 | 1,092 | 38 | 0 | 2,170 | 36,448 | 473 | 1,555,901 | 361 | 5 | 20,335 | 98·9 %_10 % |
| Low | 60+ years | Rio Grande do Norte | 28,886 | 19 | 2 | 501 | 32 | 4 | 932 | 25,768 | 3,403 | 709,324 | 306 | 41 | 8,585 | 98·9 %_10 % |
| Low | 60+ years | Sergipe | 21,237 | 14 | 2 | 132 | 23 | 3 | 206 | 19,192 | 3,012 | 161,174 | 222 | 34 | 1,927 | 98·9 %_10 % |
| Moderate | 1–11 years | Pernambuco | 196,549 | 21 | 4 | 128 | 32 | 6 | 175 | 29,067 | 5,984 | 147,558 | 249 | 51 | 1,274 | 98·9 %_10 % |
| Moderate | 1–11 years | Tocantins | 35,864 | 10 | 2 | 89 | 16 | 4 | 133 | 15,448 | 3,509 | 114,669 | 129 | 29 | 980 | 98·9 %_10 % |
| Moderate | 12–17 years | Pernambuco | 114,240 | 22 | 4 | 150 | 35 | 7 | 208 | 50,312 | 9,130 | 306,260 | 354 | 66 | 2,087 | 98·9 %_10 % |
| Moderate | 12–17 years | Tocantins | 20,633 | 11 | 2 | 105 | 17 | 4 | 158 | 24,466 | 5,061 | 219,803 | 173 | 37 | 1,535 | 98·9 %_10 % |
| Moderate | 18–59 years | Pernambuco | 825,774 | 30 | 6 | 245 | 49 | 8 | 366 | 78,011 | 12,881 | 611,058 | 514 | 88 | 3,825 | 98·9 %_10 % |
| Moderate | 18–59 years | Tocantins | 133,047 | 14 | 3 | 173 | 24 | 5 | 269 | 36,948 | 7,150 | 421,404 | 249 | 50 | 2,732 | 98·9 %_10 % |
| Moderate | 60+ years | Pernambuco | 130,770 | 38 | 6 | 483 | 66 | 9 | 757 | 48,112 | 6,575 | 560,498 | 625 | 84 | 7,063 | 98·9 %_10 % |
| Moderate | 60+ years | Tocantins | 18,193 | 18 | 3 | 345 | 31 | 5 | 595 | 24,274 | 3,972 | 455,746 | 290 | 48 | 5,412 | 98·9 %_10 % |
| High | 1–11 years | Alagoas | 260,778 | 2 | 0 | 17 | 3 | 1 | 29 | 3,149 | 754 | 31,029 | 25 | 6 | 247 | 98·9 %_50 % |
| High | 1–11 years | Ceará | 627,876 | 4 | 1 | 34 | 7 | 1 | 56 | 6,781 | 1,218 | 51,442 | 57 | 10 | 431 | 98·9 %_50 % |
| High | 1–11 years | Paraíba | 280,516 | 6 | 1 | 42 | 10 | 2 | 67 | 9,684 | 2,012 | 59,887 | 82 | 17 | 508 | 98·9 %_50 % |
| High | 1–11 years | Piauí | 239,967 | 3 | 1 | 32 | 6 | 1 | 51 | 5,435 | 1,205 | 45,971 | 46 | 10 | 387 | 98·9 %_50 % |
| High | 12–17 years | Alagoas | 153,825 | 2 | 0 | 17 | 3 | 1 | 30 | 3,801 | 933 | 40,682 | 28 | 7 | 290 | 98·9 %_50 % |
| High | 12–17 years | Ceará | 365,978 | 4 | 1 | 39 | 7 | 1 | 65 | 10,138 | 1,723 | 92,824 | 73 | 13 | 642 | 98·9 %_50 % |
| High | 12–17 years | Paraíba | 162,680 | 6 | 1 | 47 | 10 | 2 | 77 | 14,197 | 2,754 | 107,761 | 103 | 21 | 755 | 98·9 %_50 % |
| High | 12–17 years | Piauí | 146,345 | 4 | 1 | 37 | 6 | 1 | 60 | 8,198 | 1,779 | 83,853 | 59 | 13 | 587 | 98·9 %_50 % |
| High | 18–59 years | Alagoas | 997,514 | 3 | 1 | 40 | 6 | 1 | 71 | 8,002 | 1,703 | 96,783 | 58 | 13 | 685 | 98·9 %_50 % |
| High | 18–59 years | Ceará | 2,705,400 | 8 | 1 | 81 | 14 | 2 | 148 | 20,600 | 3,073 | 226,567 | 144 | 22 | 1,494 | 98·9 %_50 % |
| High | 18–59 years | Paraíba | 1,173,650 | 11 | 2 | 95 | 20 | 4 | 163 | 28,641 | 5,039 | 248,904 | 202 | 37 | 1,651 | 98·9 %_50 % |
| High | 18–59 years | Piauí | 1,010,615 | 6 | 1 | 78 | 11 | 2 | 133 | 16,183 | 3,085 | 198,528 | 113 | 22 | 1,326 | 98·9 %_50 % |
| High | 60+ years | Alagoas | 140,951 | 3 | 1 | 45 | 4 | 1 | 81 | 4,168 | 793 | 75,801 | 42 | 9 | 748 | 98·9 %_50 % |
| High | 60+ years | Ceará | 434,053 | 8 | 1 | 152 | 15 | 2 | 286 | 11,809 | 1,394 | 220,288 | 143 | 18 | 2,632 | 98·9 %_50 % |
| High | 60+ years | Paraíba | 203,370 | 11 | 2 | 159 | 20 | 3 | 283 | 15,704 | 2,269 | 210,348 | 192 | 28 | 2,620 | 98·9 %_50 % |
| High | 60+ years | Piauí | 169,081 | 7 | 1 | 149 | 12 | 2 | 275 | 9,539 | 1,465 | 211,306 | 116 | 19 | 2,500 | 98·9 %_50 % |
| Low | 1–11 years | Bahia | 961,065 | 78 | 10 | 478 | 128 | 18 | 643 | 119,942 | 19,005 | 527,541 | 1,019 | 158 | 4,601 | 98·9 %_50 % |
| Low | 1–11 years | Goiás | 505,801 | 104 | 26 | 557 | 157 | 40 | 764 | 143,018 | 39,790 | 626,739 | 1,214 | 332 | 5,446 | 98·9 %_50 % |
| Low | 1–11 years | Minas Gerais | 1,100,714 | 22 | 0 | 415 | 39 | 1 | 680 | 42,215 | 836 | 590,501 | 340 | 7 | 5,055 | 98·9 %_50 % |
| Low | 1–11 years | Rio Grande do Norte | 222,882 | 13 | 2 | 136 | 22 | 4 | 214 | 21,992 | 4,397 | 191,069 | 182 | 37 | 1,613 | 98·9 %_50 % |
| Low | 1–11 years | Sergipe | 174,400 | 10 | 2 | 59 | 17 | 3 | 91 | 17,221 | 3,521 | 87,943 | 141 | 29 | 727 | 98·9 %_50 % |
| Low | 12–17 years | Bahia | 575,979 | 78 | 10 | 540 | 129 | 16 | 747 | 179,729 | 21,510 | 1,113,494 | 1,293 | 164 | 7,542 | 98·9 %_50 % |
| Low | 12–17 years | Goiás | 280,863 | 105 | 25 | 625 | 161 | 39 | 878 | 236,445 | 55,528 | 1,350,339 | 1,634 | 394 | 8,958 | 98·9 %_50 % |
| Low | 12–17 years | Minas Gerais | 634,133 | 21 | 0 | 471 | 36 | 1 | 807 | 47,923 | 831 | 1,170,358 | 356 | 7 | 8,057 | 98·9 %_50 % |
| Low | 12–17 years | Rio Grande do Norte | 132,998 | 13 | 2 | 155 | 22 | 4 | 250 | 30,530 | 5,453 | 355,839 | 221 | 41 | 2,459 | 98·9 %_50 % |
| Low | 12–17 years | Sergipe | 104,548 | 10 | 2 | 62 | 17 | 3 | 96 | 22,925 | 4,341 | 135,716 | 165 | 32 | 952 | 98·9 %_50 % |
| Low | 18–59 years | Bahia | 4,209,700 | 148 | 23 | 1,008 | 249 | 40 | 1,477 | 380,263 | 53,543 | 2,538,818 | 2,579 | 399 | 15,639 | 98·9 %_50 % |
| Low | 18–59 years | Goiás | 2,233,990 | 174 | 45 | 1,121 | 274 | 70 | 1,679 | 449,988 | 108,396 | 3,001,462 | 2,891 | 734 | 17,959 | 98·9 %_50 % |
| Low | 18–59 years | Minas Gerais | 5,445,941 | 62 | 1 | 1,002 | 110 | 2 | 1,824 | 150,906 | 2,673 | 2,927,176 | 1,097 | 21 | 18,851 | 98·9 %_50 % |
| Low | 18–59 years | Rio Grande do Norte | 1,004,748 | 27 | 5 | 331 | 47 | 9 | 575 | 67,620 | 11,751 | 897,658 | 478 | 87 | 5,844 | 98·9 %_50 % |
| Low | 18–59 years | Sergipe | 731,513 | 19 | 4 | 120 | 34 | 7 | 197 | 47,982 | 9,034 | 295,920 | 338 | 65 | 1,988 | 98·9 %_50 % |
| Low | 60+ years | Bahia | 694,029 | 129 | 12 | 1,643 | 227 | 21 | 2,745 | 172,010 | 19,411 | 1,808,565 | 2,159 | 207 | 25,342 | 98·9 %_50 % |
| Low | 60+ years | Goiás | 310,050 | 160 | 32 | 1,796 | 264 | 49 | 2,972 | 195,881 | 39,829 | 2,100,588 | 2,522 | 476 | 28,026 | 98·9 %_50 % |
| Low | 60+ years | Minas Gerais | 1,027,451 | 41 | 1 | 1,769 | 75 | 1 | 3,550 | 74,457 | 963 | 2,624,875 | 723 | 9 | 33,210 | 98·9 %_50 % |
| Low | 60+ years | Rio Grande do Norte | 161,960 | 25 | 3 | 612 | 44 | 6 | 1,130 | 36,462 | 5,058 | 858,472 | 419 | 57 | 10,435 | 98·9 %_50 % |
| Low | 60+ years | Sergipe | 103,072 | 15 | 3 | 143 | 27 | 4 | 234 | 23,505 | 3,840 | 190,550 | 260 | 41 | 2,195 | 98·9 %_50 % |
| Moderate | 1–11 years | Pernambuco | 809,462 | 21 | 4 | 124 | 35 | 7 | 196 | 32,463 | 6,951 | 171,880 | 274 | 59 | 1,467 | 98·9 %_50 % |
| Moderate | 1–11 years | Tocantins | 148,881 | 11 | 3 | 87 | 18 | 4 | 142 | 18,183 | 4,338 | 129,742 | 150 | 36 | 1,088 | 98·9 %_50 % |
| Moderate | 12–17 years | Pernambuco | 470,483 | 21 | 4 | 138 | 36 | 7 | 222 | 49,963 | 9,518 | 319,742 | 356 | 70 | 2,209 | 98·9 %_50 % |
| Moderate | 12–17 years | Tocantins | 85,653 | 11 | 2 | 95 | 18 | 4 | 159 | 25,207 | 5,479 | 219,993 | 180 | 40 | 1,542 | 98·9 %_50 % |
| Moderate | 18–59 years | Pernambuco | 3,400,843 | 37 | 7 | 261 | 64 | 12 | 445 | 96,066 | 17,603 | 707,179 | 656 | 125 | 4,574 | 98·9 %_50 % |
| Moderate | 18–59 years | Tocantins | 552,313 | 19 | 4 | 187 | 34 | 8 | 320 | 49,032 | 10,653 | 482,181 | 341 | 76 | 3,198 | 98·9 %_50 % |
| Moderate | 60+ years | Pernambuco | 538,561 | 36 | 6 | 427 | 64 | 9 | 716 | 48,223 | 7,144 | 525,190 | 604 | 88 | 6,662 | 98·9 %_50 % |
| Moderate | 60+ years | Tocantins | 75,526 | 17 | 3 | 306 | 30 | 5 | 530 | 24,850 | 4,463 | 414,001 | 286 | 51 | 4,832 | 98·9 %_50 % |
| High | 1–11 years | Alagoas | 466,969 | 2 | 1 | 22 | 4 | 1 | 40 | 4,311 | 1,004 | 43,553 | 34 | 8 | 341 | 98·9 %_90 % |
| High | 1–11 years | Ceará | 1,171,973 | 5 | 1 | 42 | 9 | 2 | 74 | 9,276 | 1,656 | 70,188 | 77 | 14 | 581 | 98·9 %_90 % |
| High | 1–11 years | Paraíba | 533,528 | 8 | 2 | 53 | 14 | 3 | 88 | 13,476 | 2,739 | 81,868 | 113 | 23 | 687 | 98·9 %_90 % |
| High | 1–11 years | Piauí | 441,713 | 4 | 1 | 40 | 7 | 2 | 67 | 7,309 | 1,587 | 62,921 | 60 | 13 | 521 | 98·9 %_90 % |
| High | 12–17 years | Alagoas | 275,450 | 2 | 1 | 21 | 3 | 1 | 38 | 4,627 | 1,128 | 50,283 | 34 | 8 | 361 | 98·9 %_90 % |
| High | 12–17 years | Ceará | 683,123 | 5 | 1 | 45 | 9 | 2 | 81 | 12,549 | 2,139 | 113,802 | 91 | 16 | 795 | 98·9 %_90 % |
| High | 12–17 years | Paraíba | 309,409 | 7 | 1 | 56 | 13 | 3 | 96 | 17,881 | 3,409 | 132,764 | 132 | 26 | 938 | 98·9 %_90 % |
| High | 12–17 years | Piauí | 269,381 | 4 | 1 | 44 | 7 | 2 | 74 | 9,950 | 2,136 | 101,845 | 72 | 16 | 719 | 98·9 %_90 % |
| High | 18–59 years | Alagoas | 1,786,221 | 5 | 1 | 59 | 9 | 2 | 108 | 12,366 | 2,588 | 142,273 | 91 | 19 | 1,023 | 98·9 %_90 % |
| High | 18–59 years | Ceará | 5,049,814 | 11 | 2 | 108 | 21 | 3 | 204 | 29,666 | 4,509 | 302,767 | 212 | 34 | 2,032 | 98·9 %_90 % |
| High | 18–59 years | Paraíba | 2,232,224 | 17 | 3 | 130 | 31 | 6 | 229 | 42,190 | 7,540 | 339,616 | 305 | 57 | 2,296 | 98·9 %_90 % |
| High | 18–59 years | Piauí | 1,860,260 | 9 | 2 | 104 | 16 | 3 | 183 | 22,644 | 4,306 | 262,592 | 163 | 32 | 1,792 | 98·9 %_90 % |
| High | 60+ years | Alagoas | 252,398 | 3 | 1 | 51 | 5 | 1 | 93 | 5,078 | 993 | 89,073 | 50 | 10 | 863 | 98·9 %_90 % |
| High | 60+ years | Ceará | 810,190 | 10 | 1 | 170 | 18 | 2 | 332 | 14,710 | 1,807 | 258,459 | 173 | 22 | 3,054 | 98·9 %_90 % |
| High | 60+ years | Paraíba | 386,799 | 14 | 2 | 183 | 25 | 4 | 330 | 19,975 | 2,947 | 249,978 | 237 | 35 | 3,054 | 98·9 %_90 % |
| High | 60+ years | Piauí | 311,230 | 8 | 1 | 169 | 14 | 2 | 313 | 11,614 | 1,837 | 245,162 | 135 | 23 | 2,842 | 98·9 %_90 % |
| Low | 1–11 years | Bahia | 1,836,096 | 101 | 14 | 574 | 173 | 26 | 830 | 165,648 | 27,841 | 695,815 | 1,399 | 230 | 6,029 | 98·9 %_90 % |
| Low | 1–11 years | Goiás | 940,954 | 126 | 33 | 653 | 203 | 53 | 970 | 189,431 | 53,594 | 814,339 | 1,595 | 443 | 7,022 | 98·9 %_90 % |
| Low | 1–11 years | Minas Gerais | 2,321,234 | 35 | 1 | 553 | 63 | 1 | 974 | 70,028 | 1,390 | 873,898 | 561 | 11 | 7,399 | 98·9 %_90 % |
| Low | 1–11 years | Rio Grande do Norte | 422,092 | 17 | 3 | 167 | 30 | 6 | 283 | 30,976 | 6,165 | 259,872 | 254 | 51 | 2,173 | 98·9 %_90 % |
| Low | 1–11 years | Sergipe | 309,353 | 12 | 2 | 70 | 21 | 4 | 116 | 22,793 | 4,692 | 116,147 | 184 | 38 | 948 | 98·9 %_90 % |
| Low | 12–17 years | Bahia | 1,100,397 | 96 | 13 | 631 | 167 | 22 | 935 | 231,333 | 28,883 | 1,375,872 | 1,676 | 221 | 9,392 | 98·9 %_90 % |
| Low | 12–17 years | Goiás | 522,497 | 122 | 30 | 712 | 200 | 49 | 1,079 | 291,503 | 69,313 | 1,639,921 | 2,027 | 496 | 10,948 | 98·9 %_90 % |
| Low | 12–17 years | Minas Gerais | 1,337,288 | 30 | 1 | 607 | 54 | 1 | 1,104 | 71,637 | 1,216 | 1,576,071 | 535 | 10 | 10,952 | 98·9 %_90 % |
| Low | 12–17 years | Rio Grande do Norte | 251,870 | 16 | 3 | 184 | 29 | 5 | 314 | 39,191 | 6,984 | 441,956 | 286 | 53 | 3,074 | 98·9 %_90 % |
| Low | 12–17 years | Sergipe | 185,449 | 11 | 2 | 71 | 20 | 4 | 117 | 27,524 | 5,275 | 162,439 | 199 | 39 | 1,148 | 98·9 %_90 % |
| Low | 18–59 years | Bahia | 8,042,554 | 213 | 37 | 1,281 | 370 | 66 | 1,977 | 556,173 | 89,190 | 3,328,856 | 3,814 | 670 | 20,785 | 98·9 %_90 % |
| Low | 18–59 years | Goiás | 4,155,940 | 238 | 65 | 1,399 | 390 | 106 | 2,197 | 626,608 | 159,471 | 3,847,545 | 4,089 | 1,095 | 23,326 | 98·9 %_90 % |
| Low | 18–59 years | Minas Gerais | 11,484,644 | 112 | 2 | 1,447 | 203 | 4 | 2,722 | 276,187 | 5,489 | 4,231,104 | 2,019 | 44 | 27,778 | 98·9 %_90 % |
| Low | 18–59 years | Rio Grande do Norte | 1,902,786 | 41 | 8 | 441 | 74 | 14 | 797 | 103,172 | 18,742 | 1,212,793 | 742 | 142 | 8,023 | 98·9 %_90 % |
| Low | 18–59 years | Sergipe | 1,297,570 | 28 | 6 | 163 | 50 | 10 | 277 | 69,929 | 13,651 | 407,017 | 500 | 100 | 2,775 | 98·9 %_90 % |
| Low | 60+ years | Bahia | 1,325,930 | 156 | 16 | 1,894 | 285 | 29 | 3,227 | 220,728 | 26,827 | 2,148,649 | 2,711 | 278 | 29,775 | 98·9 %_90 % |
| Low | 60+ years | Goiás | 576,792 | 185 | 38 | 2,020 | 319 | 61 | 3,418 | 244,095 | 51,543 | 2,426,896 | 3,054 | 595 | 32,133 | 98·9 %_90 % |
| Low | 60+ years | Minas Gerais | 2,166,736 | 59 | 1 | 2,213 | 109 | 1 | 4,489 | 109,697 | 1,516 | 3,391,834 | 1,047 | 14 | 42,005 | 98·9 %_90 % |
| Low | 60+ years | Rio Grande do Norte | 306,719 | 30 | 4 | 704 | 55 | 8 | 1,308 | 46,674 | 6,711 | 998,955 | 521 | 73 | 12,087 | 98·9 %_90 % |
| Low | 60+ years | Sergipe | 182,831 | 18 | 3 | 157 | 32 | 5 | 267 | 28,394 | 4,788 | 222,626 | 304 | 50 | 2,503 | 98·9 %_90 % |
| Moderate | 1–11 years | Pernambuco | 1,306,851 | 22 | 5 | 130 | 39 | 8 | 222 | 37,976 | 8,197 | 201,436 | 317 | 68 | 1,700 | 98·9 %_90 % |
| Moderate | 1–11 years | Tocantins | 241,606 | 12 | 3 | 93 | 21 | 5 | 162 | 21,907 | 5,291 | 153,382 | 179 | 43 | 1,267 | 98·9 %_90 % |
| Moderate | 12–17 years | Pernambuco | 759,581 | 22 | 5 | 139 | 38 | 8 | 242 | 53,113 | 10,300 | 343,915 | 382 | 76 | 2,394 | 98·9 %_90 % |
| Moderate | 12–17 years | Tocantins | 138,998 | 11 | 3 | 97 | 20 | 5 | 171 | 27,398 | 6,088 | 234,387 | 198 | 45 | 1,653 | 98·9 %_90 % |
| Moderate | 18–59 years | Pernambuco | 5,490,560 | 45 | 9 | 298 | 81 | 16 | 533 | 117,350 | 22,428 | 819,984 | 821 | 164 | 5,412 | 98·9 %_90 % |
| Moderate | 18–59 years | Tocantins | 896,301 | 24 | 6 | 218 | 45 | 10 | 388 | 62,482 | 14,116 | 564,510 | 444 | 103 | 3,818 | 98·9 %_90 % |
| Moderate | 60+ years | Pernambuco | 869,490 | 36 | 6 | 415 | 66 | 10 | 735 | 52,084 | 8,087 | 547,272 | 629 | 95 | 6,832 | 98·9 %_90 % |
| Moderate | 60+ years | Tocantins | 122,565 | 17 | 3 | 300 | 32 | 6 | 530 | 27,125 | 5,087 | 421,655 | 301 | 55 | 4,840 | 98·9 %_90 % |

Supplementary table7 NNVs at sub-national level (Vimkunya)

| **Setting** | **Scenario** | **Region** | **Total vaccines** | **NNV (infection)** | **NNV (infection; 95% lo)** | **NNV (infection; 95% hi)** | **NNV (symptomatic)** | **NNV (symptomatic; 95% lo)** | **NNV (symptomatic; 95% hi)** | **NNV (Death)** | **NNV (Death; 95% lo)** | **NNV (Death; 95% hi)** | **NNV (DALY)** | **NNV (DALY; 95% lo)** | **NNV (DALY; 95% hi)** | **VEVC** |
| --- | --- | --- | --- | --- | --- | --- | --- | --- | --- | --- | --- | --- | --- | --- | --- | --- |
| High | 1–11 years | Alagoas | 52,717 | Inf | Inf | Inf | 15 | 3 | 129 | 9,638 | 2,148 | 85,350 | 90 | 20 | 793 | 0 %_10 % |
| High | 1–11 years | Ceará | 115,930 | Inf | Inf | Inf | 23 | 4 | 153 | 15,365 | 2,724 | 101,274 | 143 | 25 | 940 | 0 %_10 % |
| High | 1–11 years | Paraíba | 49,504 | Inf | Inf | Inf | 32 | 8 | 187 | 21,297 | 5,132 | 123,963 | 198 | 48 | 1,151 | 0 %_10 % |
| High | 1–11 years | Piauí | 45,738 | Inf | Inf | Inf | 19 | 5 | 148 | 12,371 | 2,987 | 98,339 | 115 | 28 | 913 | 0 %_10 % |
| High | 12–17 years | Alagoas | 31,096 | Inf | Inf | Inf | 16 | 4 | 171 | 28,279 | 6,091 | 295,679 | 175 | 38 | 1,835 | 0 %_10 % |
| High | 12–17 years | Ceará | 67,573 | Inf | Inf | Inf | 28 | 5 | 226 | 47,879 | 7,901 | 391,362 | 297 | 49 | 2,429 | 0 %_10 % |
| High | 12–17 years | Paraíba | 28,709 | Inf | Inf | Inf | 39 | 8 | 253 | 66,641 | 14,452 | 436,623 | 414 | 90 | 2,710 | 0 %_10 % |
| High | 12–17 years | Piauí | 27,894 | Inf | Inf | Inf | 24 | 5 | 227 | 40,655 | 9,425 | 392,614 | 252 | 58 | 2,437 | 0 %_10 % |
| High | 18–59 years | Alagoas | 201,650 | Inf | Inf | Inf | 27 | 5 | 397 | 67,633 | 11,710 | 1,013,656 | 320 | 56 | 4,768 | 0 %_10 % |
| High | 18–59 years | Ceará | 499,520 | Inf | Inf | Inf | 52 | 7 | 616 | 131,896 | 16,602 | 1,575,530 | 623 | 79 | 7,453 | 0 %_10 % |
| High | 18–59 years | Paraíba | 207,118 | Inf | Inf | Inf | 70 | 12 | 668 | 175,884 | 29,412 | 1,705,229 | 832 | 139 | 8,060 | 0 %_10 % |
| High | 18–59 years | Piauí | 192,626 | Inf | Inf | Inf | 43 | 7 | 688 | 109,391 | 17,775 | 1,738,125 | 517 | 84 | 8,301 | 0 %_10 % |
| High | 60+ years | Alagoas | 28,494 | Inf | Inf | Inf | 50 | 6 | 1,511 | 23,741 | 2,735 | 787,376 | 470 | 57 | 14,332 | 0 %_10 % |
| High | 60+ years | Ceará | 80,143 | Inf | Inf | Inf | 125 | 10 | 4,664 | 54,856 | 4,199 | 2,476,457 | 1,146 | 95 | 44,373 | 0 %_10 % |
| High | 60+ years | Paraíba | 35,889 | Inf | Inf | Inf | 153 | 18 | 3,486 | 66,764 | 7,318 | 1,703,731 | 1,403 | 161 | 32,688 | 0 %_10 % |
| High | 60+ years | Piauí | 32,227 | Inf | Inf | Inf | 99 | 11 | 4,708 | 45,125 | 4,478 | 2,442,110 | 913 | 96 | 44,615 | 0 %_10 % |
| Low | 1–11 years | Bahia | 167,710 | Inf | Inf | Inf | 377 | 78 | 1,360 | 249,983 | 51,632 | 901,927 | 2,325 | 481 | 8,379 | 0 %_10 % |
| Low | 1–11 years | Goiás | 94,119 | Inf | Inf | Inf | 425 | 136 | 1,610 | 281,588 | 90,443 | 1,067,564 | 2,617 | 841 | 9,913 | 0 %_10 % |
| Low | 1–11 years | Minas Gerais | 141,693 | Inf | Inf | Inf | 133 | 4 | 1,321 | 88,239 | 2,917 | 875,641 | 820 | 27 | 8,124 | 0 %_10 % |
| Low | 1–11 years | Rio Grande do Norte | 39,752 | Inf | Inf | Inf | 76 | 17 | 558 | 50,080 | 11,115 | 370,003 | 466 | 103 | 3,435 | 0 %_10 % |
| Low | 1–11 years | Sergipe | 35,934 | Inf | Inf | Inf | 71 | 15 | 319 | 47,120 | 9,974 | 211,287 | 438 | 93 | 1,964 | 0 %_10 % |
| Low | 12–17 years | Bahia | 100,511 | Inf | Inf | Inf | 433 | 83 | 1,926 | 749,437 | 143,722 | 3,329,513 | 4,651 | 892 | 20,663 | 0 %_10 % |
| Low | 12–17 years | Goiás | 52,263 | Inf | Inf | Inf | 483 | 146 | 2,107 | 834,985 | 251,975 | 3,643,486 | 5,182 | 1,564 | 22,612 | 0 %_10 % |
| Low | 12–17 years | Minas Gerais | 81,631 | Inf | Inf | Inf | 168 | 5 | 2,163 | 290,332 | 8,446 | 3,739,217 | 1,802 | 52 | 23,206 | 0 %_10 % |
| Low | 12–17 years | Rio Grande do Norte | 23,721 | Inf | Inf | Inf | 90 | 19 | 814 | 155,811 | 32,858 | 1,407,494 | 967 | 204 | 8,735 | 0 %_10 % |
| Low | 12–17 years | Sergipe | 21,541 | Inf | Inf | Inf | 82 | 17 | 402 | 140,936 | 29,362 | 695,044 | 875 | 182 | 4,313 | 0 %_10 % |
| Low | 18–59 years | Bahia | 734,611 | Inf | Inf | Inf | 720 | 123 | 4,457 | 1,812,120 | 307,239 | 11,346,857 | 8,566 | 1,459 | 53,629 | 0 %_10 % |
| Low | 18–59 years | Goiás | 415,700 | Inf | Inf | Inf | 736 | 186 | 4,805 | 1,855,900 | 465,481 | 12,330,223 | 8,765 | 2,207 | 57,902 | 0 %_10 % |
| Low | 18–59 years | Minas Gerais | 701,046 | Inf | Inf | Inf | 342 | 8 | 5,504 | 864,873 | 20,467 | 14,069,966 | 4,081 | 98 | 66,358 | 0 %_10 % |
| Low | 18–59 years | Rio Grande do Norte | 179,202 | Inf | Inf | Inf | 167 | 29 | 2,453 | 422,678 | 71,696 | 6,271,047 | 2,003 | 340 | 29,662 | 0 %_10 % |
| Low | 18–59 years | Sergipe | 150,723 | Inf | Inf | Inf | 135 | 24 | 821 | 341,493 | 60,034 | 2,091,831 | 1,612 | 283 | 9,870 | 0 %_10 % |
| Low | 60+ years | Bahia | 121,111 | Inf | Inf | Inf | 1,363 | 171 | 19,975 | 584,424 | 69,538 | 10,078,452 | 12,465 | 1,550 | 188,405 | 0 %_10 % |
| Low | 60+ years | Goiás | 57,694 | Inf | Inf | Inf | 1,242 | 240 | 20,601 | 584,349 | 107,743 | 11,158,693 | 11,564 | 2,218 | 196,684 | 0 %_10 % |
| Low | 60+ years | Minas Gerais | 132,262 | Inf | Inf | Inf | 862 | 12 | 29,210 | 401,859 | 4,966 | 15,527,615 | 8,013 | 109 | 278,015 | 0 %_10 % |
| Low | 60+ years | Rio Grande do Norte | 28,886 | Inf | Inf | Inf | 386 | 45 | 16,253 | 166,930 | 17,956 | 8,785,214 | 3,536 | 403 | 155,099 | 0 %_10 % |
| Low | 60+ years | Sergipe | 21,237 | Inf | Inf | Inf | 246 | 37 | 2,509 | 111,636 | 16,243 | 1,204,718 | 2,276 | 337 | 23,449 | 0 %_10 % |
| Moderate | 1–11 years | Pernambuco | 196,549 | Inf | Inf | Inf | 135 | 31 | 619 | 89,418 | 20,293 | 410,061 | 831 | 189 | 3,808 | 0 %_10 % |
| Moderate | 1–11 years | Tocantins | 35,864 | Inf | Inf | Inf | 81 | 19 | 515 | 53,617 | 12,860 | 341,249 | 498 | 120 | 3,169 | 0 %_10 % |
| Moderate | 12–17 years | Pernambuco | 114,240 | Inf | Inf | Inf | 162 | 34 | 839 | 279,376 | 58,985 | 1,451,176 | 1,734 | 366 | 9,006 | 0 %_10 % |
| Moderate | 12–17 years | Tocantins | 20,633 | Inf | Inf | Inf | 95 | 22 | 734 | 163,705 | 37,340 | 1,269,847 | 1,016 | 232 | 7,881 | 0 %_10 % |
| Moderate | 18–59 years | Pernambuco | 825,774 | Inf | Inf | Inf | 268 | 44 | 1,988 | 673,908 | 109,708 | 5,045,847 | 3,191 | 521 | 23,889 | 0 %_10 % |
| Moderate | 18–59 years | Tocantins | 133,047 | Inf | Inf | Inf | 152 | 31 | 1,902 | 384,725 | 78,184 | 4,868,528 | 1,814 | 370 | 22,937 | 0 %_10 % |
| Moderate | 60+ years | Pernambuco | 130,770 | Inf | Inf | Inf | 517 | 63 | 8,704 | 231,428 | 26,478 | 4,694,274 | 4,766 | 575 | 83,039 | 0 %_10 % |
| Moderate | 60+ years | Tocantins | 18,193 | Inf | Inf | Inf | 284 | 42 | 9,505 | 129,278 | 17,563 | 4,944,667 | 2,625 | 379 | 90,126 | 0 %_10 % |
| High | 1–11 years | Alagoas | 260,778 | Inf | Inf | Inf | 14 | 3 | 129 | 9,354 | 2,129 | 85,552 | 87 | 20 | 795 | 0 %_50 % |
| High | 1–11 years | Ceará | 627,876 | Inf | Inf | Inf | 25 | 5 | 168 | 16,346 | 3,039 | 111,631 | 152 | 28 | 1,036 | 0 %_50 % |
| High | 1–11 years | Paraíba | 280,516 | Inf | Inf | Inf | 36 | 8 | 198 | 23,671 | 5,338 | 131,270 | 220 | 50 | 1,218 | 0 %_50 % |
| High | 1–11 years | Piauí | 239,967 | Inf | Inf | Inf | 20 | 4 | 154 | 13,022 | 2,968 | 101,965 | 121 | 28 | 946 | 0 %_50 % |
| High | 12–17 years | Alagoas | 153,825 | Inf | Inf | Inf | 16 | 4 | 173 | 28,019 | 6,058 | 299,729 | 174 | 38 | 1,860 | 0 %_50 % |
| High | 12–17 years | Ceará | 365,978 | Inf | Inf | Inf | 30 | 5 | 245 | 52,163 | 8,813 | 423,379 | 324 | 55 | 2,628 | 0 %_50 % |
| High | 12–17 years | Paraíba | 162,680 | Inf | Inf | Inf | 43 | 9 | 282 | 74,330 | 15,464 | 487,070 | 461 | 96 | 3,023 | 0 %_50 % |
| High | 12–17 years | Piauí | 146,345 | Inf | Inf | Inf | 24 | 5 | 232 | 41,695 | 8,813 | 401,750 | 259 | 55 | 2,493 | 0 %_50 % |
| High | 18–59 years | Alagoas | 997,514 | Inf | Inf | Inf | 26 | 5 | 402 | 66,556 | 12,181 | 1,023,430 | 315 | 58 | 4,827 | 0 %_50 % |
| High | 18–59 years | Ceará | 2,705,400 | Inf | Inf | Inf | 56 | 7 | 693 | 141,927 | 18,353 | 1,772,378 | 670 | 87 | 8,386 | 0 %_50 % |
| High | 18–59 years | Paraíba | 1,173,650 | Inf | Inf | Inf | 78 | 13 | 724 | 195,636 | 32,608 | 1,850,198 | 925 | 155 | 8,727 | 0 %_50 % |
| High | 18–59 years | Piauí | 1,010,615 | Inf | Inf | Inf | 44 | 7 | 691 | 110,922 | 18,505 | 1,759,703 | 525 | 88 | 8,358 | 0 %_50 % |
| High | 60+ years | Alagoas | 140,951 | Inf | Inf | Inf | 48 | 7 | 1,523 | 22,643 | 2,900 | 780,897 | 449 | 60 | 14,404 | 0 %_50 % |
| High | 60+ years | Ceará | 434,053 | Inf | Inf | Inf | 134 | 12 | 5,007 | 58,612 | 4,745 | 2,639,750 | 1,231 | 105 | 47,582 | 0 %_50 % |
| High | 60+ years | Paraíba | 203,370 | Inf | Inf | Inf | 170 | 19 | 3,676 | 74,134 | 7,759 | 1,826,371 | 1,560 | 174 | 34,572 | 0 %_50 % |
| High | 60+ years | Piauí | 169,081 | Inf | Inf | Inf | 103 | 11 | 5,038 | 46,447 | 4,829 | 2,612,300 | 953 | 104 | 47,743 | 0 %_50 % |
| Low | 1–11 years | Bahia | 961,065 | Inf | Inf | Inf | 430 | 94 | 1,599 | 284,851 | 62,174 | 1,059,941 | 2,650 | 579 | 9,847 | 0 %_50 % |
| Low | 1–11 years | Goiás | 505,801 | Inf | Inf | Inf | 451 | 143 | 1,751 | 299,212 | 95,032 | 1,161,035 | 2,781 | 884 | 10,779 | 0 %_50 % |
| Low | 1–11 years | Minas Gerais | 1,100,714 | Inf | Inf | Inf | 212 | 6 | 1,917 | 140,539 | 4,078 | 1,271,225 | 1,306 | 38 | 11,795 | 0 %_50 % |
| Low | 1–11 years | Rio Grande do Norte | 222,882 | Inf | Inf | Inf | 86 | 20 | 628 | 57,060 | 13,061 | 416,068 | 531 | 122 | 3,863 | 0 %_50 % |
| Low | 1–11 years | Sergipe | 174,400 | Inf | Inf | Inf | 69 | 15 | 304 | 45,674 | 10,071 | 201,516 | 425 | 94 | 1,873 | 0 %_50 % |
| Low | 12–17 years | Bahia | 575,979 | Inf | Inf | Inf | 500 | 101 | 2,202 | 864,011 | 175,478 | 3,807,049 | 5,362 | 1,089 | 23,627 | 0 %_50 % |
| Low | 12–17 years | Goiás | 280,863 | Inf | Inf | Inf | 515 | 153 | 2,307 | 891,160 | 264,631 | 3,989,724 | 5,531 | 1,642 | 24,760 | 0 %_50 % |
| Low | 12–17 years | Minas Gerais | 634,133 | Inf | Inf | Inf | 263 | 7 | 2,917 | 455,609 | 11,609 | 5,043,141 | 2,828 | 72 | 31,298 | 0 %_50 % |
| Low | 12–17 years | Rio Grande do Norte | 132,998 | Inf | Inf | Inf | 104 | 22 | 917 | 180,505 | 38,004 | 1,585,189 | 1,120 | 236 | 9,838 | 0 %_50 % |
| Low | 12–17 years | Sergipe | 104,548 | Inf | Inf | Inf | 80 | 17 | 383 | 137,651 | 28,978 | 662,849 | 854 | 180 | 4,114 | 0 %_50 % |
| Low | 18–59 years | Bahia | 4,209,700 | Inf | Inf | Inf | 824 | 145 | 5,334 | 2,075,161 | 361,274 | 13,571,230 | 9,811 | 1,714 | 64,113 | 0 %_50 % |
| Low | 18–59 years | Goiás | 2,233,990 | Inf | Inf | Inf | 788 | 204 | 5,253 | 1,988,520 | 509,646 | 13,446,686 | 9,385 | 2,415 | 63,212 | 0 %_50 % |
| Low | 18–59 years | Minas Gerais | 5,445,941 | Inf | Inf | Inf | 534 | 11 | 8,174 | 1,346,793 | 27,278 | 20,866,454 | 6,373 | 130 | 98,625 | 0 %_50 % |
| Low | 18–59 years | Rio Grande do Norte | 1,004,748 | Inf | Inf | Inf | 191 | 32 | 2,698 | 483,171 | 81,251 | 6,900,183 | 2,285 | 386 | 32,596 | 0 %_50 % |
| Low | 18–59 years | Sergipe | 731,513 | Inf | Inf | Inf | 131 | 24 | 799 | 331,892 | 61,293 | 2,038,443 | 1,566 | 290 | 9,604 | 0 %_50 % |
| Low | 60+ years | Bahia | 694,029 | Inf | Inf | Inf | 1,578 | 200 | 22,829 | 682,094 | 81,599 | 11,345,577 | 14,453 | 1,812 | 214,731 | 0 %_50 % |
| Low | 60+ years | Goiás | 310,050 | Inf | Inf | Inf | 1,352 | 265 | 21,074 | 635,201 | 119,570 | 11,402,343 | 12,590 | 2,448 | 201,159 | 0 %_50 % |
| Low | 60+ years | Minas Gerais | 1,027,451 | Inf | Inf | Inf | 1,329 | 16 | 43,223 | 622,770 | 6,766 | 22,790,816 | 12,368 | 144 | 410,793 | 0 %_50 % |
| Low | 60+ years | Rio Grande do Norte | 161,960 | Inf | Inf | Inf | 435 | 50 | 17,383 | 189,689 | 20,458 | 9,239,167 | 3,988 | 453 | 165,410 | 0 %_50 % |
| Low | 60+ years | Sergipe | 103,072 | Inf | Inf | Inf | 239 | 36 | 2,410 | 107,509 | 15,825 | 1,155,243 | 2,208 | 333 | 22,518 | 0 %_50 % |
| Moderate | 1–11 years | Pernambuco | 809,462 | Inf | Inf | Inf | 111 | 26 | 530 | 73,921 | 17,184 | 351,519 | 687 | 160 | 3,264 | 0 %_50 % |
| Moderate | 1–11 years | Tocantins | 148,881 | Inf | Inf | Inf | 67 | 17 | 439 | 44,626 | 11,372 | 291,237 | 415 | 106 | 2,704 | 0 %_50 % |
| Moderate | 12–17 years | Pernambuco | 470,483 | Inf | Inf | Inf | 131 | 28 | 734 | 227,163 | 48,675 | 1,269,219 | 1,410 | 302 | 7,877 | 0 %_50 % |
| Moderate | 12–17 years | Tocantins | 85,653 | Inf | Inf | Inf | 78 | 19 | 606 | 135,490 | 32,229 | 1,048,280 | 841 | 200 | 6,506 | 0 %_50 % |
| Moderate | 18–59 years | Pernambuco | 3,400,843 | Inf | Inf | Inf | 217 | 38 | 1,704 | 546,314 | 93,760 | 4,340,276 | 2,584 | 445 | 20,505 | 0 %_50 % |
| Moderate | 18–59 years | Tocantins | 552,313 | Inf | Inf | Inf | 125 | 26 | 1,461 | 317,437 | 65,345 | 3,738,143 | 1,497 | 309 | 17,621 | 0 %_50 % |
| Moderate | 60+ years | Pernambuco | 538,561 | Inf | Inf | Inf | 418 | 52 | 6,818 | 187,541 | 22,040 | 3,606,992 | 3,856 | 474 | 64,835 | 0 %_50 % |
| Moderate | 60+ years | Tocantins | 75,526 | Inf | Inf | Inf | 236 | 37 | 7,176 | 106,536 | 15,835 | 3,818,037 | 2,178 | 339 | 68,305 | 0 %_50 % |
| High | 1–11 years | Alagoas | 466,969 | Inf | Inf | Inf | 14 | 3 | 130 | 9,334 | 2,129 | 86,035 | 87 | 20 | 799 | 0 %_90 % |
| High | 1–11 years | Ceará | 1,171,973 | Inf | Inf | Inf | 26 | 5 | 176 | 16,956 | 3,149 | 116,424 | 158 | 29 | 1,081 | 0 %_90 % |
| High | 1–11 years | Paraíba | 533,528 | Inf | Inf | Inf | 38 | 8 | 205 | 24,892 | 5,465 | 136,218 | 231 | 51 | 1,265 | 0 %_90 % |
| High | 1–11 years | Piauí | 441,713 | Inf | Inf | Inf | 20 | 4 | 160 | 13,316 | 2,953 | 106,120 | 124 | 27 | 985 | 0 %_90 % |
| High | 12–17 years | Alagoas | 275,450 | Inf | Inf | Inf | 16 | 4 | 173 | 27,934 | 6,075 | 299,821 | 173 | 38 | 1,861 | 0 %_90 % |
| High | 12–17 years | Ceará | 683,123 | Inf | Inf | Inf | 31 | 5 | 253 | 54,126 | 9,161 | 437,649 | 336 | 57 | 2,716 | 0 %_90 % |
| High | 12–17 years | Paraíba | 309,409 | Inf | Inf | Inf | 45 | 9 | 291 | 78,127 | 15,848 | 504,020 | 485 | 98 | 3,128 | 0 %_90 % |
| High | 12–17 years | Piauí | 269,381 | Inf | Inf | Inf | 25 | 5 | 239 | 42,487 | 8,770 | 413,910 | 264 | 54 | 2,569 | 0 %_90 % |
| High | 18–59 years | Alagoas | 1,786,221 | Inf | Inf | Inf | 26 | 5 | 401 | 65,888 | 12,327 | 1,022,048 | 312 | 58 | 4,826 | 0 %_90 % |
| High | 18–59 years | Ceará | 5,049,814 | Inf | Inf | Inf | 58 | 8 | 711 | 146,684 | 19,275 | 1,823,972 | 693 | 91 | 8,614 | 0 %_90 % |
| High | 18–59 years | Paraíba | 2,232,224 | Inf | Inf | Inf | 81 | 13 | 754 | 204,569 | 33,540 | 1,924,319 | 967 | 159 | 9,081 | 0 %_90 % |
| High | 18–59 years | Piauí | 1,860,260 | Inf | Inf | Inf | 45 | 7 | 706 | 112,695 | 18,775 | 1,799,408 | 533 | 89 | 8,536 | 0 %_90 % |
| High | 60+ years | Alagoas | 252,398 | Inf | Inf | Inf | 47 | 7 | 1,508 | 22,203 | 2,994 | 775,647 | 441 | 62 | 14,270 | 0 %_90 % |
| High | 60+ years | Ceará | 810,190 | Inf | Inf | Inf | 138 | 12 | 5,106 | 60,200 | 4,906 | 2,676,309 | 1,264 | 108 | 48,479 | 0 %_90 % |
| High | 60+ years | Paraíba | 386,799 | Inf | Inf | Inf | 177 | 20 | 3,792 | 76,520 | 8,183 | 1,883,103 | 1,621 | 183 | 35,664 | 0 %_90 % |
| High | 60+ years | Piauí | 311,230 | Inf | Inf | Inf | 104 | 12 | 5,095 | 46,622 | 4,968 | 2,688,027 | 959 | 107 | 48,428 | 0 %_90 % |
| Low | 1–11 years | Bahia | 1,836,096 | Inf | Inf | Inf | 456 | 101 | 1,695 | 302,370 | 66,793 | 1,123,963 | 2,813 | 622 | 10,442 | 0 %_90 % |
| Low | 1–11 years | Goiás | 940,954 | Inf | Inf | Inf | 465 | 148 | 1,822 | 308,075 | 97,830 | 1,207,769 | 2,863 | 910 | 11,213 | 0 %_90 % |
| Low | 1–11 years | Minas Gerais | 2,321,234 | Inf | Inf | Inf | 249 | 7 | 2,209 | 165,308 | 4,734 | 1,464,851 | 1,536 | 44 | 13,593 | 0 %_90 % |
| Low | 1–11 years | Rio Grande do Norte | 422,092 | Inf | Inf | Inf | 91 | 21 | 654 | 60,499 | 13,712 | 433,315 | 563 | 128 | 4,023 | 0 %_90 % |
| Low | 1–11 years | Sergipe | 309,353 | Inf | Inf | Inf | 68 | 15 | 303 | 44,935 | 10,010 | 200,911 | 418 | 93 | 1,868 | 0 %_90 % |
| Low | 12–17 years | Bahia | 1,100,397 | Inf | Inf | Inf | 531 | 109 | 2,331 | 918,037 | 188,735 | 4,030,553 | 5,697 | 1,171 | 25,014 | 0 %_90 % |
| Low | 12–17 years | Goiás | 522,497 | Inf | Inf | Inf | 530 | 159 | 2,415 | 917,210 | 274,237 | 4,176,154 | 5,692 | 1,702 | 25,917 | 0 %_90 % |
| Low | 12–17 years | Minas Gerais | 1,337,288 | Inf | Inf | Inf | 309 | 8 | 3,326 | 534,850 | 13,435 | 5,751,606 | 3,319 | 83 | 35,695 | 0 %_90 % |
| Low | 12–17 years | Rio Grande do Norte | 251,870 | Inf | Inf | Inf | 110 | 23 | 963 | 191,036 | 39,955 | 1,665,052 | 1,186 | 248 | 10,333 | 0 %_90 % |
| Low | 12–17 years | Sergipe | 185,449 | Inf | Inf | Inf | 78 | 17 | 381 | 135,454 | 28,803 | 658,567 | 841 | 179 | 4,087 | 0 %_90 % |
| Low | 18–59 years | Bahia | 8,042,554 | Inf | Inf | Inf | 873 | 156 | 5,674 | 2,197,598 | 388,282 | 14,449,668 | 10,388 | 1,842 | 68,235 | 0 %_90 % |
| Low | 18–59 years | Goiás | 4,155,940 | Inf | Inf | Inf | 813 | 211 | 5,435 | 2,051,262 | 527,094 | 13,896,114 | 9,683 | 2,498 | 65,391 | 0 %_90 % |
| Low | 18–59 years | Minas Gerais | 11,484,644 | Inf | Inf | Inf | 625 | 12 | 9,462 | 1,574,907 | 30,838 | 24,149,099 | 7,457 | 147 | 114,166 | 0 %_90 % |
| Low | 18–59 years | Rio Grande do Norte | 1,902,786 | Inf | Inf | Inf | 202 | 34 | 2,822 | 511,545 | 86,247 | 7,208,686 | 2,419 | 409 | 34,076 | 0 %_90 % |
| Low | 18–59 years | Sergipe | 1,297,570 | Inf | Inf | Inf | 129 | 24 | 784 | 326,114 | 61,216 | 2,001,647 | 1,539 | 289 | 9,420 | 0 %_90 % |
| Low | 60+ years | Bahia | 1,325,930 | Inf | Inf | Inf | 1,667 | 217 | 24,263 | 720,632 | 88,398 | 11,966,668 | 15,273 | 1,961 | 227,905 | 0 %_90 % |
| Low | 60+ years | Goiás | 576,792 | Inf | Inf | Inf | 1,390 | 276 | 21,326 | 653,863 | 124,569 | 11,609,269 | 12,940 | 2,553 | 203,764 | 0 %_90 % |
| Low | 60+ years | Minas Gerais | 2,166,736 | Inf | Inf | Inf | 1,539 | 18 | 50,805 | 723,660 | 7,760 | 26,468,778 | 14,332 | 166 | 481,855 | 0 %_90 % |
| Low | 60+ years | Rio Grande do Norte | 306,719 | Inf | Inf | Inf | 460 | 53 | 17,926 | 200,403 | 21,710 | 9,297,154 | 4,217 | 481 | 169,869 | 0 %_90 % |
| Low | 60+ years | Sergipe | 182,831 | Inf | Inf | Inf | 237 | 36 | 2,378 | 106,143 | 15,750 | 1,141,591 | 2,183 | 333 | 22,226 | 0 %_90 % |
| Moderate | 1–11 years | Pernambuco | 1,306,851 | Inf | Inf | Inf | 100 | 23 | 486 | 66,103 | 15,508 | 322,167 | 615 | 144 | 2,992 | 0 %_90 % |
| Moderate | 1–11 years | Tocantins | 241,606 | Inf | Inf | Inf | 61 | 16 | 390 | 40,281 | 10,451 | 258,320 | 374 | 97 | 2,398 | 0 %_90 % |
| Moderate | 12–17 years | Pernambuco | 759,581 | Inf | Inf | Inf | 117 | 25 | 670 | 202,666 | 43,995 | 1,158,566 | 1,258 | 273 | 7,190 | 0 %_90 % |
| Moderate | 12–17 years | Tocantins | 138,998 | Inf | Inf | Inf | 71 | 17 | 541 | 122,087 | 29,702 | 935,226 | 758 | 184 | 5,804 | 0 %_90 % |
| Moderate | 18–59 years | Pernambuco | 5,490,560 | Inf | Inf | Inf | 194 | 34 | 1,571 | 488,153 | 85,415 | 4,001,050 | 2,309 | 406 | 18,889 | 0 %_90 % |
| Moderate | 18–59 years | Tocantins | 896,301 | Inf | Inf | Inf | 113 | 24 | 1,293 | 285,408 | 59,861 | 3,306,524 | 1,346 | 283 | 15,596 | 0 %_90 % |
| Moderate | 60+ years | Pernambuco | 869,490 | Inf | Inf | Inf | 374 | 47 | 6,109 | 167,389 | 19,994 | 3,164,030 | 3,448 | 431 | 57,874 | 0 %_90 % |
| Moderate | 60+ years | Tocantins | 122,565 | Inf | Inf | Inf | 211 | 34 | 6,256 | 95,444 | 14,600 | 3,334,559 | 1,951 | 310 | 59,568 | 0 %_90 % |
| High | 1–11 years | Alagoas | 52,717 | 1 | 0 | 13 | 2 | 1 | 21 | 2,279 | 553 | 21,266 | 19 | 5 | 173 | 97·8 %_10 % |
| High | 1–11 years | Ceará | 115,930 | 3 | 1 | 31 | 5 | 1 | 44 | 4,887 | 872 | 38,149 | 42 | 8 | 325 | 97·8 %_10 % |
| High | 1–11 years | Paraíba | 49,504 | 5 | 1 | 36 | 7 | 2 | 53 | 6,769 | 1,489 | 45,230 | 58 | 13 | 390 | 97·8 %_10 % |
| High | 1–11 years | Piauí | 45,738 | 3 | 1 | 29 | 5 | 1 | 41 | 4,146 | 962 | 34,432 | 36 | 8 | 297 | 97·8 %_10 % |
| High | 12–17 years | Alagoas | 31,096 | 1 | 0 | 15 | 2 | 1 | 25 | 3,299 | 794 | 33,943 | 24 | 6 | 239 | 97·8 %_10 % |
| High | 12–17 years | Ceará | 67,573 | 4 | 1 | 37 | 6 | 1 | 55 | 8,442 | 1,415 | 80,215 | 59 | 10 | 546 | 97·8 %_10 % |
| High | 12–17 years | Paraíba | 28,709 | 5 | 1 | 43 | 8 | 2 | 65 | 11,507 | 2,284 | 94,006 | 82 | 17 | 650 | 97·8 %_10 % |
| High | 12–17 years | Piauí | 27,894 | 3 | 1 | 35 | 5 | 1 | 53 | 7,340 | 1,621 | 75,509 | 52 | 12 | 521 | 97·8 %_10 % |
| High | 18–59 years | Alagoas | 201,650 | 2 | 1 | 25 | 3 | 1 | 43 | 4,888 | 1,115 | 62,436 | 34 | 8 | 426 | 97·8 %_10 % |
| High | 18–59 years | Ceará | 499,520 | 5 | 1 | 67 | 9 | 1 | 110 | 14,255 | 2,027 | 179,371 | 95 | 14 | 1,135 | 97·8 %_10 % |
| High | 18–59 years | Paraíba | 207,118 | 7 | 1 | 74 | 13 | 2 | 122 | 18,976 | 3,372 | 198,310 | 129 | 24 | 1,267 | 97·8 %_10 % |
| High | 18–59 years | Piauí | 192,626 | 5 | 1 | 65 | 8 | 2 | 105 | 12,107 | 2,341 | 166,899 | 81 | 16 | 1,076 | 97·8 %_10 % |
| High | 60+ years | Alagoas | 28,494 | 2 | 1 | 43 | 4 | 1 | 77 | 3,799 | 667 | 70,903 | 40 | 8 | 717 | 97·8 %_10 % |
| High | 60+ years | Ceará | 80,143 | 8 | 1 | 158 | 14 | 2 | 282 | 10,103 | 1,088 | 213,588 | 130 | 15 | 2,596 | 97·8 %_10 % |
| High | 60+ years | Paraíba | 35,889 | 10 | 1 | 158 | 18 | 3 | 276 | 12,971 | 1,811 | 196,866 | 167 | 24 | 2,549 | 97·8 %_10 % |
| High | 60+ years | Piauí | 32,227 | 7 | 1 | 154 | 12 | 2 | 281 | 8,530 | 1,240 | 205,616 | 111 | 17 | 2,557 | 97·8 %_10 % |
| Low | 1–11 years | Bahia | 167,710 | 61 | 7 | 436 | 91 | 11 | 522 | 83,504 | 11,500 | 413,774 | 715 | 96 | 3,646 | 97·8 %_10 % |
| Low | 1–11 years | Goiás | 94,119 | 92 | 22 | 542 | 127 | 31 | 649 | 110,862 | 29,242 | 515,400 | 954 | 246 | 4,522 | 97·8 %_10 % |
| Low | 1–11 years | Minas Gerais | 141,693 | 10 | 0 | 266 | 17 | 0 | 407 | 18,080 | 382 | 337,087 | 148 | 3 | 2,927 | 97·8 %_10 % |
| Low | 1–11 years | Rio Grande do Norte | 39,752 | 10 | 2 | 116 | 15 | 3 | 165 | 14,573 | 2,926 | 140,519 | 123 | 25 | 1,207 | 97·8 %_10 % |
| Low | 1–11 years | Sergipe | 35,934 | 8 | 2 | 54 | 13 | 3 | 74 | 13,205 | 2,597 | 67,946 | 110 | 21 | 572 | 97·8 %_10 % |
| Low | 12–17 years | Bahia | 100,511 | 64 | 7 | 514 | 98 | 11 | 650 | 139,875 | 14,909 | 994,744 | 994 | 113 | 6,633 | 97·8 %_10 % |
| Low | 12–17 years | Goiás | 52,263 | 97 | 22 | 625 | 136 | 31 | 776 | 203,452 | 45,766 | 1,221,539 | 1,392 | 321 | 7,988 | 97·8 %_10 % |
| Low | 12–17 years | Minas Gerais | 81,631 | 11 | 0 | 311 | 18 | 0 | 535 | 24,417 | 469 | 798,054 | 180 | 4 | 5,402 | 97·8 %_10 % |
| Low | 12–17 years | Rio Grande do Norte | 23,721 | 10 | 2 | 140 | 17 | 3 | 205 | 23,175 | 4,164 | 297,460 | 166 | 31 | 2,035 | 97·8 %_10 % |
| Low | 12–17 years | Sergipe | 21,541 | 9 | 2 | 61 | 14 | 3 | 84 | 20,182 | 3,689 | 120,669 | 143 | 26 | 839 | 97·8 %_10 % |
| Low | 18–59 years | Bahia | 734,611 | 91 | 10 | 856 | 149 | 17 | 1,173 | 232,366 | 23,250 | 2,097,581 | 1,559 | 171 | 12,570 | 97·8 %_10 % |
| Low | 18–59 years | Goiás | 415,700 | 128 | 29 | 1,011 | 190 | 42 | 1,404 | 324,428 | 67,130 | 2,615,870 | 2,033 | 444 | 15,236 | 97·8 %_10 % |
| Low | 18–59 years | Minas Gerais | 701,046 | 19 | 0 | 623 | 34 | 1 | 1,064 | 46,656 | 665 | 1,809,337 | 337 | 5 | 11,260 | 97·8 %_10 % |
| Low | 18–59 years | Rio Grande do Norte | 179,202 | 16 | 3 | 261 | 26 | 4 | 422 | 39,325 | 6,271 | 687,886 | 270 | 45 | 4,355 | 97·8 %_10 % |
| Low | 18–59 years | Sergipe | 150,723 | 13 | 2 | 95 | 21 | 4 | 136 | 31,654 | 5,427 | 213,364 | 216 | 38 | 1,396 | 97·8 %_10 % |
| Low | 60+ years | Bahia | 121,111 | 114 | 10 | 1,659 | 191 | 16 | 2,697 | 140,514 | 13,955 | 1,748,369 | 1,814 | 155 | 25,038 | 97·8 %_10 % |
| Low | 60+ years | Goiás | 57,694 | 156 | 29 | 1,922 | 242 | 42 | 3,107 | 171,180 | 32,338 | 2,178,937 | 2,303 | 407 | 29,402 | 97·8 %_10 % |
| Low | 60+ years | Minas Gerais | 132,262 | 25 | 0 | 1,334 | 43 | 1 | 2,634 | 42,011 | 552 | 1,845,933 | 413 | 5 | 24,702 | 97·8 %_10 % |
| Low | 60+ years | Rio Grande do Norte | 28,886 | 21 | 3 | 592 | 36 | 5 | 1,102 | 28,949 | 3,815 | 828,749 | 346 | 46 | 10,160 | 97·8 %_10 % |
| Low | 60+ years | Sergipe | 21,237 | 15 | 2 | 151 | 26 | 4 | 235 | 21,647 | 3,401 | 182,889 | 250 | 38 | 2,201 | 97·8 %_10 % |
| Moderate | 1–11 years | Pernambuco | 196,549 | 24 | 5 | 148 | 36 | 7 | 198 | 31,656 | 6,460 | 164,169 | 272 | 55 | 1,424 | 97·8 %_10 % |
| Moderate | 1–11 years | Tocantins | 35,864 | 11 | 3 | 101 | 18 | 4 | 148 | 16,589 | 3,740 | 126,313 | 139 | 31 | 1,085 | 97·8 %_10 % |
| Moderate | 12–17 years | Pernambuco | 114,240 | 25 | 5 | 174 | 39 | 7 | 236 | 56,221 | 10,054 | 350,148 | 394 | 72 | 2,374 | 97·8 %_10 % |
| Moderate | 12–17 years | Tocantins | 20,633 | 12 | 3 | 120 | 19 | 4 | 177 | 26,846 | 5,489 | 249,069 | 189 | 40 | 1,731 | 97·8 %_10 % |
| Moderate | 18–59 years | Pernambuco | 825,774 | 35 | 6 | 291 | 56 | 10 | 428 | 90,178 | 14,628 | 725,421 | 590 | 100 | 4,495 | 97·8 %_10 % |
| Moderate | 18–59 years | Tocantins | 133,047 | 16 | 3 | 203 | 27 | 5 | 313 | 41,783 | 7,956 | 495,116 | 280 | 55 | 3,189 | 97·8 %_10 % |
| Moderate | 60+ years | Pernambuco | 130,770 | 44 | 7 | 576 | 77 | 10 | 902 | 54,835 | 7,428 | 655,014 | 724 | 96 | 8,412 | 97·8 %_10 % |
| Moderate | 60+ years | Tocantins | 18,193 | 20 | 3 | 407 | 35 | 6 | 704 | 27,302 | 4,454 | 531,097 | 331 | 54 | 6,412 | 97·8 %_10 % |
| High | 1–11 years | Alagoas | 260,778 | 2 | 0 | 18 | 3 | 1 | 30 | 3,211 | 768 | 31,795 | 26 | 6 | 254 | 97·8 %_50 % |
| High | 1–11 years | Ceará | 627,876 | 4 | 1 | 38 | 7 | 1 | 61 | 7,107 | 1,266 | 54,898 | 60 | 11 | 463 | 97·8 %_50 % |
| High | 1–11 years | Paraíba | 280,516 | 6 | 1 | 47 | 11 | 2 | 73 | 10,178 | 2,099 | 64,275 | 86 | 18 | 549 | 97·8 %_50 % |
| High | 1–11 years | Piauí | 239,967 | 4 | 1 | 37 | 6 | 1 | 56 | 5,734 | 1,262 | 49,197 | 48 | 11 | 418 | 97·8 %_50 % |
| High | 12–17 years | Alagoas | 153,825 | 2 | 0 | 18 | 3 | 1 | 31 | 3,934 | 961 | 42,480 | 29 | 7 | 303 | 97·8 %_50 % |
| High | 12–17 years | Ceará | 365,978 | 5 | 1 | 43 | 8 | 1 | 72 | 10,946 | 1,835 | 102,982 | 78 | 13 | 709 | 97·8 %_50 % |
| High | 12–17 years | Paraíba | 162,680 | 7 | 1 | 52 | 11 | 2 | 84 | 15,315 | 2,932 | 119,567 | 111 | 22 | 833 | 97·8 %_50 % |
| High | 12–17 years | Piauí | 146,345 | 4 | 1 | 42 | 6 | 1 | 67 | 8,981 | 1,920 | 94,180 | 64 | 14 | 655 | 97·8 %_50 % |
| High | 18–59 years | Alagoas | 997,514 | 3 | 1 | 42 | 6 | 1 | 74 | 8,274 | 1,765 | 101,502 | 60 | 13 | 715 | 97·8 %_50 % |
| High | 18–59 years | Ceará | 2,705,400 | 8 | 1 | 91 | 15 | 2 | 165 | 22,392 | 3,293 | 257,092 | 155 | 24 | 1,675 | 97·8 %_50 % |
| High | 18–59 years | Paraíba | 1,173,650 | 12 | 2 | 107 | 21 | 4 | 182 | 31,171 | 5,411 | 282,756 | 218 | 40 | 1,854 | 97·8 %_50 % |
| High | 18–59 years | Piauí | 1,010,615 | 7 | 1 | 89 | 12 | 2 | 149 | 17,822 | 3,363 | 227,613 | 123 | 24 | 1,498 | 97·8 %_50 % |
| High | 60+ years | Alagoas | 140,951 | 3 | 1 | 48 | 5 | 1 | 87 | 4,438 | 842 | 80,909 | 45 | 9 | 808 | 97·8 %_50 % |
| High | 60+ years | Ceará | 434,053 | 9 | 1 | 176 | 17 | 2 | 331 | 12,803 | 1,489 | 247,776 | 159 | 19 | 3,036 | 97·8 %_50 % |
| High | 60+ years | Paraíba | 203,370 | 13 | 2 | 184 | 23 | 3 | 325 | 17,107 | 2,444 | 236,311 | 214 | 31 | 3,012 | 97·8 %_50 % |
| High | 60+ years | Piauí | 169,081 | 8 | 1 | 174 | 14 | 2 | 322 | 10,405 | 1,577 | 239,354 | 130 | 21 | 2,922 | 97·8 %_50 % |
| Low | 1–11 years | Bahia | 961,065 | 86 | 11 | 567 | 139 | 19 | 743 | 129,014 | 19,675 | 600,399 | 1,099 | 163 | 5,263 | 97·8 %_50 % |
| Low | 1–11 years | Goiás | 505,801 | 115 | 29 | 655 | 172 | 43 | 867 | 154,264 | 42,120 | 700,146 | 1,315 | 352 | 6,115 | 97·8 %_50 % |
| Low | 1–11 years | Minas Gerais | 1,100,714 | 23 | 0 | 476 | 40 | 1 | 763 | 43,622 | 846 | 647,337 | 352 | 7 | 5,583 | 97·8 %_50 % |
| Low | 1–11 years | Rio Grande do Norte | 222,882 | 14 | 3 | 151 | 23 | 4 | 234 | 22,896 | 4,533 | 205,839 | 190 | 38 | 1,747 | 97·8 %_50 % |
| Low | 1–11 years | Sergipe | 174,400 | 10 | 2 | 63 | 17 | 3 | 96 | 17,785 | 3,619 | 92,011 | 146 | 29 | 763 | 97·8 %_50 % |
| Low | 12–17 years | Bahia | 575,979 | 85 | 10 | 644 | 140 | 17 | 870 | 195,618 | 22,349 | 1,307,693 | 1,405 | 170 | 8,816 | 97·8 %_50 % |
| Low | 12–17 years | Goiás | 280,863 | 116 | 27 | 737 | 176 | 41 | 1,003 | 259,897 | 59,569 | 1,553,485 | 1,791 | 422 | 10,261 | 97·8 %_50 % |
| Low | 12–17 years | Minas Gerais | 634,133 | 22 | 0 | 543 | 38 | 1 | 912 | 49,807 | 844 | 1,336,146 | 370 | 7 | 9,146 | 97·8 %_50 % |
| Low | 12–17 years | Rio Grande do Norte | 132,998 | 14 | 2 | 173 | 23 | 4 | 274 | 32,279 | 5,692 | 393,304 | 233 | 43 | 2,710 | 97·8 %_50 % |
| Low | 12–17 years | Sergipe | 104,548 | 10 | 2 | 66 | 17 | 3 | 102 | 23,978 | 4,508 | 144,283 | 172 | 33 | 1,010 | 97·8 %_50 % |
| Low | 18–59 years | Bahia | 4,209,700 | 164 | 24 | 1,214 | 274 | 41 | 1,751 | 422,268 | 56,140 | 3,059,241 | 2,848 | 418 | 18,640 | 97·8 %_50 % |
| Low | 18–59 years | Goiás | 2,233,990 | 194 | 49 | 1,344 | 303 | 76 | 1,963 | 503,546 | 117,755 | 3,565,999 | 3,209 | 793 | 21,115 | 97·8 %_50 % |
| Low | 18–59 years | Minas Gerais | 5,445,941 | 65 | 1 | 1,162 | 115 | 2 | 2,086 | 158,719 | 2,701 | 3,426,909 | 1,150 | 21 | 21,756 | 97·8 %_50 % |
| Low | 18–59 years | Rio Grande do Norte | 1,004,748 | 28 | 5 | 373 | 50 | 9 | 643 | 72,070 | 12,293 | 1,017,508 | 507 | 91 | 6,568 | 97·8 %_50 % |
| Low | 18–59 years | Sergipe | 731,513 | 20 | 4 | 129 | 35 | 7 | 211 | 50,533 | 9,422 | 319,104 | 355 | 68 | 2,135 | 97·8 %_50 % |
| Low | 60+ years | Bahia | 694,029 | 145 | 14 | 2,006 | 257 | 23 | 3,347 | 192,564 | 21,207 | 2,164,322 | 2,438 | 226 | 30,918 | 97·8 %_50 % |
| Low | 60+ years | Goiás | 310,050 | 182 | 35 | 2,190 | 300 | 55 | 3,598 | 219,350 | 43,868 | 2,503,227 | 2,861 | 529 | 33,952 | 97·8 %_50 % |
| Low | 60+ years | Minas Gerais | 1,027,451 | 45 | 1 | 2,109 | 82 | 1 | 4,209 | 81,374 | 1,044 | 3,026,476 | 791 | 10 | 39,382 | 97·8 %_50 % |
| Low | 60+ years | Rio Grande do Norte | 161,960 | 27 | 4 | 704 | 48 | 6 | 1,300 | 39,385 | 5,427 | 975,212 | 458 | 62 | 12,014 | 97·8 %_50 % |
| Low | 60+ years | Sergipe | 103,072 | 17 | 3 | 158 | 30 | 5 | 259 | 25,404 | 4,133 | 208,278 | 283 | 45 | 2,424 | 97·8 %_50 % |
| Moderate | 1–11 years | Pernambuco | 809,462 | 23 | 5 | 139 | 37 | 8 | 215 | 34,312 | 7,287 | 184,833 | 292 | 62 | 1,588 | 97·8 %_50 % |
| Moderate | 1–11 years | Tocantins | 148,881 | 11 | 3 | 96 | 19 | 4 | 155 | 18,984 | 4,498 | 138,543 | 158 | 37 | 1,169 | 97·8 %_50 % |
| Moderate | 12–17 years | Pernambuco | 470,483 | 23 | 5 | 156 | 38 | 7 | 246 | 54,360 | 10,217 | 356,789 | 386 | 74 | 2,451 | 97·8 %_50 % |
| Moderate | 12–17 years | Tocantins | 85,653 | 11 | 3 | 106 | 19 | 4 | 174 | 26,960 | 5,786 | 243,273 | 192 | 42 | 1,698 | 97·8 %_50 % |
| Moderate | 18–59 years | Pernambuco | 3,400,843 | 40 | 8 | 296 | 69 | 13 | 497 | 105,807 | 19,002 | 805,966 | 714 | 134 | 5,144 | 97·8 %_50 % |
| Moderate | 18–59 years | Tocantins | 552,313 | 20 | 5 | 208 | 36 | 8 | 354 | 52,775 | 11,307 | 540,271 | 364 | 80 | 3,553 | 97·8 %_50 % |
| Moderate | 60+ years | Pernambuco | 538,561 | 40 | 6 | 496 | 71 | 10 | 826 | 52,755 | 7,727 | 591,374 | 675 | 97 | 7,681 | 97·8 %_50 % |
| Moderate | 60+ years | Tocantins | 75,526 | 18 | 3 | 350 | 33 | 6 | 605 | 26,896 | 4,800 | 464,158 | 315 | 55 | 5,520 | 97·8 %_50 % |
| High | 1–11 years | Alagoas | 466,969 | 2 | 1 | 23 | 4 | 1 | 40 | 4,331 | 1,006 | 43,948 | 34 | 8 | 345 | 97·8 %_90 % |
| High | 1–11 years | Ceará | 1,171,973 | 5 | 1 | 45 | 10 | 2 | 80 | 9,570 | 1,694 | 73,646 | 79 | 14 | 614 | 97·8 %_90 % |
| High | 1–11 years | Paraíba | 533,528 | 8 | 2 | 57 | 14 | 3 | 95 | 13,926 | 2,807 | 86,303 | 117 | 24 | 728 | 97·8 %_90 % |
| High | 1–11 years | Piauí | 441,713 | 4 | 1 | 43 | 8 | 2 | 72 | 7,574 | 1,635 | 65,929 | 63 | 14 | 552 | 97·8 %_90 % |
| High | 12–17 years | Alagoas | 275,450 | 2 | 1 | 22 | 4 | 1 | 39 | 4,684 | 1,136 | 51,394 | 34 | 8 | 369 | 97·8 %_90 % |
| High | 12–17 years | Ceará | 683,123 | 5 | 1 | 49 | 10 | 2 | 88 | 13,296 | 2,234 | 123,861 | 96 | 16 | 861 | 97·8 %_90 % |
| High | 12–17 years | Paraíba | 309,409 | 8 | 2 | 61 | 14 | 3 | 104 | 18,915 | 3,556 | 144,648 | 139 | 27 | 1,017 | 97·8 %_90 % |
| High | 12–17 years | Piauí | 269,381 | 4 | 1 | 48 | 8 | 2 | 81 | 10,687 | 2,263 | 112,297 | 77 | 17 | 788 | 97·8 %_90 % |
| High | 18–59 years | Alagoas | 1,786,221 | 5 | 1 | 60 | 9 | 2 | 110 | 12,515 | 2,617 | 145,557 | 92 | 20 | 1,043 | 97·8 %_90 % |
| High | 18–59 years | Ceará | 5,049,814 | 12 | 2 | 117 | 22 | 3 | 220 | 31,380 | 4,708 | 333,370 | 222 | 35 | 2,211 | 97·8 %_90 % |
| High | 18–59 years | Paraíba | 2,232,224 | 18 | 3 | 142 | 32 | 6 | 249 | 44,737 | 7,878 | 374,731 | 320 | 59 | 2,505 | 97·8 %_90 % |
| High | 18–59 years | Piauí | 1,860,260 | 9 | 2 | 114 | 17 | 3 | 197 | 24,167 | 4,554 | 291,376 | 171 | 33 | 1,958 | 97·8 %_90 % |
| High | 60+ years | Alagoas | 252,398 | 3 | 1 | 54 | 5 | 1 | 98 | 5,235 | 1,021 | 92,247 | 52 | 11 | 904 | 97·8 %_90 % |
| High | 60+ years | Ceará | 810,190 | 10 | 1 | 192 | 20 | 2 | 374 | 15,503 | 1,880 | 282,545 | 187 | 23 | 3,433 | 97·8 %_90 % |
| High | 60+ years | Paraíba | 386,799 | 15 | 2 | 206 | 27 | 4 | 369 | 21,113 | 3,080 | 272,656 | 255 | 37 | 3,415 | 97·8 %_90 % |
| High | 60+ years | Piauí | 311,230 | 9 | 1 | 192 | 16 | 3 | 357 | 12,294 | 1,921 | 269,589 | 148 | 24 | 3,238 | 97·8 %_90 % |
| Low | 1–11 years | Bahia | 1,836,096 | 108 | 15 | 668 | 185 | 26 | 941 | 175,503 | 28,418 | 775,364 | 1,485 | 235 | 6,755 | 97·8 %_90 % |
| Low | 1–11 years | Goiás | 940,954 | 136 | 35 | 751 | 218 | 56 | 1,085 | 201,008 | 55,862 | 895,131 | 1,698 | 463 | 7,762 | 97·8 %_90 % |
| Low | 1–11 years | Minas Gerais | 2,321,234 | 36 | 1 | 619 | 64 | 1 | 1,070 | 71,357 | 1,386 | 936,456 | 573 | 11 | 7,995 | 97·8 %_90 % |
| Low | 1–11 years | Rio Grande do Norte | 422,092 | 17 | 3 | 181 | 31 | 6 | 304 | 31,739 | 6,255 | 275,343 | 261 | 52 | 2,314 | 97·8 %_90 % |
| Low | 1–11 years | Sergipe | 309,353 | 12 | 2 | 73 | 22 | 4 | 121 | 23,178 | 4,743 | 119,777 | 187 | 38 | 981 | 97·8 %_90 % |
| Low | 12–17 years | Bahia | 1,100,397 | 103 | 13 | 736 | 178 | 23 | 1,071 | 247,666 | 29,454 | 1,588,248 | 1,790 | 225 | 10,791 | 97·8 %_90 % |
| Low | 12–17 years | Goiás | 522,497 | 132 | 32 | 819 | 215 | 51 | 1,215 | 314,579 | 72,955 | 1,857,899 | 2,182 | 521 | 12,361 | 97·8 %_90 % |
| Low | 12–17 years | Minas Gerais | 1,337,288 | 31 | 1 | 682 | 55 | 1 | 1,224 | 73,096 | 1,209 | 1,765,689 | 545 | 10 | 12,196 | 97·8 %_90 % |
| Low | 12–17 years | Rio Grande do Norte | 251,870 | 17 | 3 | 200 | 30 | 5 | 339 | 40,634 | 7,141 | 480,364 | 296 | 54 | 3,331 | 97·8 %_90 % |
| Low | 12–17 years | Sergipe | 185,449 | 12 | 2 | 74 | 21 | 4 | 121 | 28,202 | 5,366 | 169,670 | 204 | 39 | 1,197 | 97·8 %_90 % |
| Low | 18–59 years | Bahia | 8,042,554 | 232 | 39 | 1,508 | 399 | 69 | 2,289 | 608,063 | 92,497 | 3,923,247 | 4,136 | 692 | 24,215 | 97·8 %_90 % |
| Low | 18–59 years | Goiás | 4,155,940 | 260 | 69 | 1,632 | 422 | 112 | 2,505 | 686,549 | 170,189 | 4,457,758 | 4,439 | 1,160 | 26,751 | 97·8 %_90 % |
| Low | 18–59 years | Minas Gerais | 11,484,644 | 116 | 2 | 1,622 | 209 | 4 | 3,012 | 287,436 | 5,508 | 4,811,803 | 2,090 | 44 | 31,064 | 97·8 %_90 % |
| Low | 18–59 years | Rio Grande do Norte | 1,902,786 | 42 | 8 | 485 | 77 | 14 | 869 | 107,782 | 19,242 | 1,339,743 | 770 | 145 | 8,787 | 97·8 %_90 % |
| Low | 18–59 years | Sergipe | 1,297,570 | 29 | 6 | 172 | 51 | 10 | 290 | 72,153 | 13,947 | 428,941 | 515 | 102 | 2,914 | 97·8 %_90 % |
| Low | 60+ years | Bahia | 1,325,930 | 172 | 17 | 2,256 | 314 | 30 | 3,849 | 240,166 | 28,309 | 2,510,081 | 2,983 | 294 | 35,506 | 97·8 %_90 % |
| Low | 60+ years | Goiás | 576,792 | 206 | 41 | 2,405 | 354 | 66 | 4,042 | 266,102 | 55,028 | 2,823,224 | 3,379 | 642 | 38,043 | 97·8 %_90 % |
| Low | 60+ years | Minas Gerais | 2,166,736 | 62 | 1 | 2,560 | 116 | 1 | 5,179 | 115,953 | 1,576 | 3,799,636 | 1,111 | 14 | 48,466 | 97·8 %_90 % |
| Low | 60+ years | Rio Grande do Norte | 306,719 | 32 | 4 | 789 | 58 | 8 | 1,466 | 48,929 | 6,980 | 1,102,987 | 553 | 76 | 13,555 | 97·8 %_90 % |
| Low | 60+ years | Sergipe | 182,831 | 18 | 3 | 169 | 33 | 5 | 287 | 29,717 | 4,987 | 236,863 | 320 | 52 | 2,691 | 97·8 %_90 % |
| Moderate | 1–11 years | Pernambuco | 1,306,851 | 24 | 5 | 142 | 41 | 8 | 240 | 39,434 | 8,447 | 213,050 | 331 | 71 | 1,810 | 97·8 %_90 % |
| Moderate | 1–11 years | Tocantins | 241,606 | 12 | 3 | 100 | 22 | 5 | 173 | 22,508 | 5,401 | 160,998 | 184 | 44 | 1,338 | 97·8 %_90 % |
| Moderate | 12–17 years | Pernambuco | 759,581 | 23 | 5 | 153 | 41 | 8 | 263 | 56,735 | 10,850 | 376,737 | 406 | 80 | 2,609 | 97·8 %_90 % |
| Moderate | 12–17 years | Tocantins | 138,998 | 12 | 3 | 105 | 21 | 5 | 184 | 28,721 | 6,303 | 253,659 | 207 | 46 | 1,783 | 97·8 %_90 % |
| Moderate | 18–59 years | Pernambuco | 5,490,560 | 48 | 10 | 326 | 85 | 17 | 578 | 125,635 | 23,603 | 908,784 | 869 | 170 | 5,916 | 97·8 %_90 % |
| Moderate | 18–59 years | Tocantins | 896,301 | 25 | 6 | 235 | 46 | 11 | 417 | 65,542 | 14,633 | 615,718 | 463 | 106 | 4,129 | 97·8 %_90 % |
| Moderate | 60+ years | Pernambuco | 869,490 | 39 | 6 | 469 | 72 | 11 | 824 | 55,288 | 8,490 | 597,671 | 684 | 102 | 7,657 | 97·8 %_90 % |
| Moderate | 60+ years | Tocantins | 122,565 | 18 | 3 | 333 | 34 | 6 | 588 | 28,494 | 5,311 | 458,381 | 323 | 59 | 5,373 | 97·8 %_90 % |

##### References

[1     Kang H, Lim A, Auzenbergs M, *et al.* Global, regional and national burden of chikungunya: force of infection mapping and spatial modelling study. *BMJ Glob Health* 2025; **10**. DOI:10.1136/bmjgh-2024-018598.](https://sciwheel.com/work/bibliography/18332060)

[2     Bustos Carrillo F, Collado D, Sanchez N, *et al.* Epidemiological Evidence for Lineage-Specific Differences in the Risk of Inapparent Chikungunya Virus Infection. *J Virol* 2019; **93**. DOI:10.1128/JVI.01622-18.](https://sciwheel.com/work/bibliography/6089155)

[3     Kang H, Auzenbergs M, Clapham H, *et al.* Chikungunya seroprevalence, force of infection, and prevalence of chronic disability after infection in endemic and epidemic settings: a systematic review, meta-analysis, and modelling study. *Lancet Infect Dis* 2024; **24**: 488–503.](https://sciwheel.com/work/bibliography/16033691)

[4     SINANWEB - Página inicial. 2024. http://portalsinan.saude.gov.br/ (accessed Oct 1, 2024).](https://sciwheel.com/work/bibliography/16987000)

[5     O’Driscoll M, Salje H, Chang AY, Watson H. Arthralgia resolution rate following chikungunya virus infection. *Int J Infect Dis* 2021; **112**: 1–7.](https://sciwheel.com/work/bibliography/12383358)

[6     Global Burden of Disease Study 2017 (GBD 2017) Disability Weights | GHDx. 2018. https://ghdx.healthdata.org/record/ihme-data/gbd-2017-disability-weights (accessed Aug 11, 2022).](https://sciwheel.com/work/bibliography/13449189)

[7     2.3. Calculating disability weights — SPHeP-NCDs  documentation. 2019. http://oecdpublichealthexplorer.org/ncd-doc/disease/disability_weights.html (accessed July 15, 2024).](https://sciwheel.com/work/bibliography/16665325)

[8     Unable to find information for 13353318. .](https://sciwheel.com/work/bibliography/13353318)

[9     Chikungunya | CDC Yellow Book 2024. https://wwwnc.cdc.gov/travel/yellowbook/2024/infections-diseases/chikungunya#:~:text=Approximately%203%25%E2%80%9328%25%20of,C%5D)%20and%20joint%20pains. (accessed Oct 15, 2023).](https://sciwheel.com/work/bibliography/15507432)

[10    United Nations Department of Economic and Social Affairs, Population Division. 2019 World Population Prospects. United Nations: https://population.un.org/wpp, 2019.](https://sciwheel.com/work/bibliography/8436701)
